# Copper-catalyzed synthesis of allenylboronic acids. Access to sterically encumbered homopropargylic alcohols and amines by propargylboration†
†Electronic supplementary information (ESI) available: Detailed experimental procedures and compound characterization data are given. CCDC 1550097. For ESI and crystallographic data in CIF or other electronic format see DOI: 10.1039/c7sc05123a


**DOI:** 10.1039/c7sc05123a

**Published:** 2018-02-19

**Authors:** Jian Zhao, Sybrand J. T. Jonker, Denise N. Meyer, Göran Schulz, C. Duc Tran, Lars Eriksson, Kálmán J. Szabó

**Affiliations:** a Department of Organic Chemistry , Stockholm University , SE-106 91 Stockholm , Sweden . Email: kalman.j.szabo@su.se; b Department of Materials and Environmental Chemistry , Stockholm University , SE-106 91 Stockholm , Sweden

## Abstract

Tri- and tetrasubstituted allenylboronic acids were prepared *via* a new versatile copper-catalyzed methodology. The densely functionalized allenylboronic acids readily undergo propargylboration reactions with ketones and imines without any additives. Catalytic asymmetric propargylborylation of ketones is demonstrated with high stereoselectivity allowing for the synthesis of highly enantioenriched tertiary homopropargyl alcohols. The reaction is suitable for kinetic resolution of racemic allenylboronic acids affording alkynes with adjacent quaternary stereocenters.

## Abstract 2

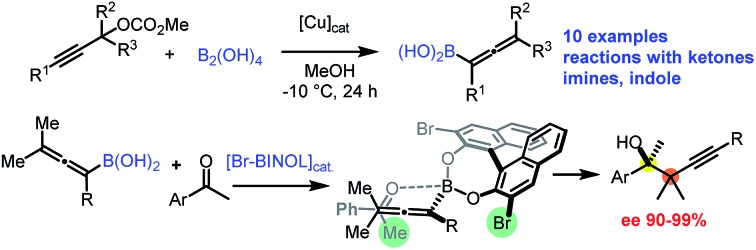
Synthesis and application of allenylboronic acids is presented. The successful synthetic applications are based on the possibility of the versatile transformations of the unprotected B(OH)_2_ group *in situ* under the propargylboration conditions.

## Introduction

Allylation and propargylation of carbonyl compounds and imines is an attractive method for the highly selective synthesis of homoallylic and propargylic alcohols and amines.^1^ Development of new methodologies for synthesis of enantioenriched compounds with allyl and propargyl groups is particularly important, as these motifs often occur in natural products.^2^ Accordingly, a number of excellent methods have been reported for asymmetric propargylation.1c,^3^ The most recent trends in this field involve synthesis of sterically encumbered propargylic alcohols and imines occurring in natural products.2*a* Asymmetric catalysis for construction of acyclic small-molecules with adjacent quaternary carbon centers is one of the most challenging synthetic transformations.^4^ This structural motif is abundant in terpenoid natural products, such as in tertiary prenyl derivatives.2b,c Formation of a carbon–carbon single bond between sterically encumbered quaternary carbons is a particularly difficult synthetic problem. The strong steric repulsion between the bulky substituents (none of them is a hydrogen) leads to an elongated and very weak carbon–carbon σ-bond, which is difficult to create and easy to cleave.^5^

**Fig. 1:**
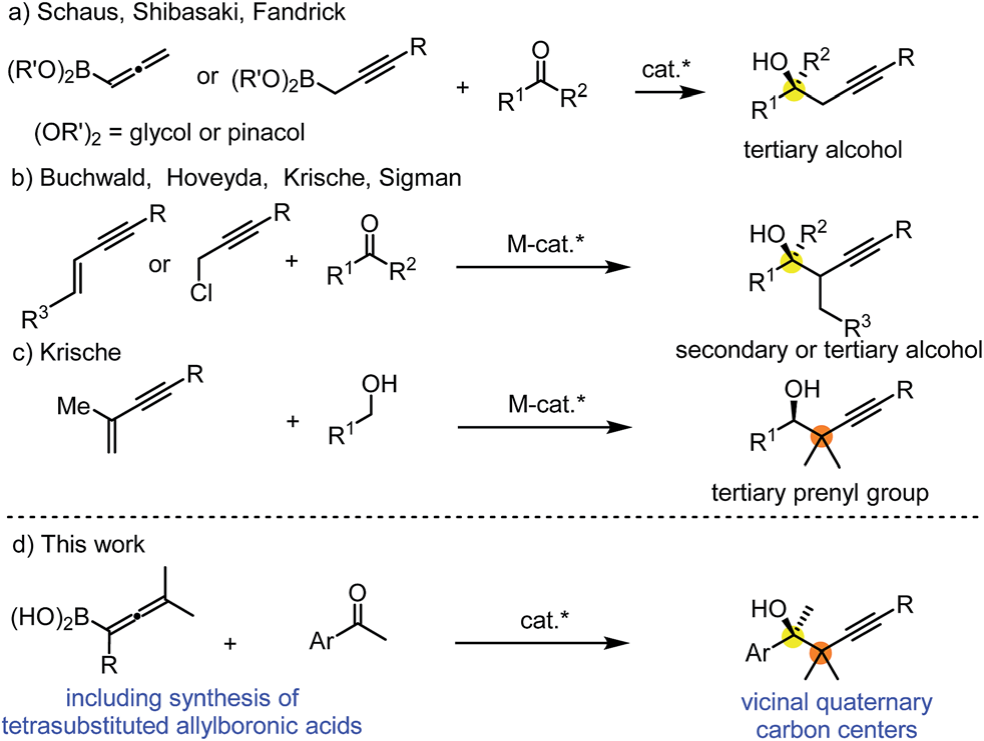
Asymmetric catalysis toward sterically encumbered homopropargylic alcohols.

Stereoselective propargylboration with allenylboron species became one of the most important transformations for creation of sterically crowded propargylic alcohols and imines.^6^ One of the important synthetic strategies for the formation of a tertiary stereocenter is based on the reaction of allenyl or propargylic boron reagents with ketones in the presence of a chiral catalyst (Fig. 1a). Schaus,6*b* Shibasaki6*a* and Fandrick6*v* reported these types of propargylboration reactions using mono- or disubstituted allenyl- and propargylboronates. Another very efficient method involves synthesis of secondary and tertiary propargylic alcohols without application of organometallic reagents using asymmetric metal catalysis (Fig. 1b).^7^ Krische reported an efficient method for *tert*-prenylation for terpenoid construction (Fig. 1c).6*w* By this method a propargylic all-carbon quaternary center could be created adjacent to a secondary alcohol. However, as far as we know, formation of vicinal quaternary carbon centers has never been reported for asymmetric propargylation reactions. In this paper we report our results for achievement of this goal *via* asymmetric propargylboration of ketones with tetrasubstituted allenylboronic acids (Fig. 1d).

## Results and discussion

Relatively few methods are available for the synthesis of allenylboronates and their analogs. The parent (unsubstituted) allenylboronate and a few alkyl-substituted derivatives can be prepared *via* Grignard (or other allenyl- or propargyl-metal mediated) reactions.6e,p,q,^8^ More recently, Cu- and Pd-catalyzed methods have been reported by the group of Ito/Sawamura and subsequently our and other groups for the synthesis of densely (tri- and tetra-) substituted allenyl-Bpin (Bpin = pinacolborane) derivatives.^9^

**Fig. 2:**
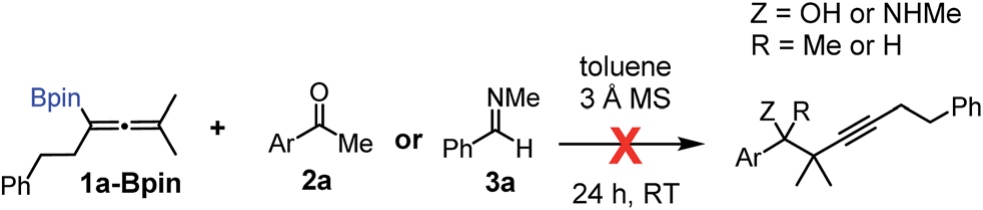
Pinacol protected allenylboronic acids are reluctant to react with ketones and imines.

However, densely functionalized, easily accessible tetrasubstituted allenyl-Bpin compounds have a relatively low reactivity profile. These compounds, such as **1a-Bpin**, react directly (without additives) with aldehydes6k,9a,b but according to our studies they are completely unreactive (Fig. 2) with ketones (**2a**) and imines (**3a**) at ambient temperature. Thus, homopropargyl alcohols and amines with adjacent quaternary carbons cannot be accessed using allenyl-Bpin reagents under mild conditions, which is required for a highly selective carbon–carbon bond formation.

The structural analogy between allyl-10 and allenylboron species suggests^11^ that allenylboronic acids are expected to be much more reactive than allenyl-Bpin (or other diol protected boron) reagents utilized to access crowded homopropargyl alcohols and amines. However, the lack of efficient methodologies for the preparation of densely functionalized allenylboronic acids is a fundamental problem for the implementation of this concept. Therefore, we undertook development of the first transition metal catalyzed synthesis of tri- and tetra-substituted allenylboronic acids, and subsequently we have exploited the synthetically useful properties of the unprotected B(OH)_2_ group for synthesis of enantiomerically enriched propargylic alcohols with vicinal quaternary carbons.

### Synthesis of allenylboronic acids

Copper-catalyzed transformation of propargylic carbonates under basic conditions was used for the borylation process.9a–c Similar to the catalytic synthesis of allylboronic acids, we employed diboronic acid^12^ (**5a**) as the B(OH)_2_ source (Table 1).10*a* Application of the acid form **5a** instead of B_2_pin_2_ (ref. 9a–c) (**5b**) under basic reaction conditions for synthesis of **1** required three important synthetic innovations: (1) the catalyst (CuOMe) was generated, *in situ* from mesitylcopper(i) (MesCu) and MeOH; (2) ethylene glycol was used for *in situ* protection of the boronic acid functionality in the product;^13^ and (3) we used weakly coordinating and easily removable P(OMe)_3_ as the ligand.

**Table 1:** Variation of reaction conditions for the synthesis of allenylboronic acid **1a**^*a*^

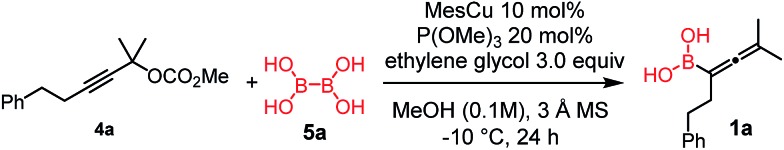		
Entry	Conditions	Yield^*b*^ [%]
1	No change	76
2	CuCl^*c*^ and KOMe^*d*^ instead of MesCu	52
3	CuCl^*c*^ and NaOMe^*d*^ instead of MesCu	49
4	CuCl^*c*^ and LiOMe^*d*^ instead of MesCu	65
5	Cul^*c*^ and LiOMe^*d*^ instead of MesCu	29
6	PPh_3_ instead of P(OMe)_3_	46
7	PCy_3_ or P(O-iPr)_3_ instead of P(OMe)_3_	0^*e*^
8	1,3-Propanediol instead of ethylene glycol	49
9	Without ethylene glycol	66
10	Without 3 Å MS	75
11	At 0 °C	16^*e*^
12	THF or toluene instead of MeOH	0

^*a*^General procedure: **4a** (0.10 mmol), **5** (0.15 mmol), mesitylcopper(i) (0.01 mmol), P(OMe)_3_ (0.02 mmol), ethylene glycol (0.30 mmol), and 3 Å MS were stirred in MeOH (1 mL) at –10 °C for 24 h.

^*b*^ ^1^H NMR-yields.

^*c*^10 mol%.

^*d*^20 mol%.

^*e*^Protodeborylation occurs.

**Fig. 3:**
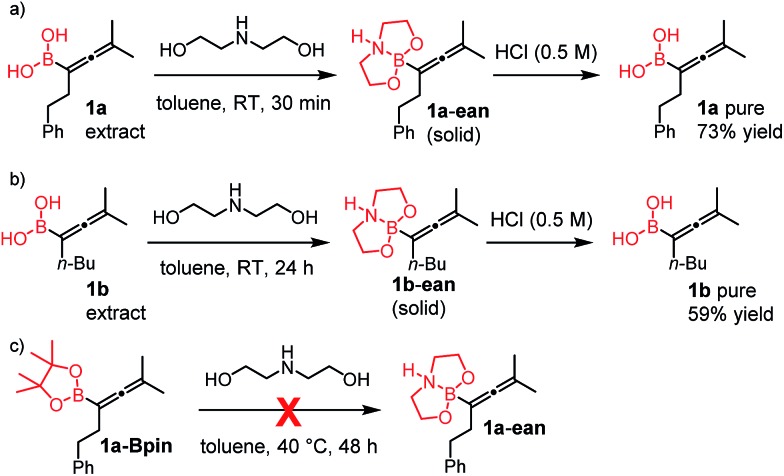
(a) and (b) Purification of allenylboronic acids **1a** and **1b**. (c) Attempted transformation of the pinacolate analogue **1a-Bpin** with diethanolamine.

Using the optimized conditions, allenylboronic acid **1a** could be obtained with 76% NMR yield (Table 1, entry 1). When the Cu-catalyst was generated from CuCl and alkali methoxides instead of MesCu/MeOH, the yields decreased to 49–65% (entries 2–4). Using CuI instead of CuCl led to a sharp decrease in the yield to 29% (entry 5). Phosphite P(OMe)_3_ could be replaced by PPh_3_, albeit in a diminished yield of 46% (entry 6). Application of bulky phosphorus based ligands, such as PCy_3_ and P(O-iPr)_3_, led to 0% yield of **1a** (entry 7). In these reactions, large amounts of protodeborylated allene formed, which indicates that **1a** is probably generated but likely undergoes a Cu-catalyzed protodeborylation. In the presence of ethylene glycol (*cf.* entries 9 and 1), the yield increased and only traces of protodeborylation products were observed. We found that in this *in situ* protection step, ethylene glycol is more efficient than its homolog, 1,3-propanediol (*cf.* entries 1 with 8–9). The addition of molecular sieves had a relatively weak effect on the yield (entry 10). When the reaction was conducted at 0 °C instead of –10 °C a large amount of protodeborylated product formed and the yield dropped substantially. Changing the solvent from methanol to toluene or THF prevented the formation of **1a** (entry 12). Allenylboronic acid **1a** is oxygen sensitive and resistant to crystallization (similar to analogs **1b–j**). Therefore, the purification was done by quenching the reaction mixture with 0.5 M HCl solution (to remove the glycol protecting group) and subsequent toluene extraction of the allenylboronic acid product. The resultant toluene solution of **1a** (and **1b–j**) can be stored under Ar (at –18 °C for several weeks) and used for all synthetic applications presented below (Tables 3–5). Compound **1a** can easily be converted to allenylboronates by adding the corresponding alcohols. For example **1a** and pinacol readily give **1a-Bpin** (see ESI† page 6). Reaction of **1a** with diethanolamine leads to **1a-ean** (Fig. 3). Easy formation of **1a-ean** can be exploited for further purification of **1a** by implementation of a methodology reported by Santos and co-workers (Fig. 3).^14^

This method is based on reaction of the (extracted) toluene solution of **1a** with diethanolamine (Fig. 3a). The esterification of the B(OH)_2_ group was very fast12*d* and the diethanolamine ester of **1a** (**1a-ean**) is precipitated from toluene. Allenylboronate **1a-ean** was an air- and moisture stable solid, which could be stored for several months. We attempted purification of **1a-ean** by silica gel chromatography but this purification method led to decomposition of **1a-ean**. Thus, after washing of **1a-ean** with ether, degassed toluene and 0.5 M HCl solution were added and the pure **1a** was extracted to the toluene phase. The purification process includes a slight loss of **1a** (yield 73%).

**Fig. 4:**
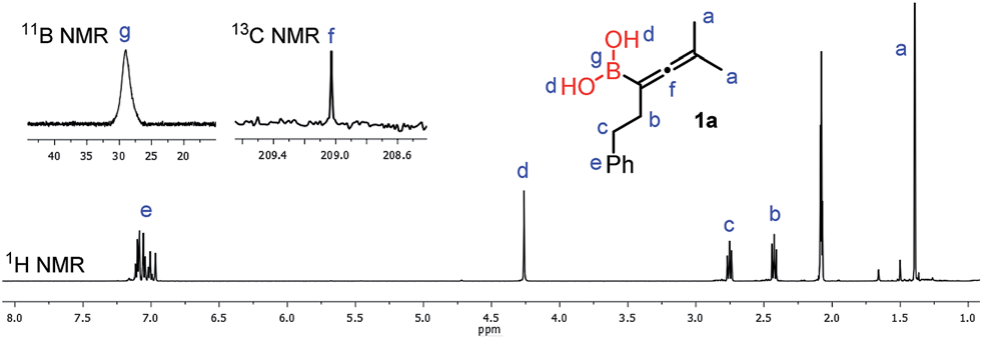
NMR spectra of allenylboronic acid **1a** with the characteristic B(OH)_2_ peak “d”.

The ^1^H-NMR spectrum (in toluene-*d*_8_) of purified **1a** (Fig. 4) clearly shows a peak (“d”) at 4.27 ppm, which belongs to the unprotected B(OH)_2_ group. From this spectrum it appears that the sample does not contain any glycol or other esters of the B(OH)_2_ group. The sample obtained by toluene extraction of the aqueous reaction mixture of the borylation is very similar (see the ESI†) to the purified one.

We have explored the synthetic scope of the above borylation reaction (Table 2) using various propargylic carbonates (**4b–h**, **4j**) and **5a** under the above (Table 1) optimized conditions. Compound **4b**, a close analog of **4a** reacted with excellent yield (94%) affording allenylboronic acid **1b** (entry 1). The butyl substituent can be replaced with more (**4c**) or less (**4d**) sterically demanding groups to provide the corresponding products in 61–67% yield (entries 2–3). The substituent can be varied at the propargylic carbon as well. Compound **4e** gives **1e** with 59% yield and substrates with two different propargylic substituents (**4f-g**) also react readily (entries 5 and 6) providing racemic allenylboronic acids **1f-g**. The borylation reaction also tolerates various types of substituents. In **4h** only the propargylic carbonate is transformed, while the aliphatic carboxylate remains unchanged (entry 7). We succeeded in performing the borylation of propargyl cyclopropane **4i** (entry 8). In this reaction, the cyclopropane ring opens instead of displacement of the carbonate leaving group. Even secondary propargylic carbonates (**4j**) could be borylated, albeit with a lower yield (34%) than their tertiary analogs (entry 9). The presented borylation method was scaled up thirty-fold with some drop of the yield to 62% (entry 1). Compound **1b** could also be further purified by the Santos method (Fig. 3b), as **1b-ean** precipitated as a solid. However, diethanolamine esters of the other allenylboronic acids (**1c–j**) were not precipitated from the toluene solution, and therefore these compounds cannot be purified further by this method.

**Table 2:** Synthesis of various allenylboronic acids^*a*^

Entry	Substrate	Product	Yield^*b*^ [%]
1	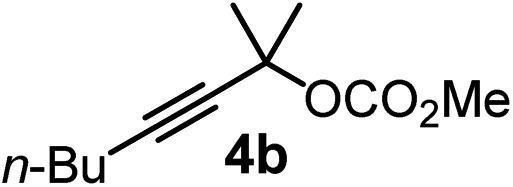	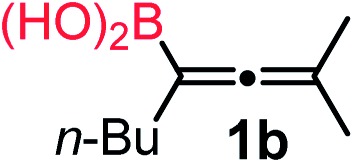	94
			62^*c*^
2	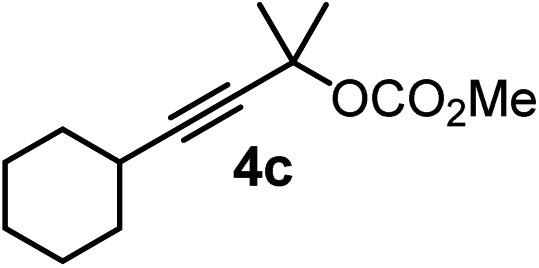	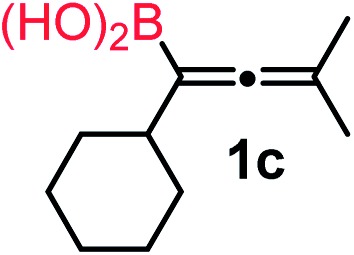	67
3	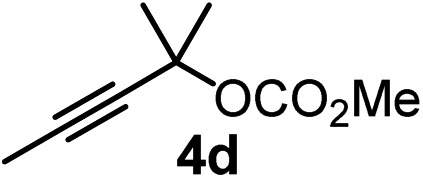	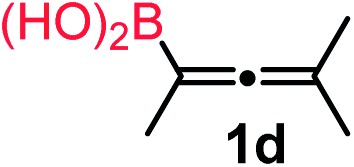	61
4	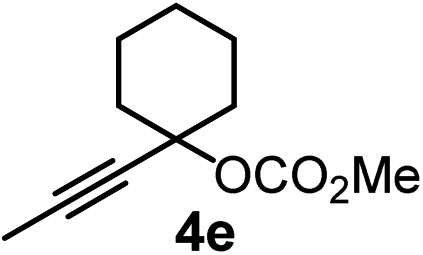	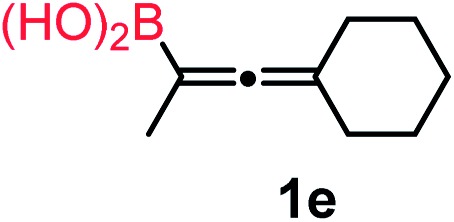	59
5	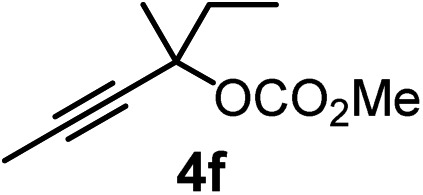	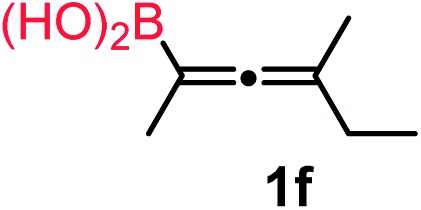	80
6	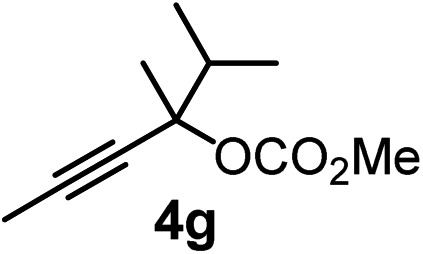	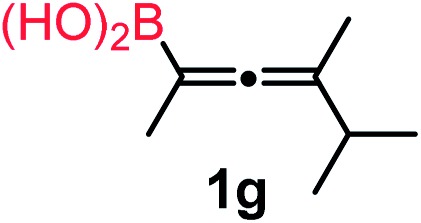	63
7	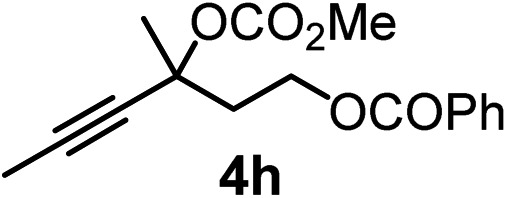	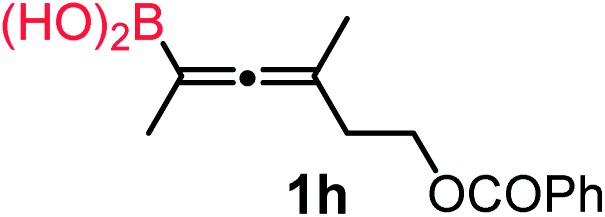	83
8	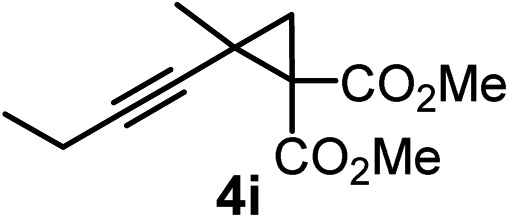	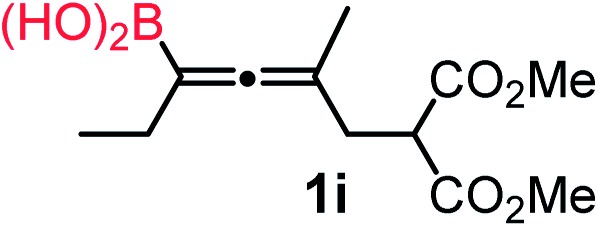	59
9	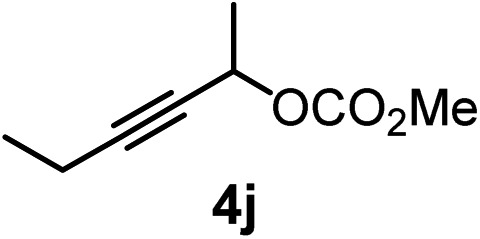	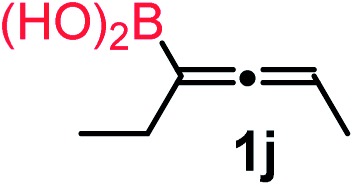	34

^*a*^General procedure: **4a** (0.10 mmol), **5** (0.15 mmol), mesitylcopper(i) (0.01 mmol), P(OMe)_3_ (0.02 mmol), ethylene glycol (0.30 mmol), and 3 Å MS were stirred in MeOH (1 mL) at –10 °C for 24 h.

^*b*^ ^1^H NMR-yield.

^*c*^Yield at 3 mmol scale.

### Extension of the borylation to synthesis of other allenyl boronates

**Fig. 5:**
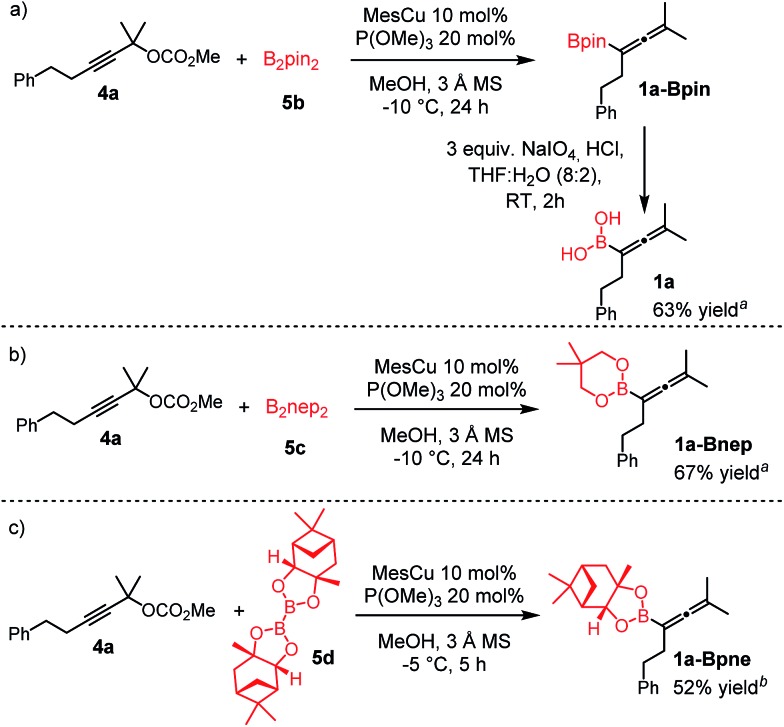
Extension of the methodology for synthesis of allenyl boronates. ^a1^H NMR yield. ^b^Isolated yield.

To our delight, the above described procedure for synthesis of allenylboronic acids **1a–j** was suitable for synthesis of other allenylboronates by replacement of the diboron reagent from diboronic acid **5a** to other species, such as B_2_pin_2_ (**5b**), neopentyl ester **5c** or chiral pinane ester **5d** (Fig. 5). The first method (Fig. 5a) is a useful complement to the previously reported copper and palladium catalyzed methods for synthesis of allenyl-Bpin compounds,9a–c such as **1a-Bpin**. Compound **1a-Bnep** is less stable than **1a-Bpin**, for example it decomposes under silica gel chromatography (Fig. 5b). However, it is air- and moisture stable, *i.e.* it is easier to handle than its unprotected counterpart **1a**. Compound **1a-Bpne** is a stable easily accessible chiral allenylboronate that can be purified by silica gel chromatography (Fig. 5c) and it can be stored in a refrigerator for several weeks. Similar to **1a-Bpin** (Fig. 2) **1a-Bpne** was unreactive toward ketones (such as **2a** and **2c**) at ambient temperature in 24 hours.

### Attempts for preparation of allenylboronic acids from allenyl boronates

Of course, the most efficient route to unprotected allenylboronic acids is using the above synthesis with application of unprotected diboronic acid **5a** (Tables 1 and 2). However, in order to extend the scope of the synthesis of allenylboronic acids, we studied the possibilities of hydrolysis of protected allenyl-Bpin compounds, such as **1a-Bpin**. The Santos method^14^ can be used for deprotection of various alkyl-Bpin derivatives, even aryl-Bpin compounds affording the corresponding organoboronic acids. However, the reaction of **1a-Bpin** with diethanolamine did not result in any formation of **1a-ean** at 40 °C in 48 h (Fig. 3c), and thus this method cannot be used for accessing allenylboronic acids from pinacol esters.

We tested another method based on oxidative hydrolysis of the pinacolborane functionality in the presence of NaIO_4_ reported by Falck and co-workers.^15^ This method was also used by Petasis and co-workers6*u* for oxidative hydrolysis of mono- and disubstituted allenyl-Bpin compounds. The oxidative hydrolysis of **1a-Bpin** was successful. Using this method after the borylation procedure, we were able to obtain **1a** with 63% overall yield (Fig. 5a). This yield was somewhat lower than the analog process using B_2_(OH)_4_ (**5a**) as the boron source (76%) but it is still viable for obtaining **1a**. We note that the level of purity of **1a** obtained by this multi-step procedure (Fig. 5a) is lower than by using **5a** as the boronate source (Table 1, entry 1) because of the use of more chemicals (*e.g.* 3 equiv. NaIO_4_) and pinacol as the protecting group.

### Propargylboration of ketones and imines with allenylboronic acids

Subsequently, we have studied the reactivity of these allenylboronic acids (Table 3). Compound **1b** reacted rapidly with aldehyde **2b** in toluene in the presence of molecular sieves without any further additives. The reaction was complete in 10 minutes at room temperature affording **6b** in 87% yield (entry 1). Under the same conditions (in 10 min) its Bpin analogue (**1b-Bpin**) did not provide any **6b** (entry 2) demonstrating that under identical reaction conditions **1b** is much more reactive than **1b-Bpin**. As mentioned above (Fig. 2) a densely functionalized allenyl-Bpin compound **1a-Bpin** did not react with ketones and imines in 24 h at room temperature. In contrast, ketones (such as **2a**) and aldimines **3a-b** reacted readily under these reaction conditions affording the corresponding homopropargylic alcohol (**6a**) and amine (**7a-b**) products in high yields (entries 3–5) confirming the superior reactivity of allenylboronic acids over their allenyl-Bpin counterparts. Dihydroisoquinoline **3c** also reacted smoothly affording homopropargyl derivative **7c** in 96% yield (entry 6). Even indole1*h***3d** underwent homopropargylation with **1a** at the C2 position affording **7d** in 82% yield (entry 7). Finally, we were able to react **1e** with imine **3b** affording compound **7e**. Remarkable features of the above reactions are the facile formation of one (entries 1, 4–8) or two (entry 3) quaternary centers connecting the newly formed C–C bond. The very high reactivity of an unprotected allenylboronic acid is the consequence of its ability to form the highly reactive boroxine (with the aid of molecular sieves), which is the anhydride of the boronic acid.^16^ Our DFT studies have shown that imines react with a much lower activation energy with allylboroxines than with allylboronic acids.10c,^16^ The reason is that the boroxines are much stronger Lewis acids than the corresponding boronic acids (or boronates).^16^

**Table 3:** Propargylation using allenylboronates^*a*^

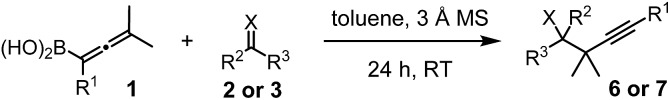				
Entry	Boronic acid	Substrate	Product	Yield^*b*^ [%]
1^*c*^	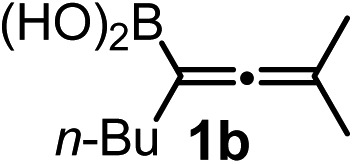	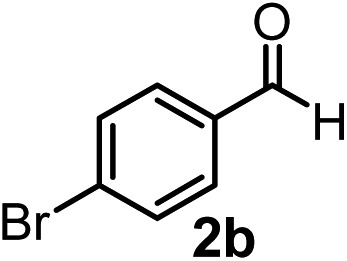	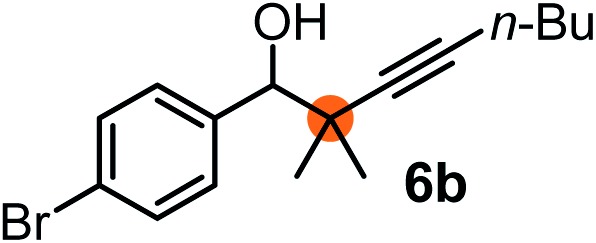	87
2^*c*^	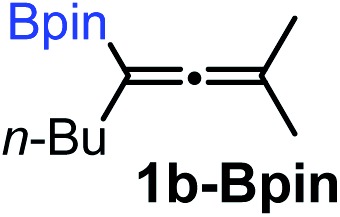	**2b**	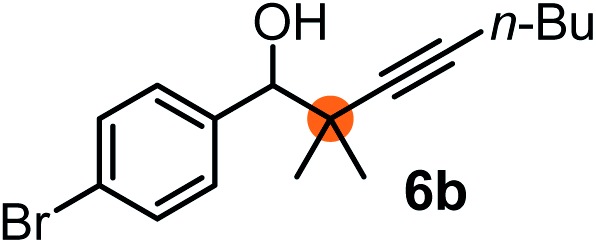	0
3	**1b**	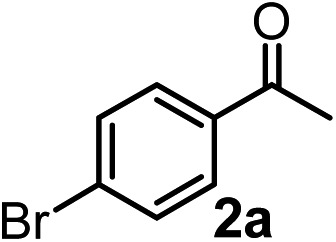	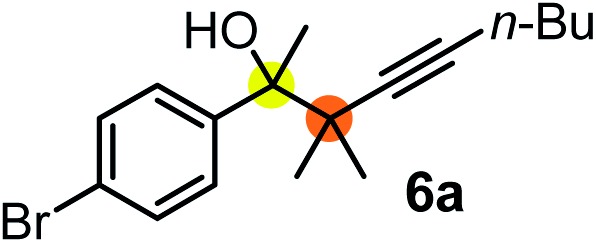	72
4	**1b**	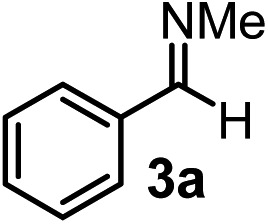	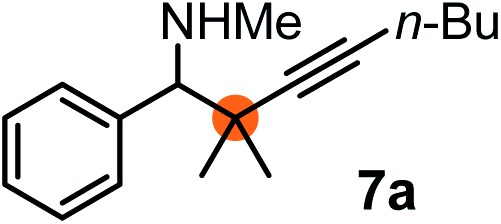	63
5	**1b**	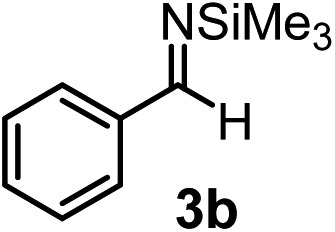	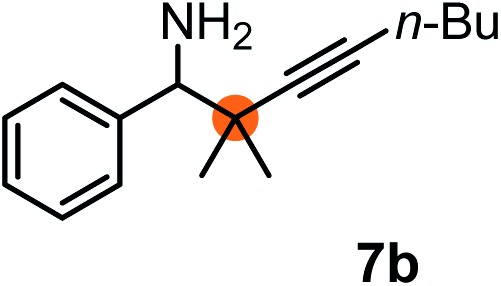	83
6	**1b**	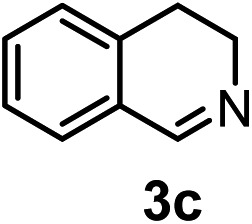	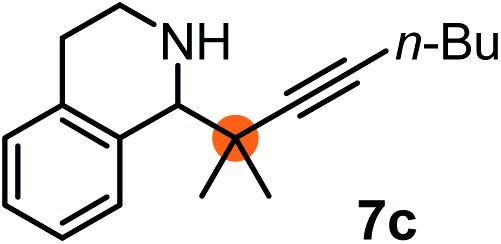	96
7	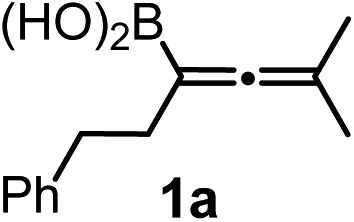	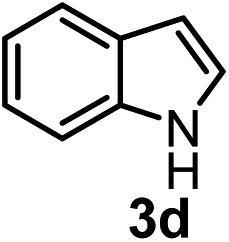	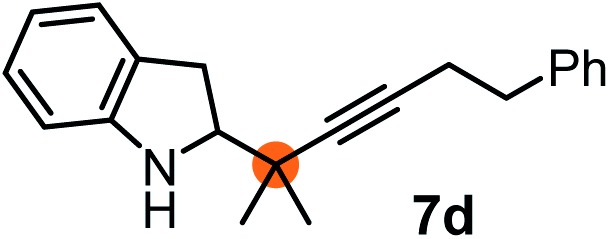	82
8	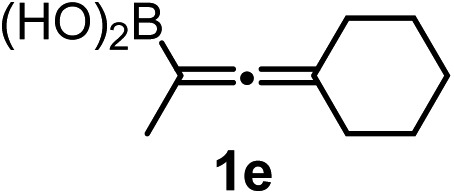	**3b**	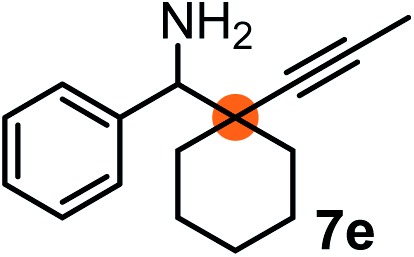	65

^*a*^Unless otherwise stated **2** or **3** (0.15 mmol) is dissolved in toluene with 3 Å MS. **1** (0.1 mmol) in toluene is added and stirred at RT for 24 h.

^*b*^Isolated yield.

^*c*^Reaction time was 10 minutes.

### Asymmetric propargylboration towards congested homopropargyl alcohols

Schaus and Barnett6*b* studied the reaction of the glycol ester of the parent and disubstituted allenylboronates with ketones. These authors found that in the presence of BINOL catalysts under the influence of microwave heating, useful levels of enantioselectivity could be achieved. We have found that tetra-substituted allenylboronic acid **1b** reacted readily with ketone **2a** at room temperature without the need for microwave heating (Table 4, entry 1). In the presence of catalytic amounts of (*S*)-dibromo-BINOL **8a** and EtOH, sterically encumbered homopropargylic alcohol **6a** with vicinal quaternary carbons was obtained with remarkably high enantioselectivity (94% ee) and in excellent yield (95%). Interestingly, the yield was higher in this reaction than in the racemic reaction (Table 3, entry 3) indicating that the reaction was accelerated in the presence of (*S*)-dibromo-BINOL **8a**. Application of an aliphatic alcohol, such as EtOH, in stoichiometric amount was essential to get a high enantioselectivity, in particular, when a catalytic amount of dibromo-BINOL **8a** was applied (entries 2 and 3). This observation is also in agreement with our previous experience with asymmetric allylation of ketones10*e* and imines10*f* and with the related DFT modeling study.^16^ When a stoichiometric amount of **8a** in the absence of EtOH was used, the ee dropped to 77% from 94% (Table 4, entry 2). When the amount of **8a** was reduced to 15 mol% and the reaction was conducted without any aliphatic alcohols the reaction proceeds with poor ee of 44%. The beneficial effects of addition of an aliphatic alcohol to increase the selectivity of the asymmetric allyl- and allenylboration by organoboronic acids are now a well-understood feature of these reactions (see the section below on the mechanism). However, the efficiency of a certain aliphatic alcohol on the improvement of the enantioselectivity depends on both the organoboronic acid and the employed electrophile. For example, for asymmetric allylboration of ketones,10*e* addition of ^*t*^BuOH had the most favourable effect on the enantioselectivity. However, when we replaced EtOH with ^*t*^BuOH in the asymmetric propargylboration reaction the ee dropped substantially from 94% to 55% (entry 4). Notably, finding the most efficient aliphatic alcohol improving the enantioselectivity of the allyl- and propargylboration reactions is inherently easier for allyl- and allenylboronic acids than their organoboronate counterparts. In organoboronates the B(OH)_2_ group is usually protected with an aliphatic alcohol (such as pinacol, glycol, isopropanol *etc.*), which may have detrimental effects on the enantioselectivity of the allyl- or propargylboration process. An additionally useful synthetic feature of the above asymmetric propargylboration reaction is that using (*R*)-dibromo-BINOL (the enantiomer of **8a**) as the catalyst the enantiomeric form of **6a** can be obtained with high ee and yield (entry 5).

**Table 4:** Variation of reaction conditions for the asymmetric propargylation of **2a**^*a*^

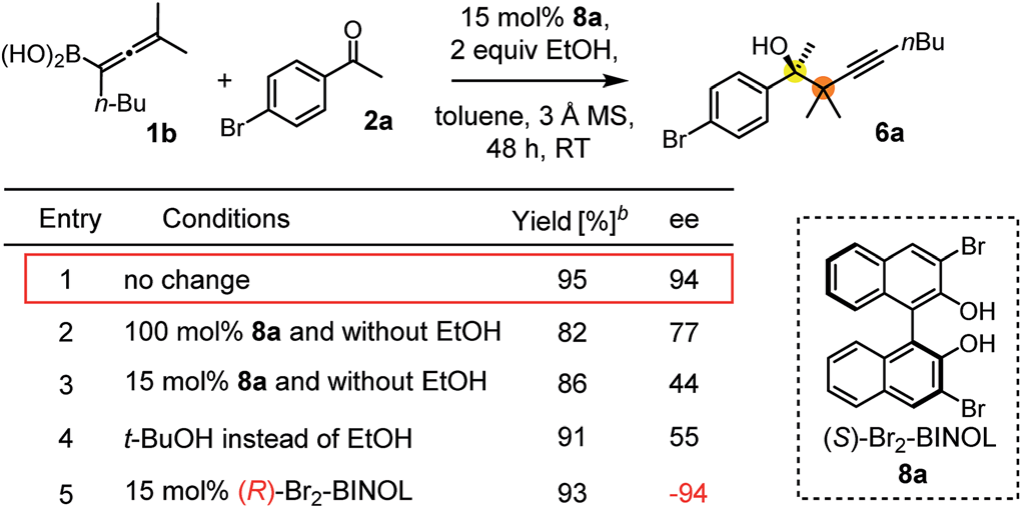

^*a*^EtOH or *t*-BuOH (0.2 mmol) and **8a** were added to **1b** (0.1 mmol) in toluene (0.2 M) with 3 Å MS, then 3 h later **2a** (0.15 mmol) was added and this mixture was stirred for 48 h at RT.

^*b*^Isolated yield.

We have briefly studied the synthetic scope of the above described asymmetric propargylation reactions for synthesis of encumbered homopropargylic alcohols with vicinal quaternary centers including a reversed prenyl motif (Table 5). Using the above described achiral allenylboronic acids **1a–e** and ketones **2c–g** the expected homopropargylic alcohols formed with 90–99% ee and 62–90% yields. The studied reactions involved the parent acetophenone **2c** (entry 1) and analogs with cyano **2d** (entry 2), acetate **2e** and bromo **2a** (entry 4) functionalities (entry 3). We made several derivatives (**6g–i**) containing a sulfone group in order to obtain crystalline products for determination of the absolute configuration of the products (entries 5–7) *via* X-ray diffraction. Unfortunately, all these products (**6g–i**) were oils resistant to crystallization. Finally, we succeeded in obtaining crystals of the ester of **6g** (ESI†), which were suitable for X-ray analysis. The absolute configuration of the stereogenic carbon in **6g-ester** was *R*, and thus we assigned all products arising from the (*S*)-dibromo-BINOL **8a** catalyzed reactions as the *R*-enantiomers. The reaction can be easily scaled up by five-fold without a significant change in yield or ee (entry 5).

**Table 5:** Asymmetric propargylation of various ketones.^*a*^ The vicinal quaternary carbons formed in the propargylboration process are colored

Entry		Substrates	Product	Yield^*b*^ [%]	ee
1	**1b**	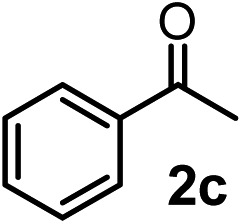	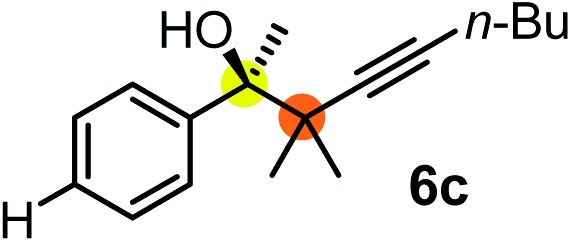	75	97
2	**1b**	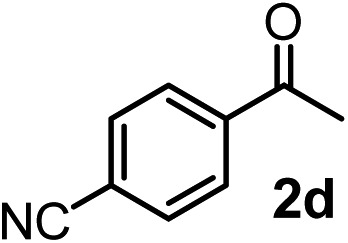	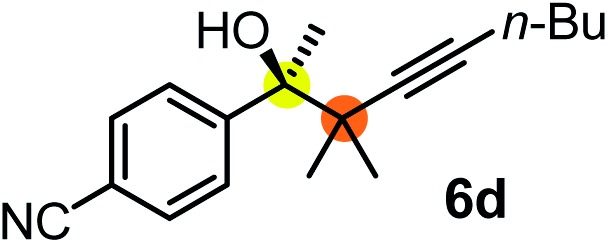	67	91
3	**1b**	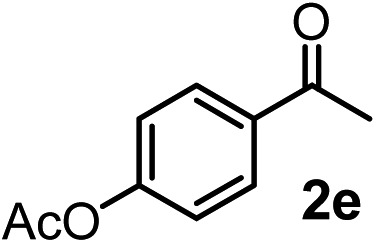	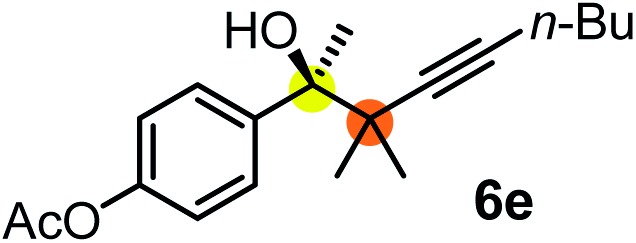	90	96
4^*c*^	**1c**	**2a**	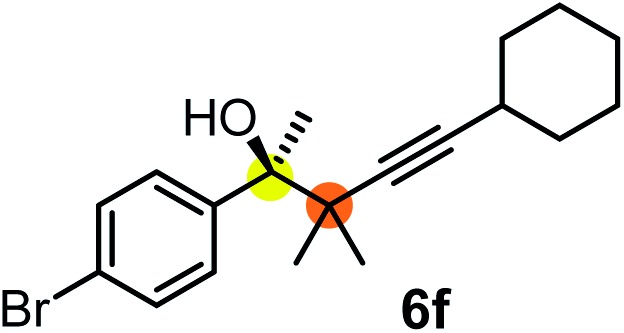	77	90
5^*d*^	**1b**	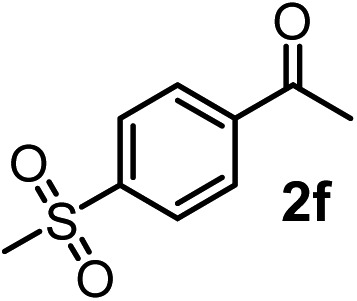	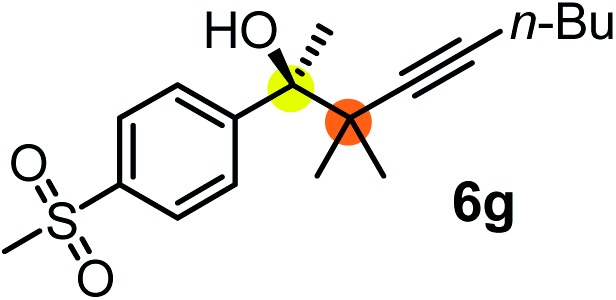	62 (70^*e*^)	94 (96^*e*^)
6^*c*^	**1a**	**2f**	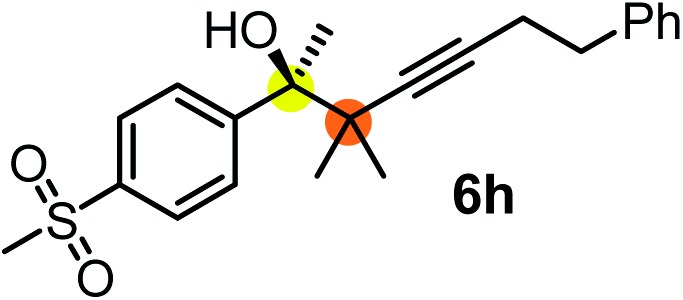	63	96
7^*c*^	**1e**	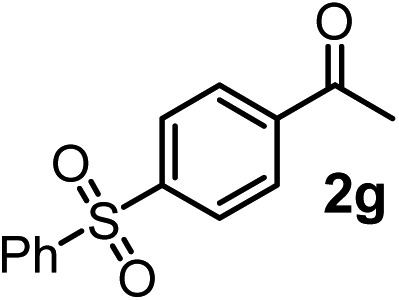	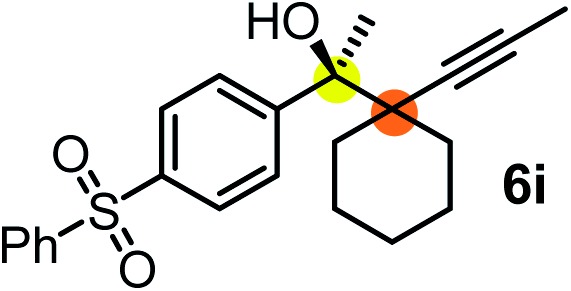	64	99

^*a*^EtOH (0.2 mmol) and **8a** were added to **1** (0.1 mmol) in toluene (0.2 M) with 3 Å MS, then 3 h later **2** (0.15 mmol) was added and this mixture was stirred for 48 h at RT.

^*b*^Isolated yields.

^*c*^Reaction time 72 h.

^*d*^Reaction time 90 h. Conc. was 0.1 M. 20 mol% **8a**.

^*e*^0.5 mmol scale, using 30 mol% **8a** and the reaction time was 90 h.

**Fig. 6:**
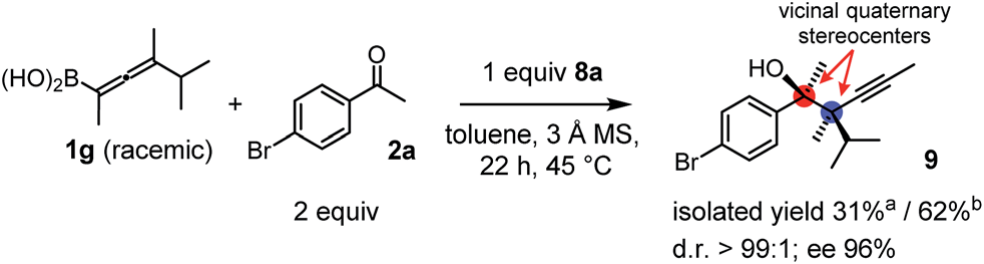
Kinetic resolution of **1g** affording a single enantiomeric product with adjacent quaternary stereocenters. ^a^Yield is based on racemic **1g**. ^b^Yield is based on the reactive enantiomer of **1g**.

Racemic allenylboronic acids are not supposed to react with high selectivity in conventional asymmetric catalysis. However, Schaus and co-workers6*b* have shown that disubstituted (racemic) allylboronates undergo kinetic resolution with benzophenone (**2c**) in the presence of **8a**. We have also found that racemic **1g** reacted with ketone **2a** and a stoichiometric amount of **8a**, affording **9** in very high enantio- (96% ee) and diastereoselectivity (Fig. 6). The reaction of **1g** with **2a** was slower than with the dimethyl substituted achiral allenylboronic acids most probably because the steric congestion was further increased by the presence of an isopropyl group. Therefore, the reaction temperature was increased to 45 °C, and 100 mol% of BINOL had to be employed for full conversion of ketone **2a**. Interestingly, in the absence of BINOL **8a** boronic acid **1g** did not react with ketone **2a** under the otherwise identical conditions. This is another indication that the reactivity of allenylboronic acid is increased in the presence of BINOL **8a** (Fig. 6).

As far as we know compound **9** is the first reported enantioenriched homopropargylic compound, which was synthesized by asymmetric catalysis forming adjacent quaternary stereogenic centers. Compound **9** was obtained as an oil, which resisted functionalization. The tertiary alcohol center was assigned on the basis of the X-ray structure of **6g-ester**. The relative configuration was tentatively assigned as syn based on the similarities of the spectral data and optical rotation reported by Schaus for a related product.6*b*

### Suggested mechanism

**Fig. 7:**
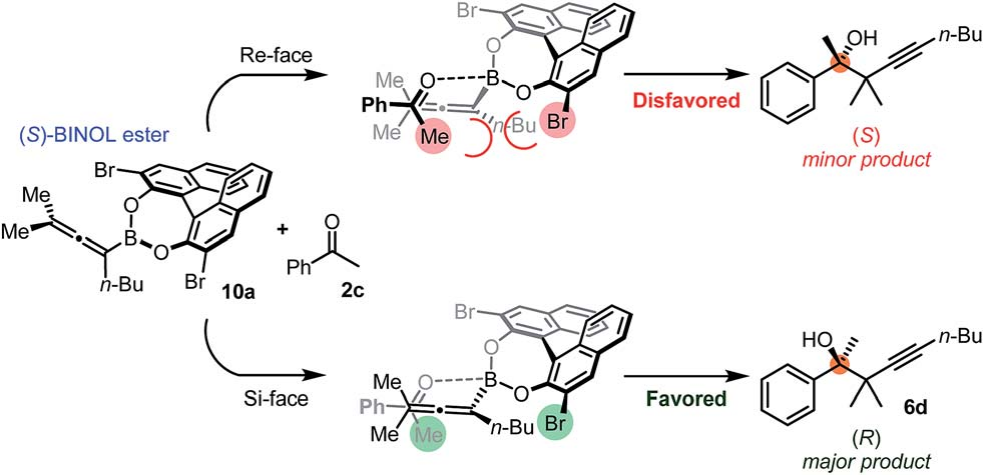
Plausible mechanism of the stereoselection exemplified with the propargylation of **2c**.

The suggested mechanism for the stereoselection, exemplified by the reaction of **1b** with **2c** in the presence of (*S*)-dibromo-BINOL **8a** (Table 5, entry 1), is shown in Fig. 7. We assume that BINOL **8a** esterifies the B(OH)_2_ group of **1b** to give chiral allenylboronate **10a** and the reaction proceeds *via* a Zimmerman-Traxler (Z.-T.) TS (Fig. 7).6d,e,k The assumption of the diesterification of **1b** with **8a** is based on our^16^ and others'^17^ DFT studies on asymmetric allylboration of ketones. These studies show that diesters of BINOL, such as **10a**, react with a much lower activation barrier with ketones than mono-esters or esters of aliphatic alcohols. In the case of the Re-face arrangement the Me group of ketone **2c** clashes with one of the bromo substituents of the BINOL moiety, therefore this TS is disfavoured. However, in the Si-face TS this steric repulsion does not occur, and therefore, formation of the *R*-product is favoured (Fig. 7).

**Fig. 8:**
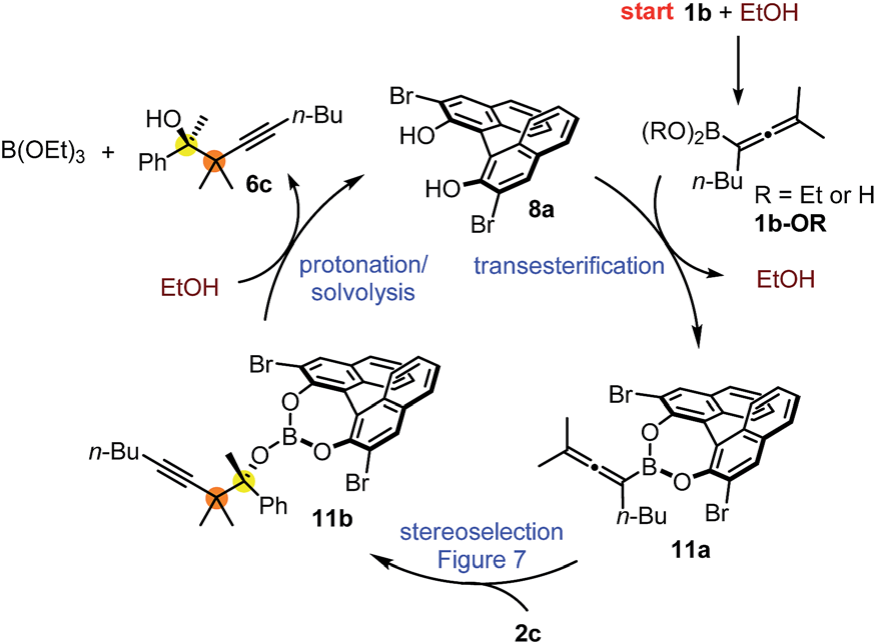
Proposed mechanism for the asymmetric propargylation exemplified with the reaction of **1b** with **2c**.

The proposed catalytic cycle for the propargylboration is given in Fig. 8. Accordingly, the enantioselective version of the reaction starts with mono- or diesterification of allenyl boronic acid **1b** with EtOH affording **1b-OR**. This esterification process is very fast and it can be observed by ^1^H NMR spectroscopy (see ESI† page 27). As mentioned above,^16^ boronic esters of aliphatic alcohols (such as EtOH) react much slower (if at all) than allylboronic acids/boroxines with ketones.10b,^11^ Thus **1b-OR** does not react directly with ketone **2c**, effectively shutting down the racemic background reaction (Table 3, entry 3). Therefore, when the reaction was performed without EtOH (Table 4, entry 3), the yield remained high but the ee dropped considerably indicating that considerable amount of racemic product was formed by the reaction of **1b** or its boroxine and **2a**. Compound **1b-OR** may undergo transesterification with BINOL **8a** forming a highly reactive chiral allenylboronate **11a**. The transesterification of aliphatic esters (such as ethyl-ester) of **1b** is probably much faster than for pinacol ester. The difficult transesterification of **1a-Bpin** with diethanolamine is mentioned above (Fig. 3c). The very high reactivity of BINOL esterified allylboronic acids towards electrophiles was demonstrated by our recent DFT studies.^16^ The high reactivity of **11a** is due to the presence of phenolic oxygen atoms, which conjugate less efficiently with the empty B(p_π_) orbital than the oxygen atoms of aliphatic alcohols (*e.g.* EtOH). Therefore, the B(p_π_) orbital of **11a** will be an efficient electron acceptor for the O(n_π_) orbital of ketone **2c** in the cyclic TS (Fig. 7) of the reaction. The stereoselectivity of the reaction is determined in the **11a** + **2c** → **11b** process (Fig. 7). Formation of **11b** involves trapping of the BINOL catalyst. The added EtOH probably mediates decomposition of **11b**, effectively releasing the BINOL catalyst **8a** back into the catalytic cycle. Thus, the uncatalyzed racemic propargylation can be suppressed by the “dual action” of EtOH (*i.e.***1b** → **1b-OR** and **11b** → **8a** processes) increasing the ee of the reaction.

## Conclusion

We have presented a new approach for the propargylboration of ketones and imines, which is suitable for synthesis of sterically encumbered homopropargylic alcohols and amines. The key reagent in this process is unprotected allenylboronic acid **1**. In this study, we report the development of the first transition metal catalysed procedure for the synthesis of allenylboronic acids. This process is based on the application of diboronic acid as the commercially available B(OH)_2_ source and utilizes easily accessible propargylic carbonates. The diboron reagent scope of the reaction is also broad. The newly developed process is suitable for synthesis of allenylboronates as well, including chiral ones. The broad application of allenylboronic acids with various electrophiles is based on the high reactivity of the unprotected B(OH)_2_ group. The addition of molecular sieves leads to the formation of allenylboroxine, which is highly reactive towards carbonyls and imines. Therefore, tetra-substituted allenylboronic acids readily react with aldehydes, ketones, imines, and indoles at room temperature. Convenient *in situ* esterification of the B(OH)_2_ group with the diboromo-BINOL/EtOH system allows for the catalytic asymmetric propargylboration of ketones at room temperature affording sterically encumbered homopropargyl alcohols with neighbouring quaternary carbons. In addition, kinetic resolution of racemic allenylboronic acid could be performed in the presence of (*S*)-dibromo-BINOL (**8a**) catalyst. This reaction is suitable for creation of vicinal quaternary stereocenters with high enantio- and diastereoselectivity. We hope that similar to allylboronic acids,10g–j access to allenylboronic acids will also inspire new synthetic applications towards complex organic molecules and natural products.^2^

## Conflicts of interest

There are no conflicts to declare.

## Supplementary Material

Supplementary information

Crystal structure data
