# Supplementary material for: Copper-catalyzed synthesis of allenylboronic acids. Access to sterically encumbered homopropargylic alcohols and amines by propargylboration
Source: Chem Sci. 2018 Feb 19;9(13):3305–12. doi: 10.1039/c7sc05123a (PMC5915797; doi:10.1039/c7sc05123a)

Electronic Supplementary Material (ESI) for Chemical Science.
This journal is © The Royal Society of Chemistry 2018

Supporting Information

Copper-Catalyzed Synthesis of Allenylboronic Acids. Access to Sterically Encumbered Homopropargylic Alcohols and Amines by Propargylboration with Allenylboronic Acids

Jian Zhao,[a†] Sybrand J. T. Jonker,[a†] Denise N. Meyer,[a] Göran Schulz,[a] C. Duc Tran,[a] Lars Eriksson[b] and Kálmán J. Szabó*[a]

[a]Department of Organic Chemistry, Stockholm University (Sweden), [b]Department of Materials and Environmental Chemistry, Stockholm University (Sweden).

E-mail: kalman@organ.su.se, web: http://www.organ.su.se/ks

Contents:

General Information 2
Experimental Procedures and Spectral Data 2
Esterification of boronic acid **1b** with EtOH 26
References 27
^{1}H, ^{13}C and ^{11}B spectra 28

General Information

All reactions were carried out in dried glassware under argon or in an argon-filled glove box. Allenyl boronate **1a-Bpin** and propargyl carbonates (**4a-j**) were synthesized according to previously reported literature procedures.[1-2] All other chemicals were obtained from commercial sources and used as received. Dry toluene, activated 3 Å molecular sieves and anhydrous methanol were stored in an argon-filled glove box. ^{1}H NMR and ^{13}C NMR spectra were recorded in $CDCl_3$ (internal standard: 7.26 ppm, ^{1}H; 77.16 ppm, ^{13}C), toluene-d_8 (internal standard: 2.08, 6.97, 7.01, 7.09 ppm, ^{1}H; 20.43, 125.13, 127.96, 128.87, 137.48 ppm, ^{13}C), C_6D_6 (internal standard: 7.16 ppm, ^{1}H; 128.06 ppm, ^{13}C) and CD_3CN (internal standard: 1.94 ppm, ^{1}H; 1.32, 118.26 ppm, ^{13}C) using 400 MHz and 500 MHz spectrometers. High resolution mass data (HRMS) were obtained using the ESI technique. For column chromatography, silica gel (35-70 microns) was used. TLC was performed on aluminium backed plates pre-coated (0.25 mm) with silica gel 60 F_{254} with a suitable solvent system and was visualized using UV fluorescence and/or developed with $KMnO_4$ or phosphomolybdic acid. Chiral SFC was performed using Chiralpak IA, Chiralpak IB and Chiralpak IC columns (4.6 × 250 mm × 5 µm) eluting with MeOH/CO_2 and monitored by DAD (Diode Array Detector). Retention times (R_t) are quoted in minutes. Optical rotation was measured on an AUTOPOL IV polarimeter.

Experimental Procedures and Spectral Data

Procedure A for the synthesis of allenylboronic acids

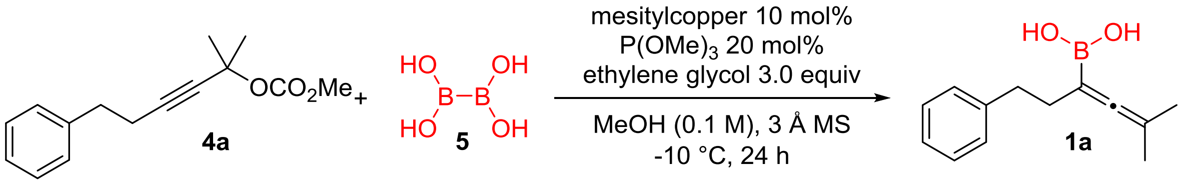

A vial was charged with mesitylcopper(I) (0.01 mmol, 1.8 mg), trimethyl phosphite (0.02 mmol, 2.4 µL), ethylene glycol (10 M solution in MeOH, 0.30 mmol, 30 µL), 3 Å molecular sieves, and MeOH (0.8 mL). The reaction mixture was stirred for 1 hour at room temperature. Then a MeOH solution (0.2 ml) of propargylic carbonate **4** (0.10 mmol), naphthalene (internal standard, 5.5 mg) and diboronic acid **5a** (1.0 M solution in MeOH, 0.15 mmol, 0.2 mL) was added dropwise to the reaction mixture via syringe at -10 °C. Subsequently, this reaction mixture was stirred at -10 °C for 24 hours. Then the reaction mixture was filtered through a 0.45 µm syringe PTFE filter and transferred to degassed HCl solution (0.5 M). The resulting slurry was extracted with degassed toluene (toluene-d_8 for ^{1}H NMR sample) and washed twice with a phosphate buffer-NaCl solution (prepared from 100 mL commercial phosphate buffer solution (pH = 7) by addition of 10 g NaCl). The yield of allenylboronic acids **1** was determined by ^{1}H NMR spectroscopy using naphthalene as an internal standard.

Procedure B for purification of allenylboronic acids. A modified method reported by Santos and co-workers[3] was used for further purification of the allenylboronic acids. A vial was charged with diethanolamine (0.12 mmol, 13 mg), and then, solution of allenylboronic acid in toluene (0.10 mmol, 0.5 ml, 0.2 M) was added dropwise via syringe at room temperature. After a few minutes a white precipitate formed and the reaction mixture was stirred until the starting material was completely consumed (30 min for **1a** and 24 h for **1b**). The precipitate was filtered, washed with ether (3 x 5 mL) and dried under vacuum. To a solution of this white precipitate in degassed toluene(-d_8), degassed HCl solution (0.5 M) was added. Then, the resulting mixture was shaken for 5 min and extracted with degassed toluene(-d_8) and washed with a phosphate buffer-NaCl solution.

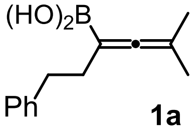

1a

(5-Methyl-1-phenylhexa-3,4-dien-3-yl)boronic acid (1a). This compound was prepared according to procedure A (yield 76%), and subsequently purified by procedure B (yield 73%). ^{1}H NMR (400 MHz, tol-d_8): δ 7.16-6.96 (m, 5H), 4.27 (s, 2H), 2.75 (t, *J* = 7.6 Hz, 2H), 2.42 (dd, *J* = 8.0, 6.8 Hz, 2H), 1.39 (s, 6H); ^{13}C NMR (100 MHz, tol-d_8): δ 209.4, 142.6, 128.9, 128.4, 125.8, 93.6, 36.0, 31.3, 19.5; ^{11}B NMR (128 MHz, tol-d_8): δ 29.

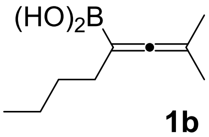

1b

(2-Methylocta-2,3-dien-4-yl)boronic acid (1b). This compound was prepared according to procedure A (yield 94%), and subsequently purified by procedure B (yield 59%). ^{1}H NMR (500 MHz, tol-d_8): δ 4.73 (s, 2H), 2.16 (t, *J* = 7.5 Hz, 2H), 1.53-1.47 (m, 2H), 1.50 (s, 6H), 1.37-1.30 (m, 2H), 0.90 (t, *J* = 7.4 Hz, 3H); ^{13}C NMR (125 MHz, tol-d_8): δ 209.5, 93.5, 32.5, 29.7, 23.1, 20.0, 14.6; ^{11}B NMR (128 MHz, tol-d_8): δ 29.

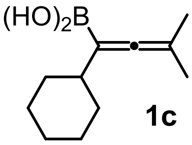

1c

(1-Cyclohexyl-3-methylbuta-1,2-dien-1-yl)boronic acid (1c). This compound was prepared according to procedure A (yield 67%). The ^{1}H NMR data was determined from the toluene solution of **1c** obtained after the final extraction in procedure A. ^{1}H NMR (500 MHz, tol-d_8): δ 4.32 (s, 2H), 2.19-2.13 (m, 1H), 1.51 (s, 6H), 1.36-1.09 (m, 10H).

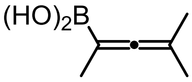

1d

(4-Methylpenta-2,3-dien-2-yl)boronic acid (1d). This compound was prepared according to procedure A (yield 61%). The ^{1}H NMR data was determined from the toluene solution of **1d** obtained after the final extraction in procedure A. ^{1}H NMR (500 MHz, tol-d_8): δ 4.18 (s, 2H), 1.76 (s, 3H), 1.46 (s, 6H).

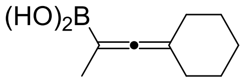

1e

(1-Cyclohexylideneprop-1-en-2-yl)boronic acid (1e). This compound was prepared according to procedure A (yield 59%). The ^{1}H NMR data was determined from the toluene solution of **1e** obtained after the final extraction in procedure A. ^{1}H NMR (500 MHz, tol-d_8): δ 4.10 (s, 2H), 1.79 (s, 3H), 1.46-1.38 (m, 4H), 1.34-1.24 (m, 6H).

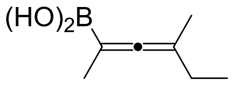

1f

(4-Methylhexa-2,3-dien-2-yl)boronic acid (1f). This compound was prepared according to procedure A (yield 80%). The ^{1}H NMR data was determined from the toluene solution of **1f** obtained after the final extraction in procedure A. ^{1}H NMR (500 MHz, tol-d_8): δ 4.27 (s, 2H), 1.78 (s, 3H), 1.73-1.65 (m, 2H), 1.48 (s, 3H), 0.87 (t, J = 7.3 Hz, 3H).

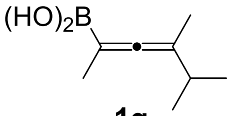

1g

(4,5-Dimethylhexa-2,3-dien-2-yl)boronic acid (1g). This compound was prepared according to procedure A (yield 63%). The ^{1}H NMR data was determined from the toluene solution of **1g** obtained after the final extraction in procedure A. ^{1}H NMR (500 MHz, tol-d_8): δ 4.13 (s, 2H), 1.97-1.87 (m, 1H), 1.80 (s, 3H), 1.53 (s, 3H), 0.95 (d, J = 6.6 Hz, 2H), 0.92 (d, J = 6.8 Hz, 2H).

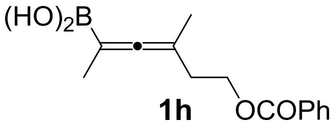

1h

(6-(Benzoyloxy)-4-methylhexa-2,3-dien-2-yl)boronic acid (1h). This compound was prepared according to procedure A (yield 83%). The ^{1}H NMR data was determined from the toluene solution of **1h** obtained after the final extraction in procedure A. ^{1}H NMR (500 MHz, tol-d_8): δ 8.12-8.05 (m, 5H), 4.69 (s, 2H), 4.33-4.23 (m, 2H), 4.17-4.11 (m, 2H), 1.75 (s, 3H), 1.47 (s, 3H).

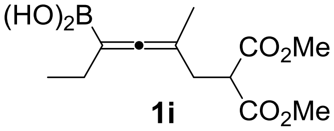

1i

(8-Methoxy-7-(methoxycarbonyl)-5-methyl-8-oxoocta-3,4-dien-3-yl)boronic acid (1i). This compound was prepared according to procedure A (yield 59%). The ^{1}H NMR data was determined from the toluene solution of **1i** obtained after the final extraction in procedure A.^{1}H NMR (500 MHz, tol-d_8): δ 4.81 (s, 2H), 3.52-3.48 (m, 1H), 3.34 (s, 3H), 3.28 (s, 3H), 2.53-2.43 (m, 2H), 2.17-2.11 (m, 2H), 1.47 (s, 3H), 1.03 (t, J = 7.4 Hz, 3H);

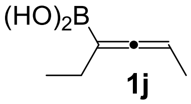

1j

Hexa-3,4-dien-3-ylboronic acid (1j). This compound was prepared according to procedure A (yield 34%). The ^{1}H NMR data was determined from the toluene solution of **1j** obtained after the final extraction in procedure A. ^{1}H NMR (500 MHz, tol-d_8): δ 4.04 (s, 2H), 1.91 (q, J = 7.5 Hz, 2H), 1.43 (d, J = 6.8 Hz, 3H), 1.09 (t, J = 7.4 Hz, 3H);

Comparision of the ^{1}H NMR spectra of 1a obtained after the toluene extraction in procedure A and after purification using procedure B. Spectrum i) below is the ^{1}H NMR spectrum of **1a** (in wet toluene-d_8, 500 MHz) purified by the above method. Peak "**d**" belongs to the unprotected B(O<u>H</u>)$_2$ group. The spectrum does not display any peaks arising from boronic esters or boroxine. Spectrum ii) shows the ^{1}H NMR spectrum of **1a** obtained by extraction of the reaction mixture as described in "Procedure A for the synthesis of allenylboronic acids" above. Comparision of the ^{1}H NMR spectrum of the purified (spectrum i) and extracted (spectrum ii) sample of **1a** reveals that the extracted sample is sufficiently pure and contains the B(OH)$_2$ form of **1a**. This also means that under the extractive purification procedure the glycol ester of B(OH)$_2$ is completely hydrolyzed. As mentioned in the main text, all the reactions (Table 3-4) were carried out with extracted allenylboronic acids, such as **1a** (spectrum ii).

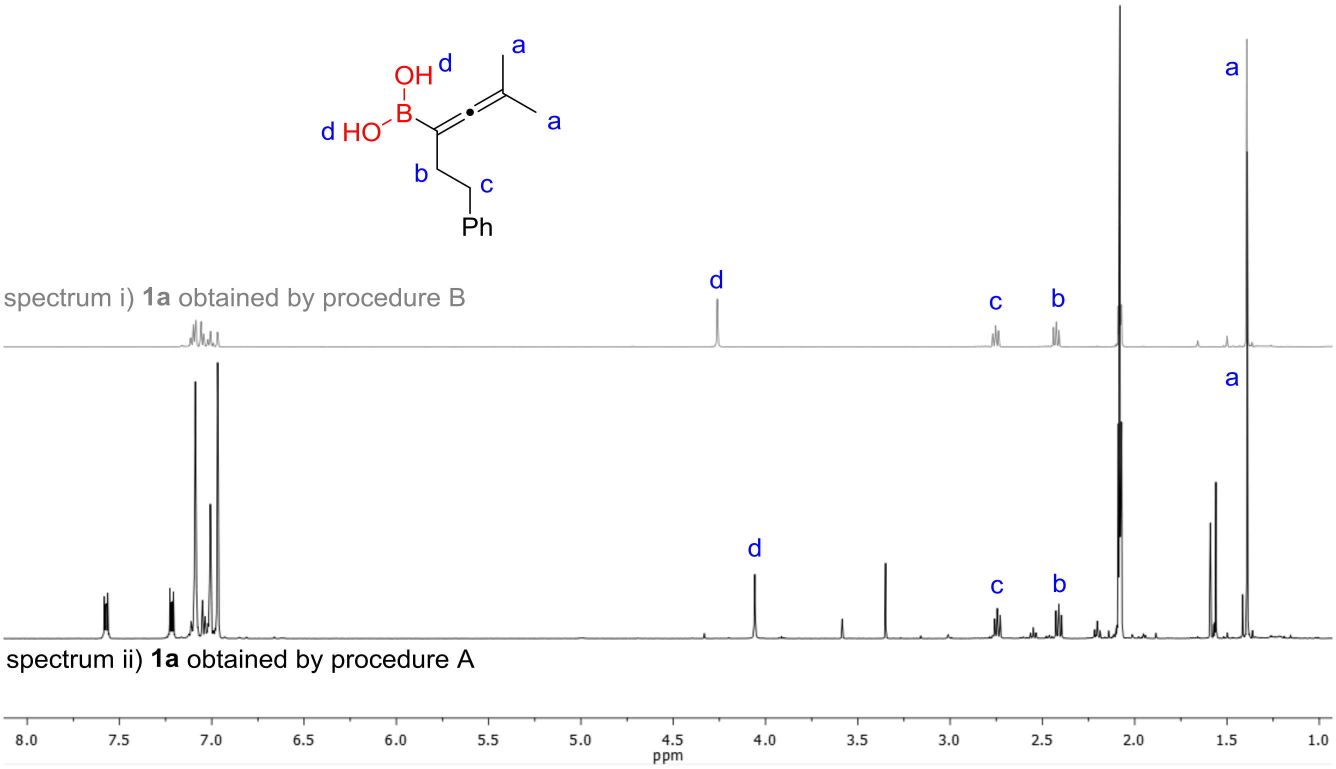

Conversion of boronic acid 1a to its pinacol ester 1a-Bpin

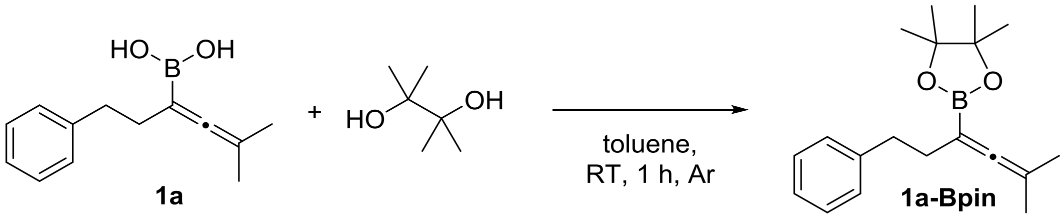

A vial was charged with pinacol (0.20 mmol, 24 mg) and 3 Å molecular sieves. Then, a toluene solution (obtained by procedure A) of allenylboronic acid (0.10 mmol, 0.5 ml, 0.19 M) was added dropwise via syringe at room temperature and stirred for 1 hour. Completion of the reaction was monitored by ^{1}H NMR. After a complete conversion of allenylboronic acid **1a**, the reaction mixture was diluted with diethyl ether (1.0 mL). The molecular sieves were filtered off through a short silica pad (about 1 cm in a pipette) using ethyl acetate/hexane (1:1) as an eluent. The solvent was removed and the residue was purified by a rapid silica gel chromatography using a mixture of pentane/ethyl acetate 30:1 to 20:1 (v/v) as eluent affording a colorless oil in 87% yield (26 mg, 0.09 mmol). NMR data for the pinacol ester of **1a**. ^{1}H NMR (400 MHz, C_6D_6): δ 7.18-1.02 (m, 5H), 2.92 (t, J = 7.2 Hz, 2H), 2.66 (t, J = 7.2 Hz, 2H), 1.55 (s, 6H), 1.05 (s, 12H); ^{13}C NMR (100 MHz, C_6D_6): δ 212.3, 143.2, 129.5, 128.9, 126.3, 91.9, 83.7, 36.8, 33.2, 25.3, 20.3. ^{11}B NMR (128 MHz, C_6D_6): δ 31. The spectral data is in agreement with the literature values.[4]

Attempted transesterification of 1a-Bpin with diethanolamine. A vial was charged with diethanolamine (0.09 mmol, 9.3 mg), **1a-Bpin** (0.08 mmol, 24 mg) and toluene (0.5 mL). This mixture was heated to 40 °C under continuous stirring and monitored by ^{1}H NMR. No precipitation was observed and the mixture remained transparent. After 48 hours of stirring, ^{1}H NMR analysis of the mixture showed only **1a-Bpin** and no traces of **1a-ean** were observed. This experiment shows that **1a-Bpin** cannot be converted to **1a** via transesterification with diethanolamine.

Synthesis of allenylboronic acid 1b on 3 mmol scale. A Schlenk tube was charged with mesitylcopper(I) (55 mg, 0.30 mmol), 3 Å molecular sieves, and trimethylphosphite (71 µL, 0.60 mmol). To this reaction mixture MeOH (15 mL) were added, followed by ethylene glycol (0.9 mL, 10 M in MeOH, 9.0 mmol) and NaOMe (0.6 mL, 0.5 M in MeOH, 0.30 mmol). The reaction mixture was stirred at room temperature until the solids were dissolved. Then, the Schlenk tube was cooled to -20 °C and a mixture of **4b** (595 mg, 3.0 mmol) and **5a** (3.9 mL, 1.0 M in MeOH, 3.9 mmol) were added. This reaction mixture was then stirred for 32 h at -10 °C. Subsequently, the stirring was turned off and the mixture was cooled to -20 °C, which led to precipitation of solid material. Subsequently, the reaction mixture was transferred with a syringe through a PTFE filter (0.45 µm) to a Schlenk tube containing 20 mL 0.5 M degassed HCl(aq) at 0°C. The aqueous layer was washed with degassed toluene (3 x 2 mL). The combined organic layers were collected in a Schlenk tube and washed with 4 mL degassed phosphate buffer-NaCl solution. After washing, the pH of the solution was 7. Then, the organic phase was washed (3 x 3 mL) with degassed brine. The solution of boronic acid **1b** was stored under Ar atmosphere over 3 Å molecular sieves at -20 °C. By this procedure 5 mL of 0.37 M boronic acid **1b** was obtained (62% yield).

Synthesis of allenyl boronate 1a-Bpin followed by oxidative hydrolysis to allenylboronic acid 1a

Step 1

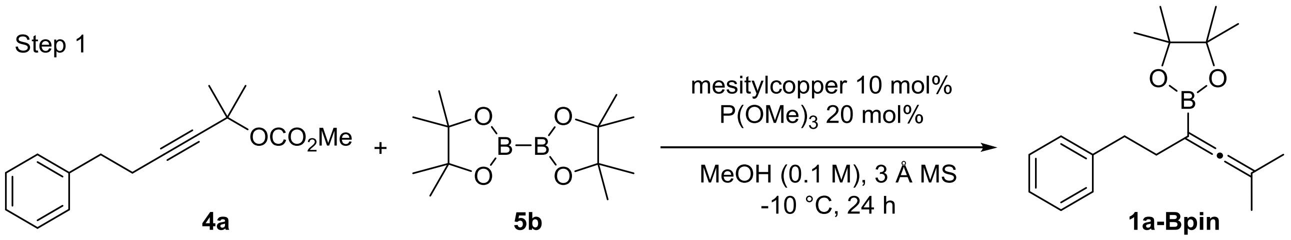

Step 2

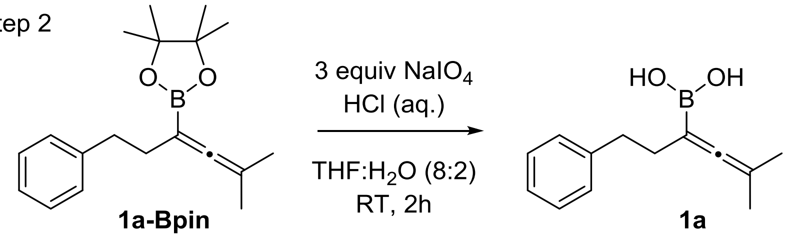

A vial was charged with mesitylcopper(I) (0.03 mmol, 5.5 mg), trimethyl phosphite (0.06 mmol, 7.8 µL), 3 Å molecular sieves, and MeOH (2.5 mL). This reaction mixture was stirred for 20 minutes at room temperature. Then a MeOH solution (0.45 ml) of propargylic carbonate **4a** (0.30 mmol, 74 mg), naphthalene (internal standard, 0.3 mmol 38 mg) and bis(pinacolato)diboron **5b** (0.45 mmol, 114 mg) was added dropwise to the reaction mixture via syringe at -10 °C. Subsequently, this reaction mixture was stirred at -10 °C for 24 hours. Then the reaction mixture was filtered through a 0.45 µm syringe PTFE filter and transferred to 3 ml degassed HCl solution (0.5 M). The resulting slurry was extracted with 1.5 mL toluene and washed once with 1.5 mL phosphate buffer-NaCl solution (prepared from 100 mL commercial phosphate buffer solution (pH = 7) by addition of 10 g NaCl). The toluene solution was placed in a roundbottom flask and the solvent was removed. The resulting oil was dissolved in 2.5 mL degassed THF:H_2O (8:2) under Ar, which was followed by addition of $NaIO_4$ (0.9 mmol, 192 mg). When the $NaIO_4$ had dissolved completely (5 minutes), 0.18 mL 0.5 M HCl was added dropwise. The mixture was stirred at room temperature for two hours, after which it was extracted with 2 mL of degassed toluene. The organic layer was washed with 2 mL degassed phosphate buffer-NaCl solution and 2 mL degassed brine, and then dried in vacuo. The resulting solids were then dissolved in 1 mL degassed toluene-d_8 and sampled for ^1H NMR analysis. This procedure yielded 63% of **1a**.

Synthesis of allenyl boronate 1a-Bnep

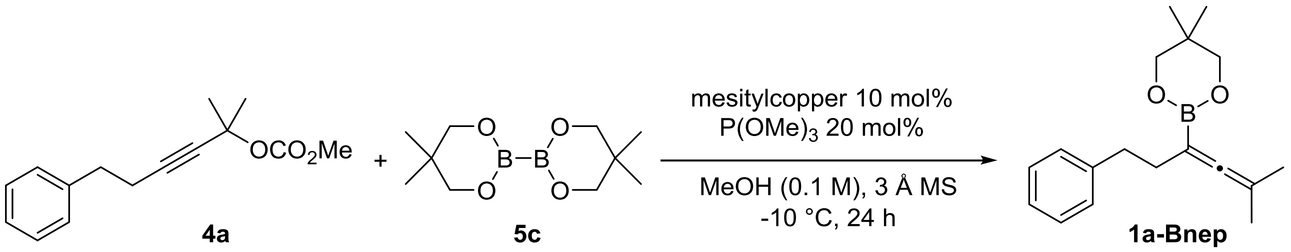

A vial was charged with mesitylcopper(I) (0.03 mmol, 5.5 mg), trimethyl phosphite (0.06 mmol, 7.8 μL), 3 Å molecular sieves, and MeOH (2.5 mL). This reaction mixture was stirred for 20 minutes at room temperature. Then a MeOH solution (0.45 ml) of propargylic carbonate **4a** (0.30 mmol, 74 mg) and bis(neopentyl glycolato)diboron **5c** (0.45 mmol, 102 mg) was added dropwise to the reaction mixture via syringe at -10 °C. Subsequently, this reaction mixture was stirred at -10 °C for 24 hours. Then, the reaction mixture was filtered through a 0.45 μm syringe PTFE filter and transferred to 3 ml degassed HCl solution (0.5 M). The resulting slurry was extracted with 1.5 mL toluene and washed once with 1.5 mL phosphate buffer-NaCl solution (prepared from 100 mL commercial phosphate buffer solution (pH = 7) by addition of 10 g NaCl). The toluene layer was dried by passing through an IST phase separator® (Biotage) and the solvent was removed, affording a colorless oil (71 mg) of **1a-Bnep** and **4a** in a 8 : 2 mass ratio, calculated from ^{1}H NMR analysis. We attempted to purify **1a-Bnep** by silica gel chromatography but these attempts were fuitless because of decomposition of **1a-Bnep** in the presence of silica gel. NMR data for **1a-Bnep**. ^{1}H NMR (400 MHz, $CDCl_3$): δ 7.25-7.18 (m, 5H), 3.62 (s, 4H), 2.69 (t, J = 7.8 Hz, 2H), 2.29 (t, J = 7.8 Hz, 2H), 1.63 (s, 6H), 0.96 (s, 6H);

Synthesis of boronic ester 1a-Bpne

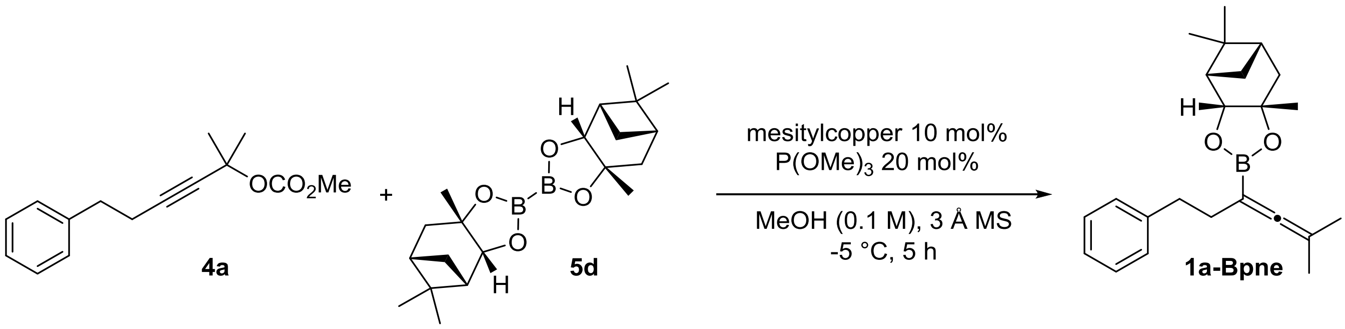

A vial was charged with mesitylcopper(I) (0.02 mmol, 3.7 mg), trimethyl phosphite (0.04 mmol, 4.7 μL), 3 Å molecular sieves, and MeOH (1.5 mL). This reaction mixture was stirred for 10 minutes at room temperature Then a MeOH solution (0.5 ml) of propargylic carbonate **4a** (0.20 mmol, 49 mg) was added dropwise to the reaction mixture via syringe. Subsequently, bis[(+)-pinanediolato]diboron **5d** (0.3 mmol, 107 mg) was added and the reaction mixture was stirred at -5 °C for 5 hours. Then, the reaction mixture was filtered through a 0.45 μm syringe PTFE filter and transferred to 3 ml degassed HCl solution (0.5 M). The resulting slurry was extracted with 1.5 mL toluene and washed once with 1.5 mL phosphate buffer-NaCl solution (prepared from 100 mL commercial phosphate buffer solution (pH = 7) by addition of 10 g NaCl). The toluene layer was dried by passing through an

IST phase separator® (Biotage) and the solvent was removed. Product **1a-Bpne** was isolated in 52% yield (36 mg, 104 μmol) as a colourless oil using petroleum ether/ethyl acetate eluent mixture 50:1 for silica gel chromatography.

$[\alpha]_D^{23}$ -3.409 (*c* 0.44, $CHCl_3$); ^{1}H NMR (400 MHz, $CHCl_3$): δ 7.28-7.23 (m, 2H), 7.21-7.12 (m, 3H), 4.31 (dd, *J* = 8.8 Hz; 1.8 Hz, 1H), 2.73 (t, *J* = 7.7 Hz, 2H), 2.38-2.30 (m, 3H), 2.25-2.18 (m, 1H), 2.08 (t, *J* = 5.5 Hz, 1H), 1.93-1.86 (m, 2H), 1.63 (s, 6H), 1.40 (s, 3H), 1.29 (s, 3H), 1.18 (d, *J* = 10.9 Hz, 1H), 0.85 (s, 3H); ^{13}C NMR (100 MHz, $CHCl_3$): δ 210.9, 142.6, 128.6, 128.1, 125.5, 91.6, 85.8, 77.9, 51.4, 39.6, 38.1, 35.7, 35.6, 32.2, 28.7, 27.1, 26.5, 24.0, 19.8, 19.8; ^{11}B NMR (128 MHz, $CHCl_3$): δ 29.96; HRMS (pos. ESI) m/z: calcd. for $C_{23}H_{31}BO_2Na$ $[M+Na]^+$ 373.2313. Found 373,2322.

Procedure C for reaction of allenylboronic acids with aldehyde, ketones, imines and indole

A vial was charged with the corresponding aldehyde, ketone, imine or indole (0.15 mmol), 3 Å molecular sieves and toluene (0.5 ml). This solution was stirred for 1 min, then the allenylboronic acid (0.10 mmol, obtained by procedure A) in a toluene (0.5 ml) was added to the reaction mixture via syringe. The final reaction mixture was stirred at room temperature. Completion of the reaction was checked by ^{1}H NMR. After a full conversion, the reaction mixture was diluted with diethyl ether (1 mL) under air. The precipitate was filtered off through a short silica pad (about 1 cm in a Pasteur-pipette) using ethyl acetate/hexane (1:1) as an eluent. Then, the solvent was removed and the residue was purified by a rapid silica gel chromatography.

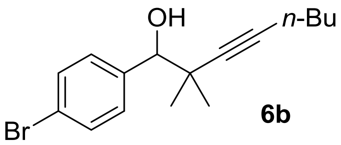

1-(4-Bromophenyl)-2,2-dimethyloct-3-yn-1-ol (6b). This compound was prepared according to procedure C, except that the vial was charged with 0.4 ml of toluene and the allenylboronic acid was added in 0.1 ml toluene. The final reaction mixture was stirred at room temperature for 10 min. The product was isolated by silica gel chromatography using hexane/ethyl acetate 20:1 to 10:1 (v/v) as eluent affording a colorless oil (27 mg, 87%). ^{1}H NMR (400 MHz, $CDCl_3$): δ 7.44 (d, *J* = 8.4 Hz, 2H), 7.26 (d, *J* = 8.4 Hz, 2H), 4.41 (d, *J* = 3.6 Hz, 1H), 2.53 (d, *J* = 4.4 Hz, 1H), 2.20 (t, *J* = 7.2 Hz, 2H), 1.55-1.45 (m, 2H), 1.45-1.33 (m, 2H), 1.21 (s, 3H), 1.02 (s, 3H), 0.92 (t, *J* = 7.2 Hz, 3H); ^{13}C NMR (100 MHz, $CDCl_3$): δ 139.1, 130.6, 129.4, 121.5, 84.2, 83.7, 79.8, 37.9, 31.0, 26.5, 24.5, 21.9, 18.3, 13.6; HRMS (pos. ESI) m/z: calcd. for $C_{16}H_{21}BrONa$ $[M+Na]^+$ 331.0668. Found 331.0657.

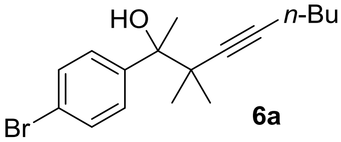

2-(4-Bromophenyl)-3,3-dimethylnon-4-yn-2-ol (6a). This compound was prepared according to procedure C. The final reaction mixture was stirred at room temperature for 24 hours. The product was isolated by silica gel chromatography using hexane/ethyl acetate 100:1 to 30:1 (v/v) as eluent affording a colorless oil (23 mg, 72%). ^{1}H NMR (400 MHz, $CDCl_3$): δ 7.45-7.36 (m, 4H), 2.40 (s, 1H), 2.20 (t, *J* = 6.8 Hz, 2H), 1.68 (s, 3H), 1.54-1.45 (m, 2H), 1.45-1.33 (m, 2H), 1.13 (s, 3H), 1.06 (s, 3H), 0.93 (t, *J* = 7.2 Hz, 3H); ^{13}C NMR (100 MHz, $CDCl_3$): δ 142.6, 130.1, 128.9, 120.8,

85.0, 83.4, 77.1, 41.3, 31.0, 25.7, 25.2(9), 25.2(0), 21.9, 18.3, 13.6; HRMS (pos. ESI) m/z: calcd. for $C_{17}H_{23}BrONa$ $[M+Na]^+$ 347.0824. Found 347.0815.

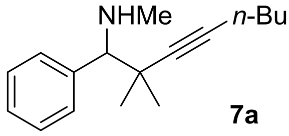

7a

***N*,2,2-trimethyl-1-phenyloct-3-yn-1-amine (7a)**. This compound was prepared according to procedure C. The final reaction mixture was stirred at room temperature for 24 hours. Product **7a** was isolated in 63% yield (15 mg, 63 μmol) as a light-yellow oil by silica gel chromatography using pentane/ethyl acetate eluent mixture 5:1 to 1:1 (v/v). ^{1}H NMR (400 MHz, $CDCl_3$): δ 7.36-7.22 (m, 5H), 3.30 (s, 3H), 2.23 (s, 3H), 2.20 (t, *J* = 6.8 Hz, 2H), 1.80 (br s, 1H), 1.55-1.46 (m, 2H), 1.45-1.36 (m, 2H), 1.21 (s, 3H), 1.00 (s, 3H), 0.94 (t, *J* = 7.2 Hz, 3H); ^{13}C NMR (100 MHz, $CDCl_3$): δ 139.9, 129.1, 127.5, 127.1, 85.9, 82.0, 73.6, 36.3, 35.0, 31.1, 28.7, 25.5, 21.9, 18.4, 13.6; HRMS (pos. ESI) m/z: calcd. for $C_{17}H_{26}N$ $[M+H]^+$ 244.2060. Found 244.2065.

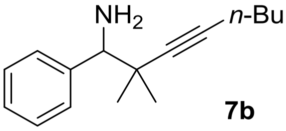

7b

2,2-Dimethyl-1-phenyloct-3-yn-1-amine (7b). This compound was prepared according to procedure C. The final reaction mixture was stirred at room temperature for 24 hours. The product **7b** was isolated The product was isolated by silica gel chromatography using a mixture of hexane/ethyl acetate/Et_3N 100:10:0.5 to 100:50:1 (v/v) as eluent affording a light-yellow oil (19 mg, 83%). ^{1}H NMR (400 MHz, $CDCl_3$): δ 7.41-7.35 (m, 2H), 7.36-7.21 (m, 3H), 3.70 (s, 3H), 2.21 (t, *J* = 6.8 Hz, 2H), 1.82 (br s, 2H), 1.55-1.46 (m, 2H), 1.46-1.35 (m, 2H), 1.26 (s, 3H), 1.02 (s, 3H), 0.93 (t, *J* = 7.2 Hz, 3H); ^{13}C NMR (100 MHz, $CDCl_3$): δ 142.8, 128.1, 127.5, 127.1, 85.2, 82.4, 64.3, 37.1, 31.1, 28.3, 25.8, 21.9, 18.4, 13.6; HRMS (pos. ESI) m/z: calcd. for $C_{16}H_{24}N$ $[M+H]^+$ 230.1903. Found 230.1908.

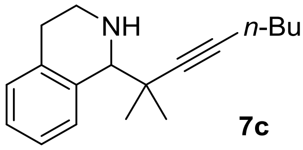

7c

1-(2-Methyloct-3-yn-2-yl)-1,2,3,4-tetrahydroisoquinoline (7c). This compound was prepared according to procedure C. The final reaction mixture was stirred at room temperature for 4 hours. Product **7c** was isolated in 96% yield (25 mg, 96 μmol) as a light-yellow oil by silica gel chromatography using pentane/ethyl acetate eluent mixture 5:1 to 1:1 (v/v). ^{1}H NMR (400 MHz, $CDCl_3$): δ 7.53-7.45 (m, 1H), 7.18-7.05 (m, 3H), 4.01 (s, 1H), 3.43-3.30 (m, 1H), 2.92-2.80 (m, 2H), 2.71-2.60 (m, 1H), 2.15 (t, *J* = 6.8 Hz, 2H), 1.99 (br s, 1H), 1.50-1.40 (m, 2H), 1.40-1.30 (m, 2H), 1.21 (s, 3H), 1.20 (s, 3H), 0.89 (t, *J* = 7.2 Hz, 3H); ^{13}C NMR (100 MHz, $CDCl_3$): δ 137.9, 135.9, 128.8, 128.5, 126.0, 124.7, 87.2, 82.2, 63.4, 41.9, 37.8, 31.0, 30.8, 27.4, 26.2, 21.9, 18.5, 13.6; HRMS (pos. ESI) m/z: calcd. for $C_{18}H_{26}N$ $[M+H]^+$ 256.2060. Found 256.2067.

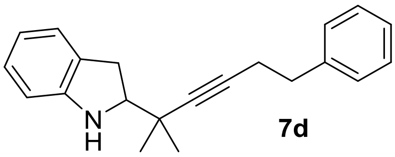

7d

2-(2-Methyl-6-phenylhex-3-yn-2-yl)indoline (7d). This compound was prepared according to procedure C. The final reaction mixture was stirred at room temperature for 48 hours. Product **7d** was isolated in 82% yield (22 mg, 82 μmol) as a light-yellow oil by using silica gel chromatography using pentane/ethyl acetate eluent mixture 100:1 to 30:1 (v/v). ^{1}H NMR (400 MHz, CD_3CN): δ 7.41-7.18 (m, 5H), 7.01 (d, *J* = 7.2 Hz, 1H), 6.96-6.92 (m, 1H), 6.59 (dt, *J* = 7.6, 1.2 Hz, 1H), 6.52 (d, *J* = 7.6 Hz, 1H), 4.51 (br s, 1H), 3.69 (ddd, *J* = 9.6, 9.6, 3.2 Hz, 1H), 2.97 (dd, *J* = 16.0, 9.2 Hz, 1H), 2.87(dd, *J* = 16.0, 9.2 Hz, 1H), 2.72 (t, *J* = 6.8 Hz, 2H), 2.41 (t, *J* = 7.2 Hz, 2H), 1.14 (s, 3H), 1.10 (s, 3H); ^{13}C NMR (100 MHz, CD_3CN): δ 153.2, 142.5, 130.2, 130.1, 129.6, 128.4, 127.5, 125.6, 109.6, 88.0, 82.5, 69.3, 36.9, 36.3, 33.2, 26.6, 26.5, 21.9; HRMS (pos. ESI) m/z: calcd. for $C_{21}H_{24}N$ $[M+H]^+$ 290.1903. Found 290.1912.

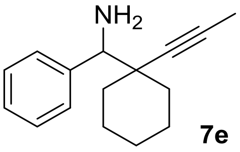

7e

Phenyl(1-(prop-1-yn-1-yl)cyclohexyl)methanamine (7e). This compound was prepared according to procedure C. The final reaction mixture was stirred at room temperature for 48 hours. Product **7e** was isolated in 65% yield (15 mg, 65 μmol) as a light-yellow oil by silica gel chromatography using pentane/ethyl acetate eluent mixture 5:1 to 1:1 (v/v). ^{1}H NMR (400 MHz, $CDCl_3$): δ 7.40-7.35 (m, 2H), 7.32-7.26 (m, 2H), 7.26-7.21 (m, 1H), 3.60 (s, 1H), 2.07 (m, 1H), 1.89 (s, 3H), 1.86 (br s, 2H), 1.73-1.45 (m, 5H), 1.34-1.25 (m, 1H), 1.25-1.13 (m, 1H), 1.13-0.95 (m, 2H); ^{13}C NMR (100 MHz, $CDCl_3$): δ 143.1, 128.2, 127.5, 127.0, 81.6, 80.6, 64.9, 42.9, 36.6, 34.6, 26.0, 23.0, 22.8, 3.6; HRMS (pos. ESI) m/z: calcd. for $C_{16}H_{22}N$ $[M+H]^+$ 228.1747. Found 228.1750.

Procedure D for asymmetric propargylation of ketones A vial was charged with a toluene solution (0.4 mL) of allenylboronic acid (0.10 mmol, obtained by procedure A), (*S*)-Br_2-BINOL (15 μmol, 6.7 mg), 3 Å molecular sieves and EtOH (0.20 mmol, 12.0 μL). This solution was stirred for 3 hours at room temperature, then a toluene solution (0.1 mL) of the ketone (0.15 mmol) was added to the reaction mixture via syringe at room temperature. If needed, toluene was added to adjust the concentration of the allenylboronic acid to 0.2 M. The final reaction mixture was stirred at room temperature for another 48 hours. Completion of the reaction was checked by ^{1}H NMR. After a complete conversion of **1**, the reaction mixture was diluted with MeOH (0.1 mL). The precipitate was filtered off through a short silica pad (about 1 cm in a pipette) using ethyl acetate/hexane (1:1) as an eluent. The solvent was removed and the residue was purified by a rapid silica gel chromatography.

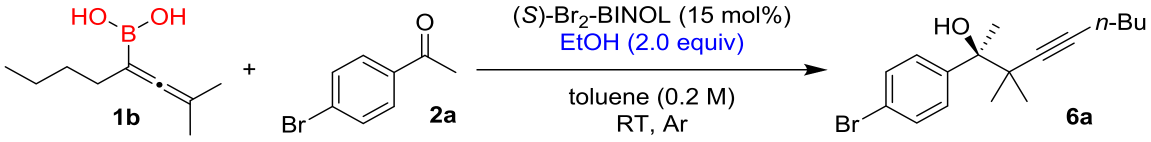

(*R*)-2-(4-Bromophenyl)-3,3-dimethylnon-4-yn-2-ol (6a). This compound was prepared according to procedure D. Product **6a** was isolated in 96% yield (31 mg, 96 μmol) as a colorless oil by silica gel chromatography using hexane/ethyl acetate 100:1 to 30:1 (v/v) as eluent.

$[\alpha]_D^{22}$ -24.1 (*c* 0.70, $CHCl_3$).

Determination of *ee*: Chiral SFC (Diacel IC, 35 bar, 40 ºC, 0.46 cm ϕ, 25 cm column, 5% MeOH in CO_2, flow rate: 2.0 mL/min; R_t: 3.20 min (minor enantiomer), 3.67 min (major enantiomer); *ee* (major enantiomer) = 94%.

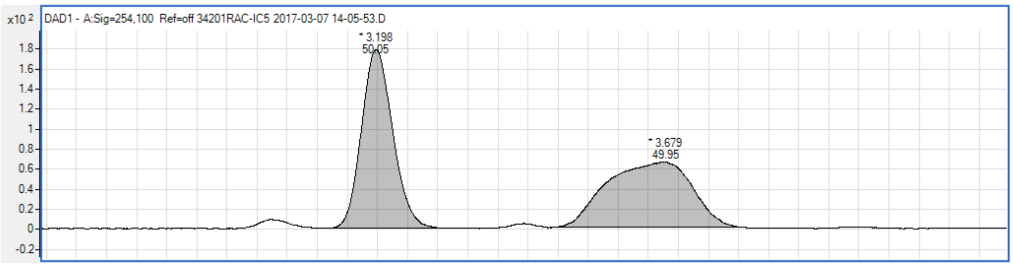

Integration Peak List

Peak	Start	RT	End	Height	Area	AreaSumPercent
1	3.119	3.198	3.309	178.17	642.96	50.05
2	3.494	3.679	3.809	65.1	641.76	49.95

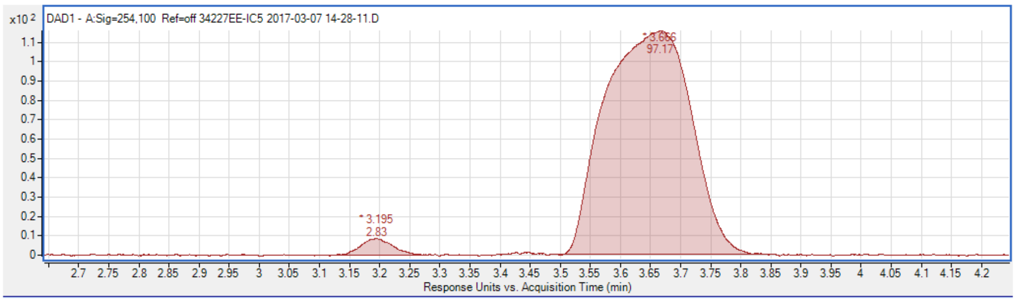

Integration Peak List

Peak	Start	RT	End	Height	Area	AreaSumPercent
1	3.117	3.195	3.278	8.7	33.96	2.83
2	3.496	3.666	3.845	115.53	1165.39	97.17

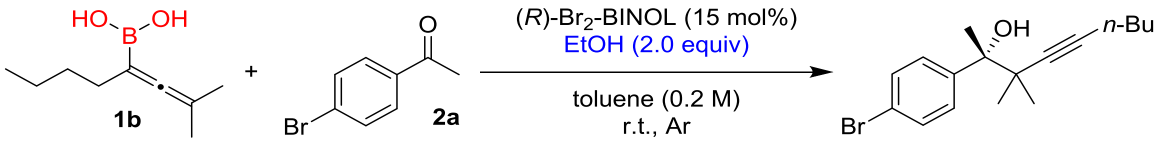

(*S*)-2-(4-bromophenyl)-3,3-dimethylnon-4-yn-2-ol (Table 5, entry 4). This compound was prepared according to procedure D, except (*R*)-Br_2-BINOL was used. The product was isolated in 94% yield (30 mg, 94 μmol) as a colorless oil by silica gel chromatography using pentane/ethyl acetate eluent mixture 100:1 to 30:1 (v/v). $[\alpha]_D^{23}$ +25.2 (*c* 0.51, $CHCl_3$).

Determination of *ee*: Chiral SFC (Diacel IC, 35 bar, 40 ºC, 0.46 cm ϕ, 25 cm column, 5% MeOH in CO_2, flow rate: 2.0 mL/min; t_R: 3.20 min (major enantiomer), 3.68 min (minor enantiomer); *ee* (major enantiomer) = 94%.

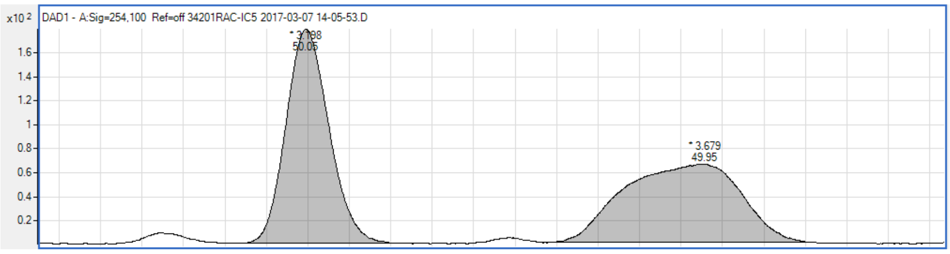

Integration Peak List

Peak	Start	RT	End	Height	Area	AreaSumPercent
1	3.119	3.198	3.309	178.17	642.96	50.05
2	3.494	3.679	3.809	65.1	641.76	49.95

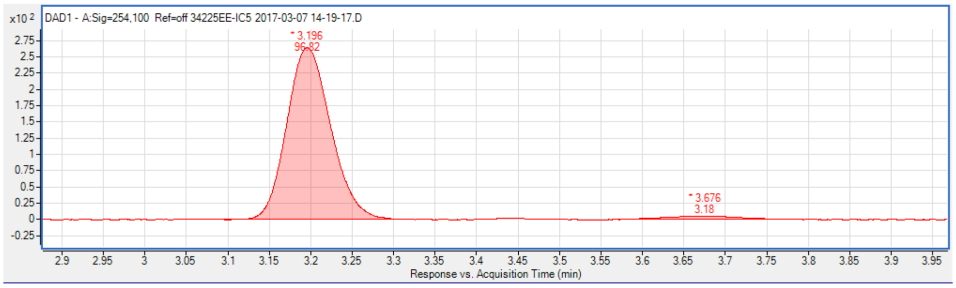

Integration Peak List

Peak	Start	RT	End	Height	Area	AreaSumPercent
1	3.094	3.196	3.322	264.48	975.97	96.82
2	3.574	3.676	3.786	5.12	32.06	3.18

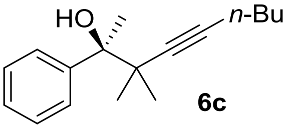

6c

(*R*)-3,3-Dimethyl-2-phenylnon-4-yn-2-ol (6c). This compound was prepared according to procedure D with (*S*)-Br_2-BINOL (20 μmol, 8.9 mg) catalyst and using a reaction time of 72 hours. Product **6c** was isolated in 75% yield (18 mg, 75 μmol) as a colorless oil by silica gel chromatography using pentane/ethyl acetate eluent mixture 30:1 to 10:1 (v/v). $[\alpha]_D^{21}$ -27.7 (*c* 0.40, $CHCl_3$); ^1H NMR (400 MHz, $CDCl_3$): δ 7.54-7.49 (m, 2H), 7.34-7.27 (m, 2H), 7.27-7.21 (m, 1H), 2.44 (s, 1H), 2.21 (t, *J* = 7.2 Hz, 2H), 1.72 (s, 3H), 1.57-1.45 (m, 2H), 1.45-1.36 (m, 2H), 1.16 (s, 3H), 1.07 (s, 3H), 0.93 (t, *J* = 7.6 Hz, 3H); ^{13}C NMR (100 MHz, $CDCl_3$): δ 143.3, 127.0, 126.6, 85.4, 83.0, 77.2, 41.5, 31.0, 25.8, 25.4, 25.3, 21.9, 18.4, 13.6; HRMS (pos. ESI) m/z: calcd. for $C_{17}H_{24}ONa$ $[M+Na]^+$ 267.1719. Found 267.1731.

Determination of *ee*: Chiral SFC (Diacel IC, 35 bar, 40 ºC, 0.46 cm *ϕ*, 25 cm column, 5% MeOH in CO_2, flow rate: 2.0 mL/min; R_t: 2.82 min (minor enantiomer), 3.08 min (major enantiomer); *ee* (major enantiomer) = 97%.

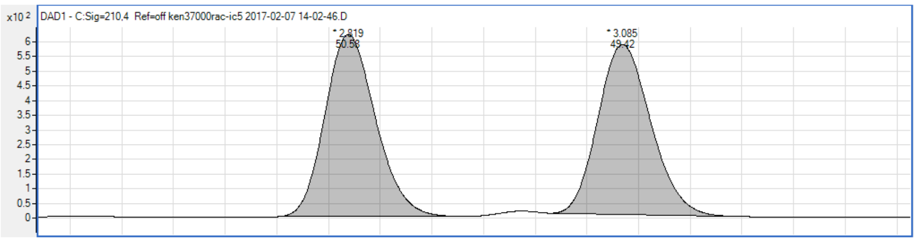

Integration Peak List

Peak	Start	RT	End	Height	Area	AreaSumPercent
1	2.753	2.819	2.925	617.84	2103.59	50.58
2	3.017	3.085	3.198	576.74	2055.64	49.42

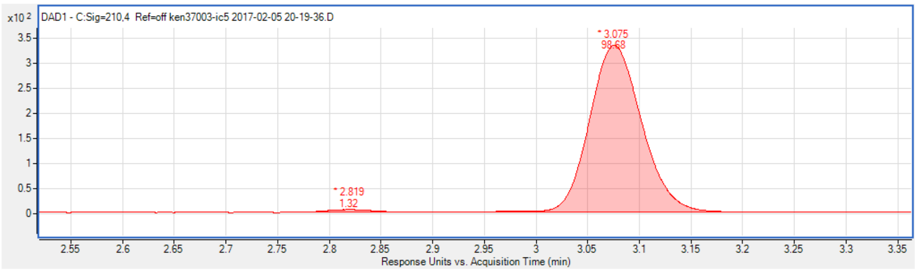

Integration Peak List

Peak	Start	RT	End	Height	Area	AreaSumPercent
1	2.765	2.819	2.894	5.16	15.48	1.32
2	2.959	3.075	3.198	331.84	1159.86	98.68

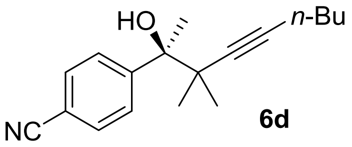

6d

(*R*)-4-(2-Hydroxy-3,3-dimethylnon-4-yn-2-yl)benzonitrile (6d). This compound was prepared according to procedure D with a reaction time of 72 hours. Product **6d** was isolated in 67% yield (18 mg, 67 μmol) as a colorless oil using by silica gel chromatography pentane/ethyl acetate eluent mixture 20:1 to 10:1 (v/v). $[\alpha]_D^{23}$ -12.9 (*c* 0.23, $CHCl_3$); ^{1}H NMR (400 MHz, $CDCl_3$): δ 7.65 (d, *J* = 8.4 Hz, 2H), 7.59 (d, *J* = 8.4 Hz, 2H), 2.44 (s, 1H), 2.20 (t, *J* = 7.2 Hz, 2H), 1.71 (s, 3H), 1.54-1.46 (m, 2H), 1.45-1.35 (m, 2H), 1.13 (s, 3H), 1.07 (s, 3H), 0.93 (t, *J* = 7.2 Hz, 3H); ^{13}C NMR (100 MHz, $CDCl_3$): δ 149.2, 130.9, 127.9, 119.0, 110.5, 84.6, 83.8, 77.2, 41.3, 30.9, 25.6, 25.2, 25.1, 21.9, 18.3, 13.5; HRMS (pos. ESI): *m/z* calcd. for $C_{18}H_{23}NONa$ $[M+Na]^+$ 292.1672. Found 292.1684.

Determination of *ee*: Chiral SFC (Diacel IA, 35 bar, 40 °C, 0.46 cm *ϕ*, 25 cm column, 5% MeOH in CO_2, flow rate: 2.0 mL/min; R_t: 5.14 min (minor enantiomer), 5.39 min (major enantiomer); *ee* (major enantiomer) = 91%.

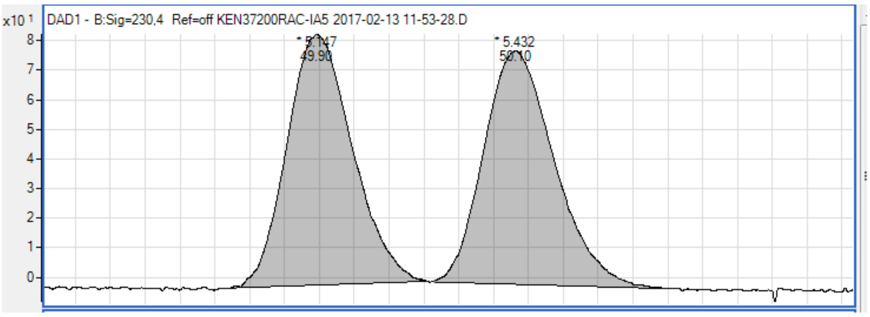

Integration Peak List

Peak	Start	RT	End	Height	Area	AreaSumPercent
1	5.01	5.147	5.304	84.61	553.79	49.9
2	5.31	5.432	5.644	78.81	556.12	50.1

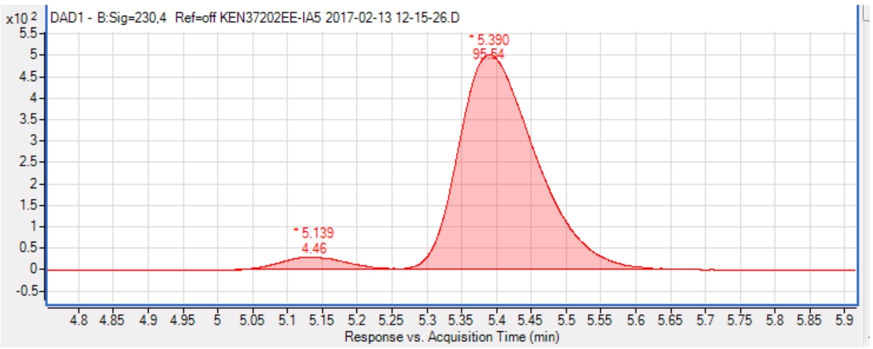

Integration Peak List

Peak	Start	RT	End	Height	Area	AreaSumPercent
1	5.007	5.139	5.253	28.75	177.54	4.46
2	5.26	5.39	5.626	499.81	3806.05	95.54

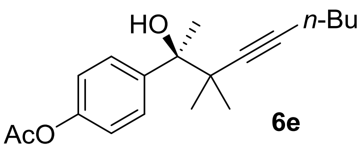

(*R*)-4-(2-Hydroxy-3,3-dimethylnon-4-yn-2-yl)phenyl acetate (6e). This compound was prepared according to procedure D. Product **6e** was isolated in 90% yield (27.3 mg, 90 μmol) as colorless oil by silica gel chromatography using pentane/ethyl acetate 5:1 as eluent. $[\alpha]_D^{22}$ -30.4 (*c* 0.25, $CHCl_3$); ^{1}H NMR (400 MHz, $CDCl_3$): δ 7.51 (d, *J* = 8.8 Hz, 2H), 7.02 (d, *J* = 8.8 Hz, 2H), 2.40 (s, 1H), 2.29 (s, 3H), 2.20 (t, *J* = 6.98 Hz, 2H), 1.71 (s, 3H), 1.54-1.47 (m, 2H), 1.46-1.36 (m, 2H), 1.15 (s, 3H), 1.07 (s, 3H), 0.93 (t, 3H); ^{13}C NMR (100 MHz, $CDCl_3$): δ 169.4, 149.5, 141.0, 128.2, 120.0, 85.3, 83.2, 41.5, 31.3, 25.9, 25.4, 25.3, 22.0, 21.2, 18.4, 13.6; HRMS (pos. ESI) m/z: calcd for $C_{19}H_{26}O_3Na$ $[M+Na]^+$ 325.1774. Found 325.1767.

Determination of *ee*: Chiral SFC (Diacel IA, 35 bar, 40 °C, 0.46 cm ϕ, 25 cm column, 15% MeOH in CO_2, flow rate: 2.0 mL/min; R_t: 2.72 min (minor enantiomer), 2.92 min (major enantiomer); *ee* (major enantiomer) = 96%.

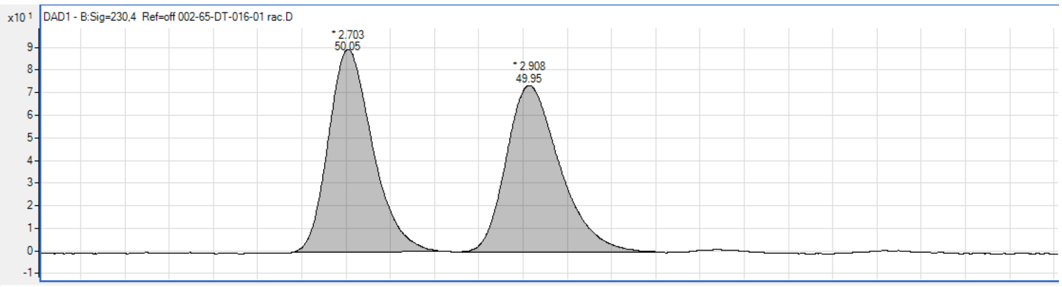

Integration Peak List

Peak	Start	RT	End	Height	Area	AreaSumPercent
1	2.639	2.703	2.812	89.62	310.72	50.05
2	2.828	2.908	3.051	73.65	310.05	49.95

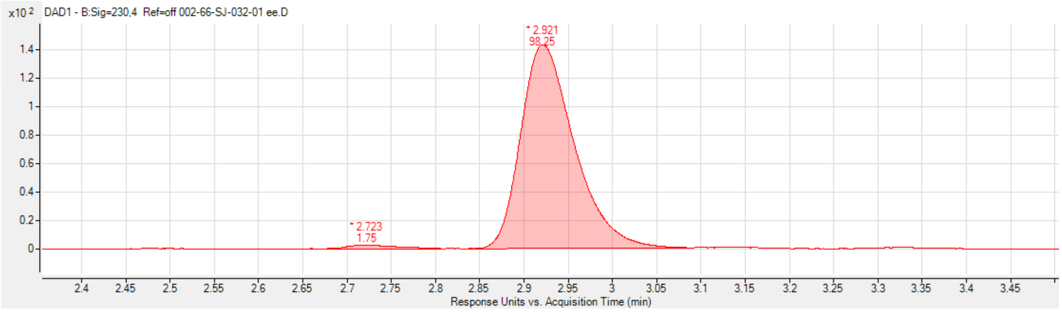

Integration Peak List

Peak	Start	RT	End	Height	Area	AreaSumPercent
1	2.654	2.723	2.807	2.59	10.74	1.75
2	2.833	2.921	3.085	143.38	603.93	98.25

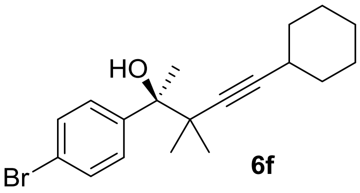

6f

(*R*)-2-(4-bromophenyl)-5-cyclohexyl-3,3-dimethylpent-4-yn-2-ol (6f) This compound was prepared according to procedure D with a reaction time of 72 hours. Product **6f** was isolated in 77% yield (27 mg, 77 μmol) as a colorless oil using pentane/ethyl acetate eluent mixture 30:1 to 10:1 (v/v) for silica gel chromatography. $[\alpha]_D^{23}$ -23.7 (*c* 0.40, $CHCl_3$); ^1H NMR (400 MHz, $CDCl_3$): 7.45-7.36 (m, 4H), 2.43 (s, 1H), 2.42 (m, 1H), 1.84-1.74 (m, 2H), 1.74-1.62 (m, 2H), 1.68 (s, 3H), 1.53-1.37 (m, 3H), 1.37-1.25 (m, 3H), 1.13 (s, 3H), 1.06 (s, 3H); ^{13}C NMR (100 MHz, $CDCl_3$): δ 142.6, 130.1, 128.9, 120.8, 87.6, 85.0, 77.0, 41.2, 32.9, 28.9, 25.9, 25.6, 25.3, 25.2, 24.7; HRMS (pos. ESI) m/z: calcd. for $C_{19}H_{25}BrONa$ $[M+Na]^+$ 371.0981. Found 371.0979.

Determination of *ee*: Chiral SFC (Diacel IC, 35 bar, 40 °C, 0.46 cm *ϕ*, 25 cm column, 5% MeOH in CO_2, flow rate: 2.0 mL/min; t_R: 3.92 min (minor enantiomer), 4.72 min (major enantiomer); *ee* (major enantiomer) = 90%.

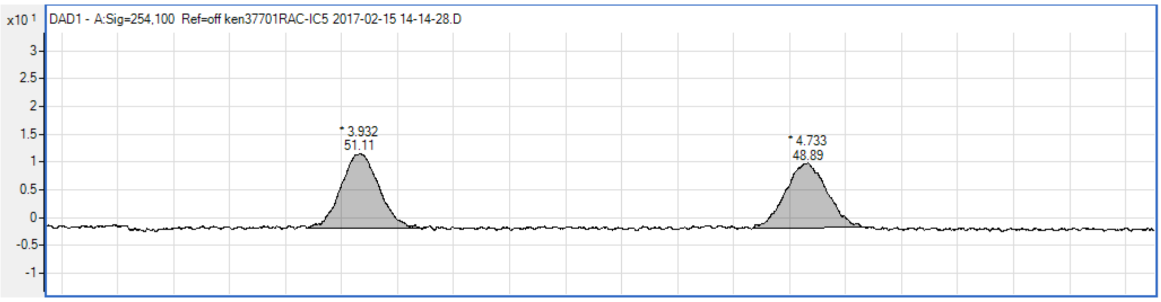

Integration Peak List

Peak	Start	RT	End	Height	Area	AreaSumPercent
1	3.84	3.932	4.039	13.31	62.5	51.11
2	4.636	4.733	4.845	11.62	59.77	48.89

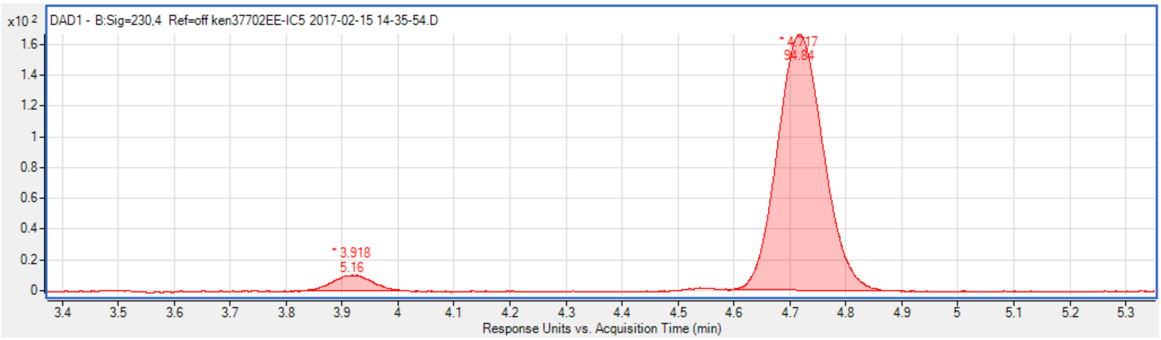

Integration Peak List

Peak	Start	RT	End	Height	Area	AreaSumPercent
1	3.827	3.918	4.008	10.21	51.62	5.16
2	4.576	4.717	4.894	166.21	949.05	94.84

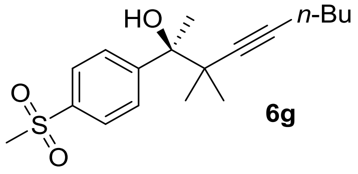

(*R*)-3,3-Dimethyl-2-(4-(methylsulfonyl)phenyl)non-4-yn-2-ol (6g) This compound was prepared according to procedure D with a reaction time of 90 hours. Product **6g** was isolated in 62% yield (0.10 mmol scale, 20 mg, 0.06 mmol) and 70% yield (0.50 mmol scale, 112 mg, 0.35 mmol) as colorless oil by silica gel chromatography using pentane/ethyl acetate 5:1 to 2:1 (v/v) as eluent. $[\alpha]_D^{21}$ -11.2 (*c* 0.41, $CHCl_3$); ^{1}H NMR (400 MHz, $CDCl_3$): δ 7.87 (d, *J* = 8.7 Hz, 2H), 7.74 (d, *J* = 8.7 Hz, 2H), 3.06 (s, 3H), 2.47 (s, 1H), 2.21 (t, *J* = 7.0 Hz, 2H), 1.73 (s, 3H), 1.52-1.47 (m, 2H), 1.45-1.36 (m, 2H), 1.15 (s, 3H), 1.09 (s, 3H), 0.93 (t, *J* = 7.24 Hz, 3H); ^{13}C NMR (100 MHz, $CDCl_3$): δ 150.3, 138.8, 128.2, 126.2, 84.6, 83.9, 44.5, 41.4, 31.0, 25.8, 25.2, 25.2, 22.0, 18.4, 13.6; HRMS (pos. ESI) m/z: calcd for $C_{18}H_{28}O_3SNa$ $[M+Na]^+$ 345.1495. Found 345.1479.

Determination of *ee* (0.1 mmol scale): Chiral SFC (Diacel IC, 35 bar, 40 °C, 0.46 cm ϕ, 25 cm column, 15% MeOH in CO_2, flow rate: 2.0 mL/min; *t*R: 5.29 min (minor enantiomer), 6.10 min(major enantiomer); *ee* (major enantiomer) = 94%.

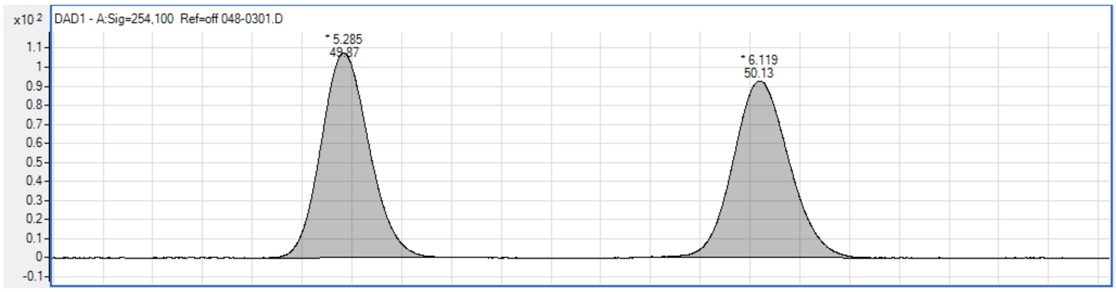

Integration Peak List

Peak	Start	RT	End	Height	Area	AreaSumPercent
1	5.103	5.285	5.501	107.46	721.23	49.87
2	5.896	6.119	6.373	92.7	725.02	50.13

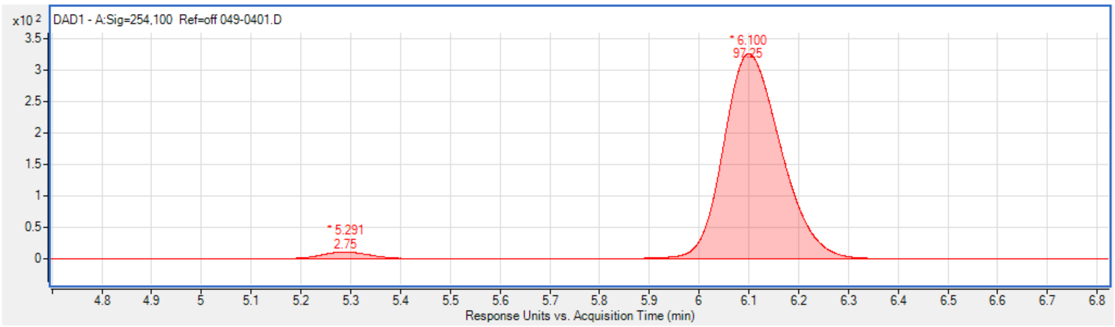

Integration Peak List

Peak	Start	RT	End	Height	Area	AreaSumPercent
1	5.142	5.291	5.445	11	73.48	2.75
2	5.873	6.1	6.357	325.82	2602.64	97.25

Determination of *ee* (0.5 mmol scale): Chiral SFC (Diacel IC, 35 bar, 40 °C, 0.46 cm ϕ, 25 cm column, 15% MeOH in CO_2, flow rate: 2.0 mL/min; R_t: 5.23 min (minor enantiomer), 6.07 min (major enantiomer); *ee* (major enantiomer) = 96%

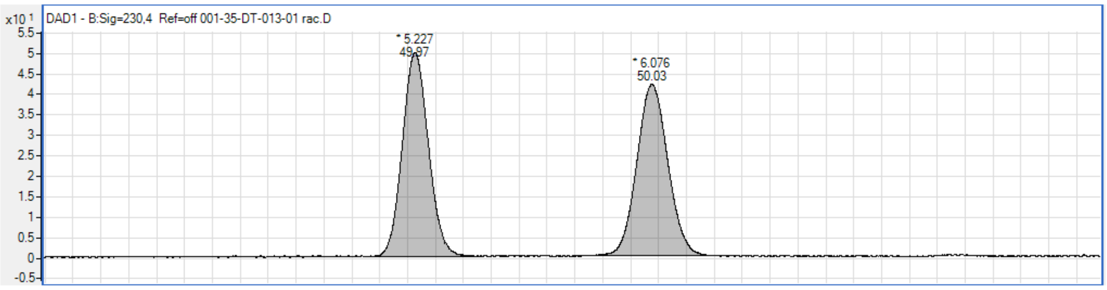

Integration Peak List

Peak	Start	RT	End	Height	Area	AreaSumPercent
1	5.045	5.227	5.442	49.71	322.55	49.97
2	5.865	6.076	6.332	41.82	322.95	50.03

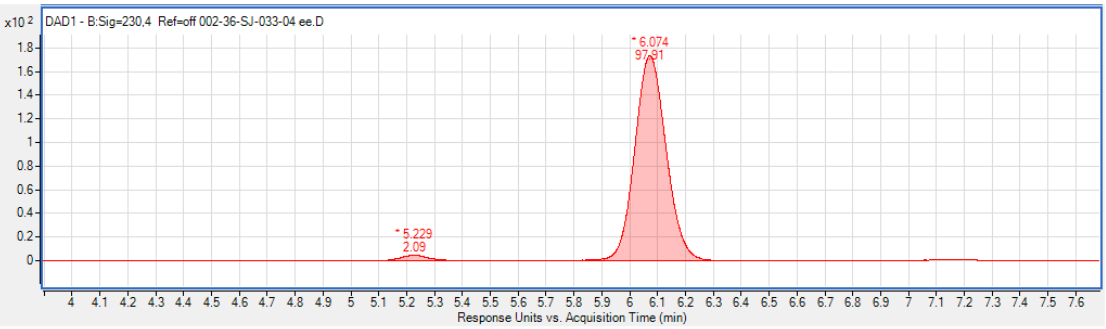

Integration Peak List

Peak	Start	RT	End	Height	Area	AreaSumPercent
1	5.094	5.229	5.379	4.43	28.71	2.09
2	5.846	6.074	6.323	173.23	1347.47	97.91

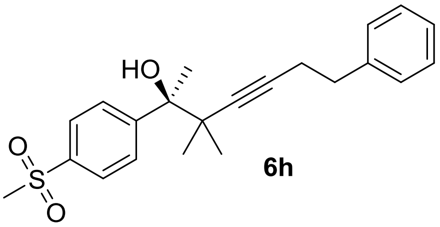

6h

(*R*)-3,3-Dimethyl-2-(4-(methylsulfonyl)phenyl)-7-phenylhept-4-yn-2-ol (6h). This compound was prepared according to procedure D with a reaction time of 72 hours. Product **6h** was isolated in 63% yield (23 mg, 63 μmol) as a colorless oil by silica gel chromatography using pentane/ethyl acetate eluent mixture 10:1 to 2:1 (v/v). $[\alpha]_D^{22}$ -14.6 (*c* 0.58, $CHCl_3$); ^1H NMR (400 MHz, $CDCl_3$): δ 7.82 (d, *J* = 8.8 Hz, 2H), 7.60 (d, *J* = 8.4 Hz, 2H), 7.36-7.29 (m, 2H), 7.26-7.20 (m, 3H), 3.03 (s, 3H), 2.83 (t, *J* = 7.2 Hz, 2H), 2.53 (t, *J* = 7.2 Hz, 2H), 2.31 (s, 1H), 1.60 (s, 3H), 1.10 (s, 3H), 1.05 (s, 3H); ^{13}C NMR (100 MHz, $CDCl_3$): δ 150.2, 140.5, 138.6, 128.43, 128.41, 128.0, 126.3, 126.0, 85.7, 82.7, 77.1, 44.4, 41.2, 34.9, 25.4, 25.0(9), 25.0(3), 20.6; HRMS (pos. ESI) m/z: calcd. for $C_{22}H_{26}O_3SNa$ $[M+Na]^+$ 393.1495. Found 393.1507.

Determination of *ee*: Chiral SFC (Diacel IC, 35 bar, 40 °C, 0.46 cm ϕ, 25 cm column, 15% MeOH in CO_2, flow rate: 2.0 mL/min; R_t: 9.35 min (minor enantiomer), 10.94 min (major enantiomer); *ee* (major enantiomer) = 96%.

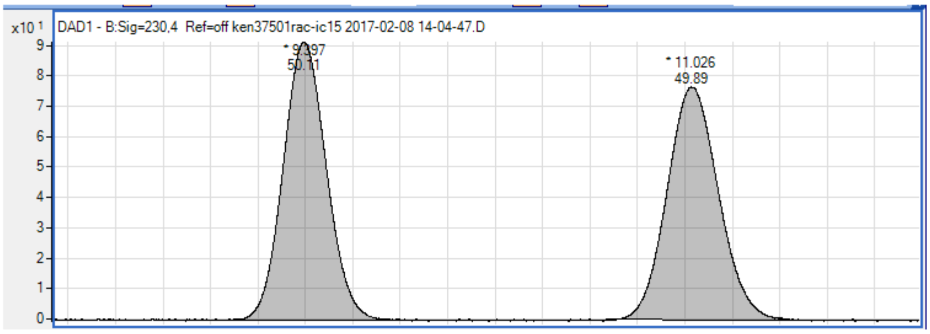

Integration Peak List

Peak	Start	RT	End	Height	Area	AreaSumPercent
1	9.082	9.397	9.853	91.51	1159.28	50.11
2	10.673	11.026	11.485	76.6	1153.97	49.89

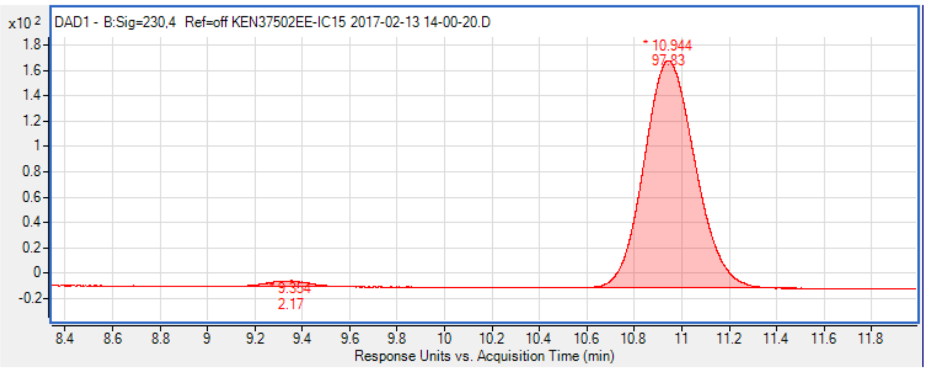

Integration Peak List

Peak	Start	RT	End	Height	Area	AreaSumPercent
1	9.115	9.354	9.626	4.68	59.49	2.17
2	10.596	10.944	11.476	179.09	2687.27	97.83

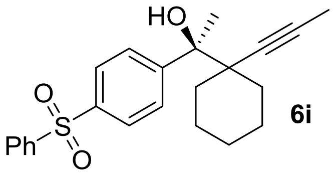

(*R*)-1-(4-(phenylsulfonyl)phenyl)-1-(1-(prop-1-yn-1-yl)cyclohexyl)ethan-1-ol (6i) This compound was prepared according to procedure D with a catalyst loading of (*S*)-Br_2-BINOL (20 µmol, 8.9 mg) and a reaction time of 72 hours. The concentration of allenylboronic acid was 0.1 M. Product **6i** was isolated in 64% yield (24.5 mg, 64 µmol) as a colorless oil by silica gel chromatography using pentane/ethyl acetate eluent mixture 10:1 to 5:1 (v/v). $[\alpha]_D^{23}$-6.9 (*c* 0.27, $CHCl_3$); ^{1}H NMR (400 MHz, $CDCl_3$): δ 7.98-7.93 (m, 2H), 7.86 (d, *J* = 8.8 Hz, 2H), 7.63 (d, *J* = 8.8 Hz, 2H), 7.60-7.48 (m, 3H), 2.46 (s, 1H), 1.88 (s, 3H), 1.79-1.71 (m, 1H), 1.66 (s, 3H), 1.62-1.48 (m, 6H), 1.25 (m, 1H), 0.93-0.84 (m, 2H); ^{13}C NMR (100 MHz, $CDCl_3$): δ 150.0, 141.7, 139.6, 133.0, 129.2, 128.2, 127.6, 126.4, 82.3, 81.1, 77.7, 47.5, 31.9, 30.3, 25.5, 25.4, 23.2, 23.0, 3.5; HRMS (pos. ESI) m/z: calcd. for $C_{23}H_{26}O_3SNa$ $[M+Na]^+$ 405.1495. Found 405.1501.

Determination of *ee*: Chiral SFC (Diacel IC, 35 bar, 40 °C, 0.46 cm ϕ, 25 cm column, 15% MeOH in CO_2, flow rate: 2.0 mL/min; R_t: 14.98 min (minor enantiomer), 18.73 min (major enantiomer); *ee* (major enantiomer) = 99%.

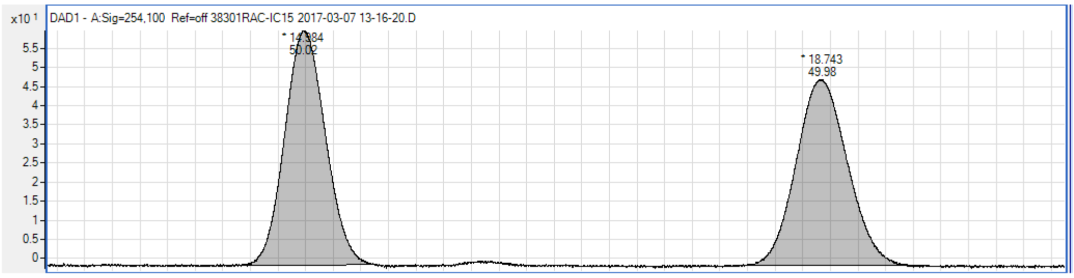

Integration Peak List

Peak	Start	RT	End	Height	Area	AreaSumPercent
1	14.459	14.984	15.493	61.32	1260.12	50.02
2	18.103	18.743	19.505	48.66	1258.97	49.98

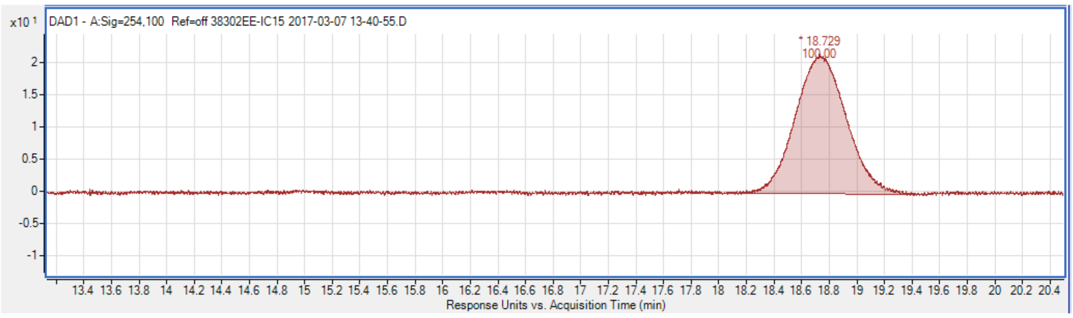

Integration Peak List

Peak	Start	RT	End	Height	Area	AreaSumPercent
1	18.073	18.729	19.509	21.73	554.14	100

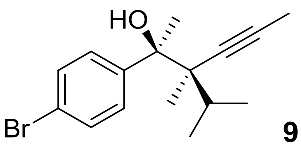

9

(2R,3R)-2-(4-bromophenyl)-3-isopropyl-3-methylhex-4-yn-2-ol (9) A vial was charged with a toluene solution (0.33 mL) of allenylboronic acid **1g** (0.10 mmol, 1.00 equiv.), (*S*)-Br_2-BINOL (44.4 mg, 0.10 mmol, 1.00 equiv.) and 3 Å molecular sieves. This solution was stirred for 1 hour at room temperature, then *p*-Bromoacetophenone (39.8 mg, 0.20 mmol, 2.00 equiv.) was added to the reaction. The reaction mixture was stirred at 45 °C for another 22 hours. Product **9** was isolated in 31% yield (9.7 mg, 31 μmol) as a colorless oil by silica gel chromatography using petroleum ether/ethyl acetate eluent mixture 100:1 (v/v).

$[\alpha]_D^{22}$ –71.0 (*c* 0.10, $CHCl_3$); ^{1}H NMR (400 MHz, $CDCl_3$): δ 7.45-7.38 (m, 4H), 2.59 (bs, 1H), 1.88 (s, 3H), 1.68 (s, 3H), 1.56 (hept, J = 6.7 Hz, 1H), 1.17 (s, 3H), 1.05 (d, J = 6.6 Hz, 3H), 0.62 (d, J = 6.7 Hz, 3H). ^{13}C NMR (100 MHz, $CDCl_3$): δ 143.5, 130.9, 128.9, 120.9, 82.0, 81.1, 78.6, 49.8, 32.4, 28.3, 21.7, 20.7, 19.9, 3.7; HRMS (pos. ESI) m/z: calcd. for $C_{16}H_{21}BrONa$ $[M+Na]^+$ 331.0655. Found 331.0668.

Determination of *ee*: Chiral SFC (Diacel IC, 35 bar, 40 °C, 0.46 cm ϕ, 25 cm column, 10% MeOH in CO_2, flow rate: 2.0 mL/min; R_t: 2.69 min (minor enantiomer), 2.93 min (major enantiomer); *ee* (major enantiomer) = 96%.

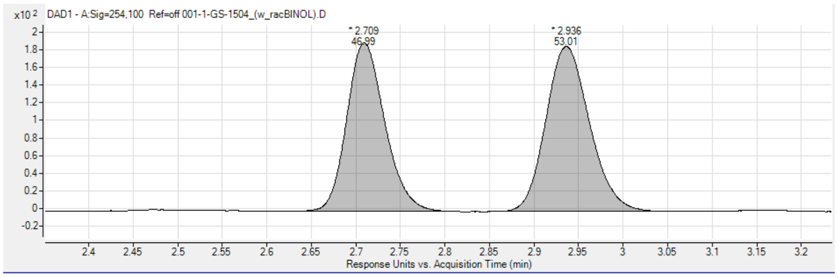

Integration Peak List

Peak	Start	RT	End	Height	Area	AreaSumPercent
1	2.63	2.709	2.805	190.38	564.32	46.99
2	2.856	2.936	3.062	186.5	636.52	53.01

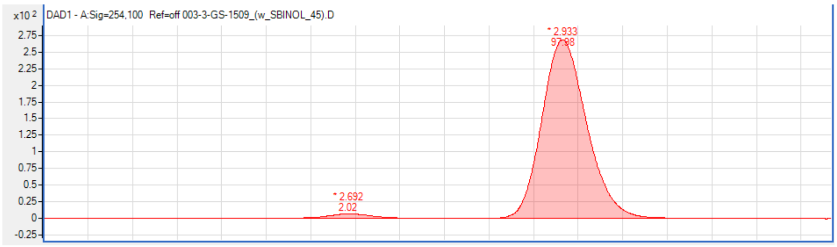

Integration Peak List

Peak	Start	RT	End	Height	Area	AreaSumPercent
1	2.638	2.692	2.753	6.49	19.25	2.02
2	2.842	2.933	3.073	268.23	933.05	97.98

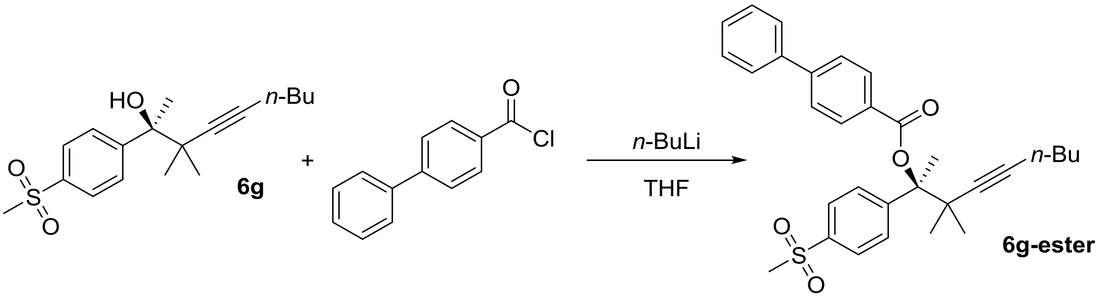

(R)-3,3-dimethyl-2-(4-(methylsulfonyl)phenyl)non-4-yn-2-yl [1,1'-biphenyl]-4-carboxylate (6g-ester). Esterification of **6g** was carried out using a modified literature procedure.[5] Compound **6g** (20 mg, 0.06 mmol) was dissolved in 0.5 mL of dry THF and placed in an Ar-filled round bottom flask. This mixture was cooled to -78 °C and *n*-BuLi (30 µL of 2.5 M in hexane, 0.08 mmol) was added under stirring; then the resulted mixture was stirred for 1 hour at -78 °C. Subsequently, biphenyl-4-carbonyl chloride (34 mg, 0.16 mmol) in dry THF (0.2 mL) was added. The resulting mixture was stirred for 2 hours at 60 °C; and then quenched with sat. NH_4Cl. The water phase was extracted with Et_2O (3 x 2 mL), and the organic phases were collected and dried by passing through an IST phase separator® (Biotage). Product **6g-ester** was isolated by silica gel chromatography (using petroleum ether/ethyl acetate 5:2 as eluent) as a colorless solid in 71% yield (22 mg, 0.04 mmol). The amorphous solid (10 mg) was dissolved in 0.5 mL of ethanol at room temperature and water (60 µL) was added dropwise. The solution was kept at 7 °C for 96 hours affording colorless crystals, which were suitable for X-ray diffraction analysis. Melting point 131.6 °C. $[\alpha]_D^{22}$ +113.704 (*c* 0.27, $CHCl_3$); ^{1}H NMR (400 MHz, $CDCl_3$): δ 8.16 (d, J = 8.5 Hz, 2H), 7.86 (d, J = 9.0 Hz, 2H), 7.71 (d, J = 8.5 Hz, 2H), 7.65-7.63 (m, 2H), 7.55-7.47 (m, 4H), 7.44-7.40 (m, 1H), 3.06 (s, 3H), 2.21 (s, 3H), 2.17 (t, J = 7.0 Hz, 2H), 1.51-1.42 (m, 2H), 1.40 (s, 3H), 1.38-1.33 (m, 2H), 1.28 (s, 3H), 0.90 (t, J = 7.2 Hz, 3H); ^{13}C NMR (100 MHz, $CDCl_3$): δ 164.7, 147.4, 146.1, 139.9, 139.0, 130.2, 129.6, 129.0, 128.3, 128.0, 127.3, 127.3, 126.1, 86.4, 84.1, 83.7, 44.6, 41.5, 31.0, 25.4, 25.3, 22.0, 22.0, 18.4, 13.6; HRMS (pos. ESI) m/z: calcd for $C_{31}H_{34}O_4SNa$ $[M+Na]^+$ 525.2070. Found 525.2079.

Determination of *ee*: Chiral SFC (Diacel IB, 35 bar, 40 °C, 0.46 cm ϕ, 25 cm column, 15% MeOH in CO_2, flow rate: 2.0 mL/min; R_t: 6.66 min (minor enantiomer), 7.33 min (major enantiomer); *ee* (major enantiomer) = 94%.

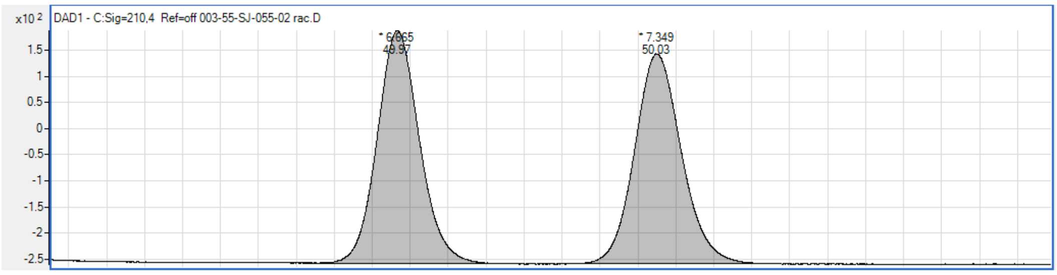

Integration Peak List

Peak	Start	RT	End	Height	Area	AreaSumPercent
1	6.381	6.665	6.931	444.72	3438.77	49.97
2	7.115	7.349	7.63	401.49	3443.47	50.03

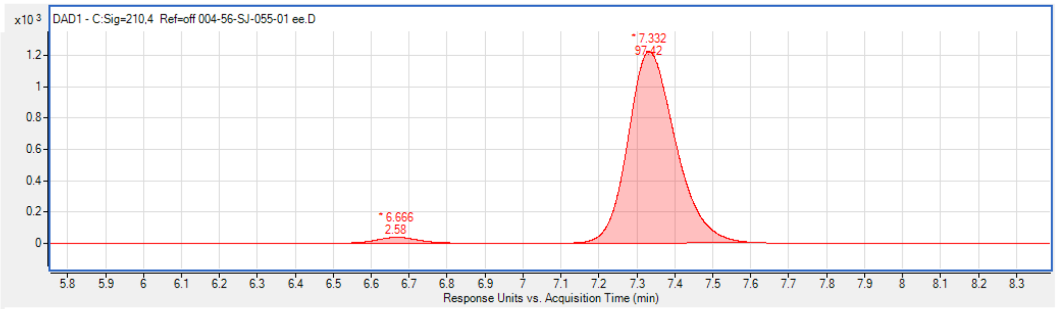

Integration Peak List

Peak	Start	RT	End	Height	Area	AreaSumPercent
1	6.523	6.666	6.844	37.82	283.02	2.58
2	7.126	7.332	7.631	1226.86	10692.77	97.42

The crystallographic data for **6g-ester** is given in file: 6g_ester.cif. The crystal structure has been deposited at the Cambridge Crystallographic Data Centre and has been assigned deposition number: CCDC 1550097.

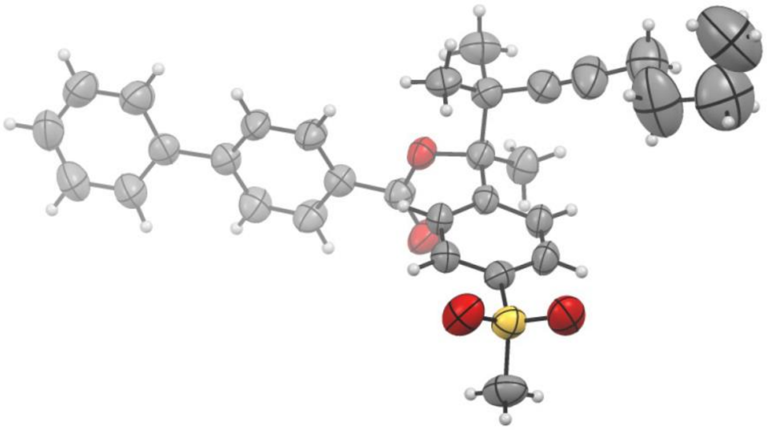

ORTEP model of the X-ray crystal structure of **6g-ester**.

Explanation of the A-level alert in the checkif file of 6g-ester

The checkif file (6g_ester_checkif.pdf) reports an A-level alert stating that ”Structure Contains Solvent Accessible VOIDS”. As it appears from the crystal packing structure (6g_ester_cavity_at_origin.png) these voids are part of the unit cell. The largest residual density that was found in these void channels is 0.75 e/Å^3. Partial occupation of these channels by disordered solvent molecules is a possible explanation for this residual density. Since the residual density is low, these channels are largely empty. In conclusion, the voids do not effect the accuracy of the structural parameters determined from the X-ray data. The Flack parameter is x = -0.01(2), thus the absolute configuration of **6g-ester** can be unambiguously assigned as *R*.

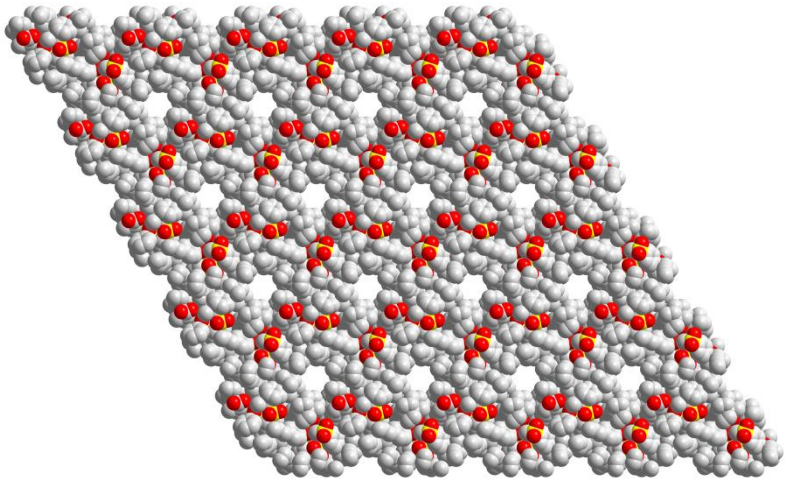

Crystal packing structure of **6g-ester** (5x5 unit cells) showing the void channels.

Esterification of boronic acid 1b with EtOH

We stated in the main text that „ ... the enantioselective version of the reaction starts with mono- or diesterification of allenyl boronic acid **1b** with EtOH.“ In Figure 2 below we present experimental data supporting this statement.

The ^{1}H NMR spectrum of **1b** toluene-d_8 (Figure 2i) shows a characteristic peak “**a**“ (4.16 ppm), which belongs to the free (unesterified) $B(O\underline{H})_2$ group. The sample was obtained by extraction (see preparation above) and therefore it contains (non-deuterated) toluene. One hour after reacting 0.1 mmol of **1b** with 0.2 mmol of EtOH in the presence of MS (3 Å) in degassed toluene, the ^{1}H NMR spectrum of this reaction mixture was monitored by ^{1}H NMR (Figure 2ii). In spectrum ii) the peak “**a**“ of the free $B(OH)_2$ group at 4.16 ppm does not appear any more indicating the esterification of the $B(OH)_2$ group, i.e. formation of B(OH)(OEt) or $B(OEt)_2$. In Figure 2ii the quartett(s) at 4.00 ppm probably belongs to the ethylester of the allenylboronic acid, while traces of the CH_2 group from free EtOH gives a weak multiplett (dq) at 3.29 ppm.

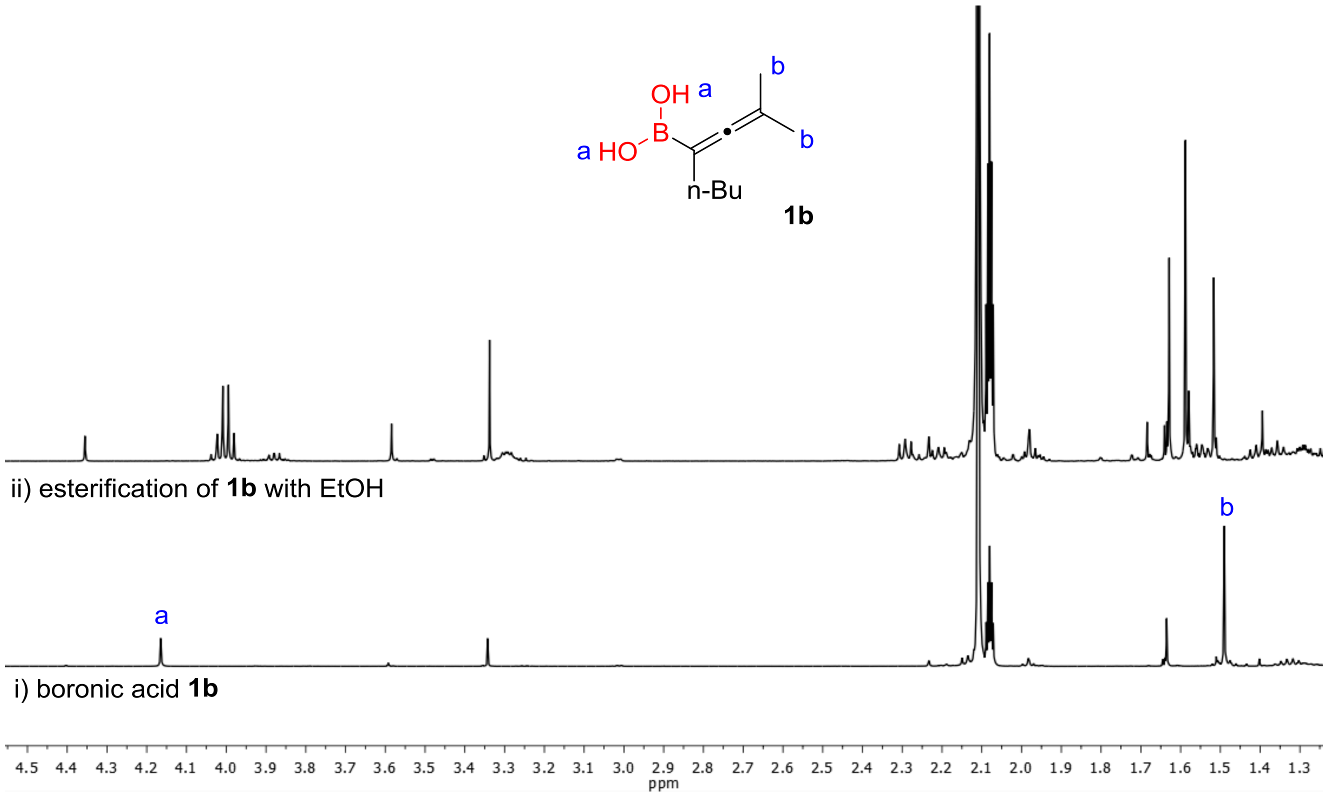

Figure 2. ^{1}H NMR supporting the esterification of boronic acid **1b** with EtOH (500 MHz, tol-d_8).

References:

1) H. Ito, Y. Sasaki, M. Sawamura, *J. Am. Chem. Soc.* **2008,** *130*, 15774.

2) T. S. Zhao,Y. Yang, T. Lessing, K. J. Szabo, *J. Am. Chem. Soc.* **2014,** *136*, 7563.

3) J. Sun, M. T. Perfetti, W. L. Santos, *J. Org. Chem.* **2011,** *76*, 3571.

4) G. A. Molander, S. L. J. Trice, S. D. Dreher, *J. Am. Chem. Soc.* **2010,** *132*, 17701.

5) S. Hayashi, K. Hirano, H. Yorimitsu, K. Oshima, *J. Am. Chem. Soc.* **2008,** *130*, 5048.

Spectra for **1a:** ^{1}H NMR (400 MHz, tol-d_8):

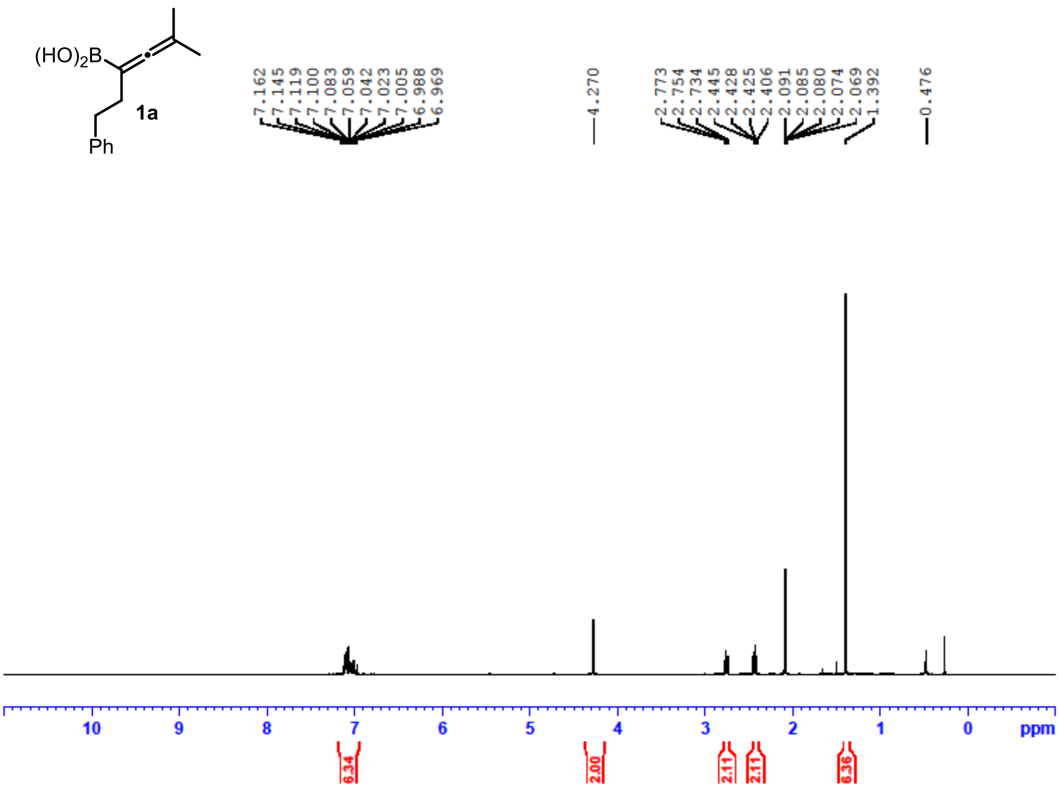

^{13}C NMR (100 MHz, tol-d_8)

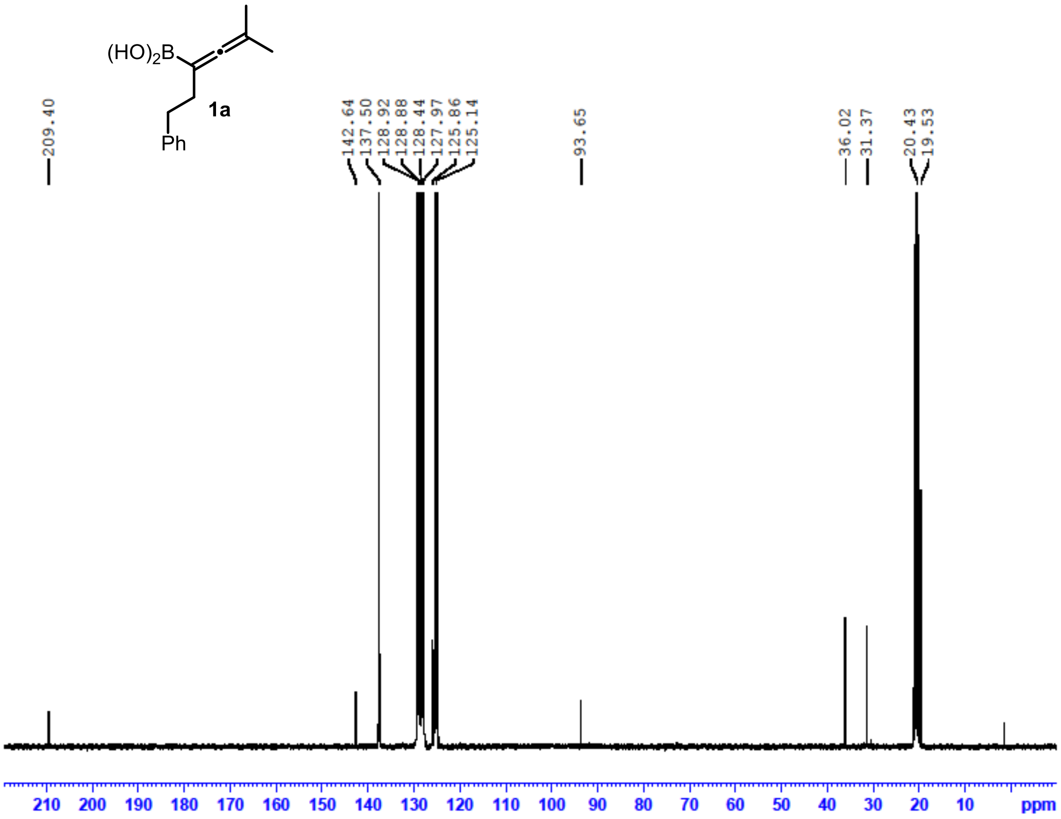

^{11}B NMR (128 MHz, tol-d_8):

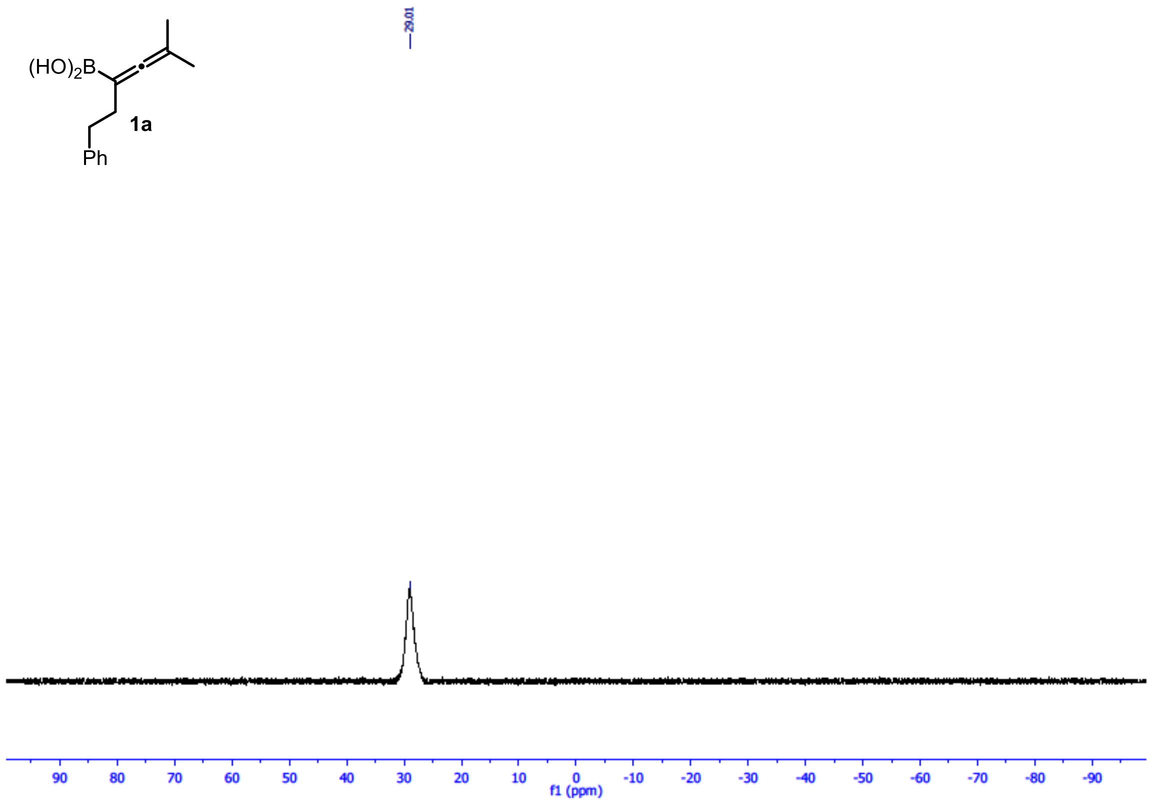

Spectra for **1b:** ^{1}H NMR (500 MHz, tol-d_8):

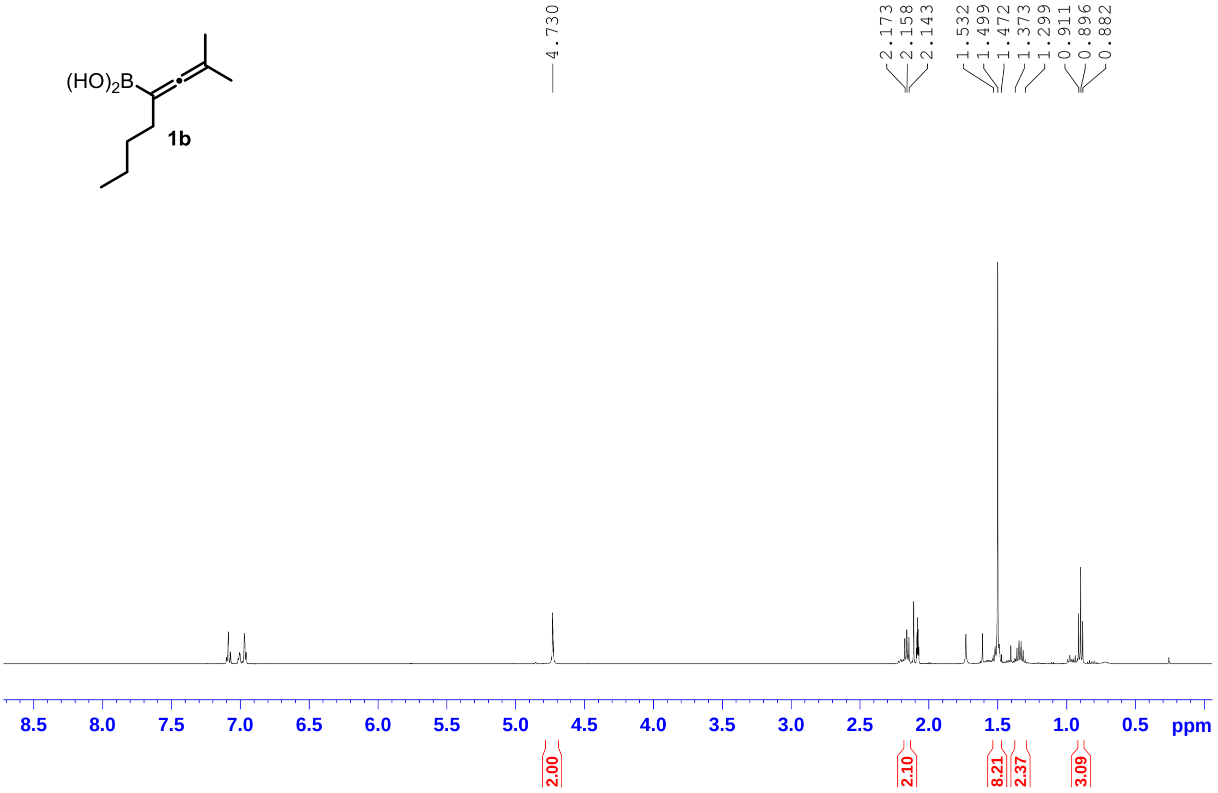

^{13}C NMR (125 MHz, tol-d$_8$)

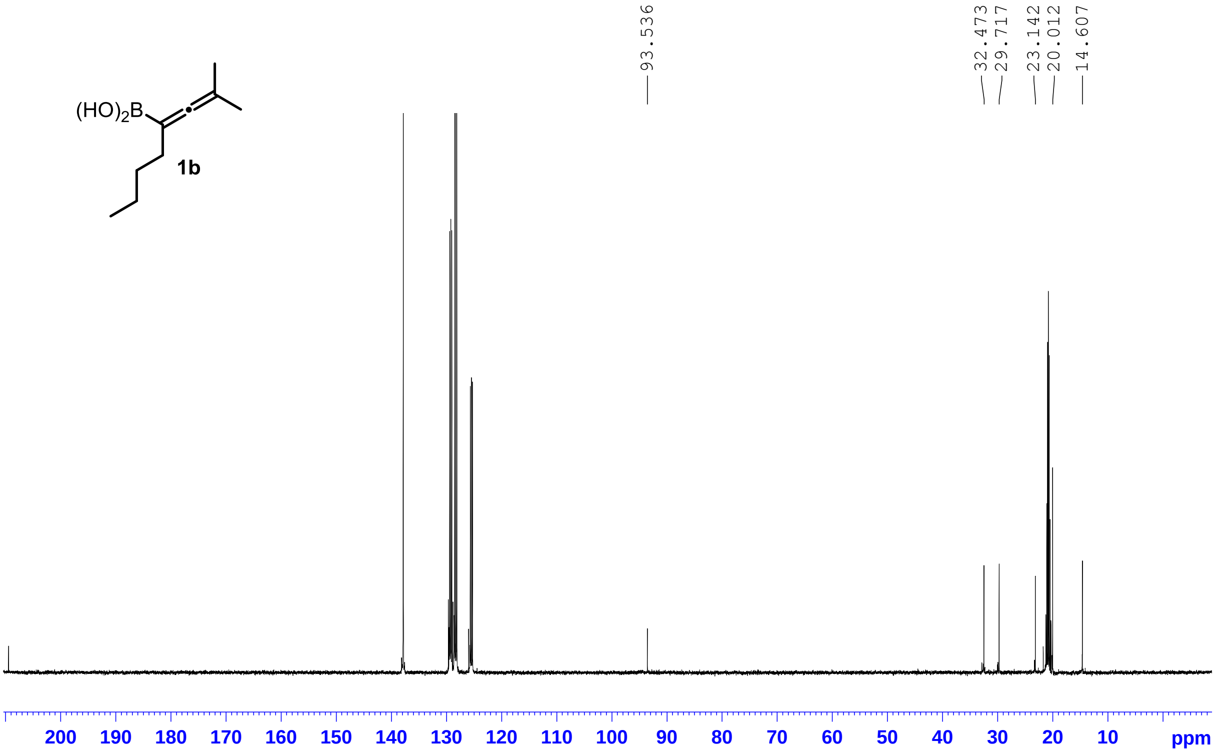

^{11}B NMR (128 MHz, tol-d$_8$):

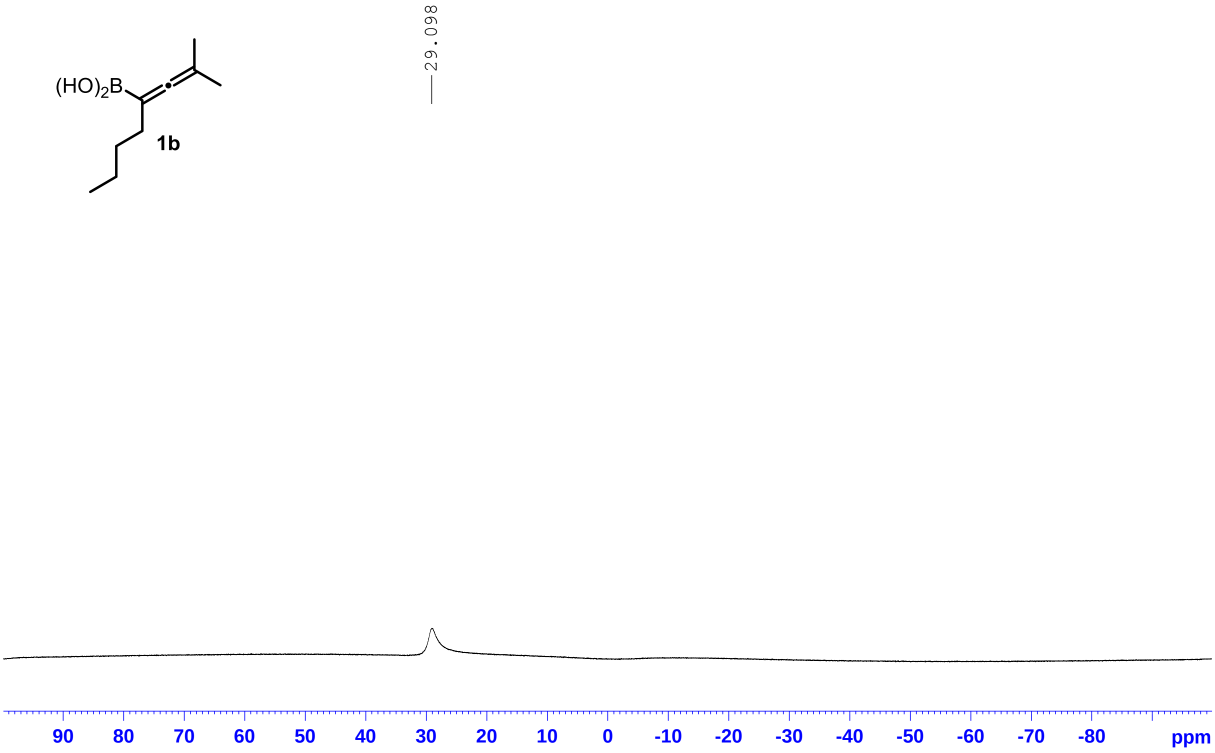

Spectrum for **1c:** ^{1}H NMR (500 MHz, tol-d_8). The sample was obtained by extraction of the crude mixture with tol-d_8. Naphtalene was used as internal standard.

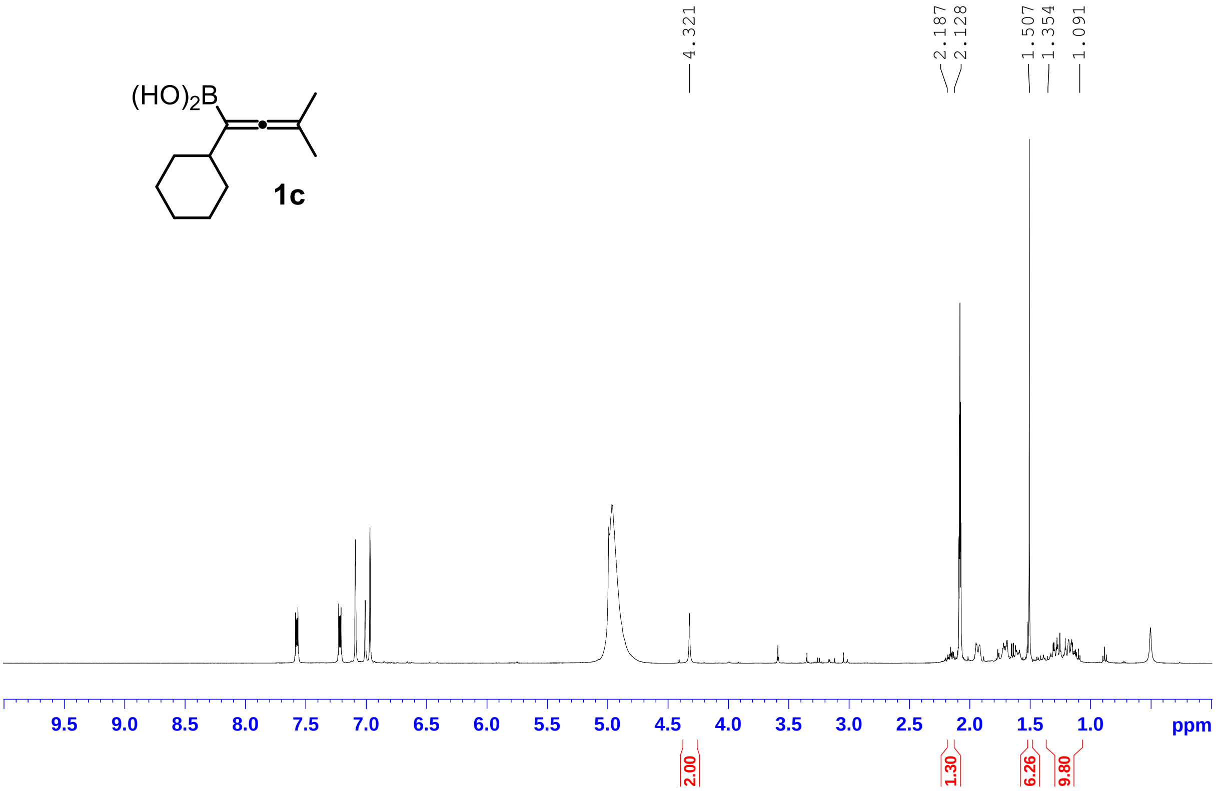

Spectrum for **1d:** ^{1}H NMR (500 MHz, tol-d_8). The sample was obtained by extraction of the crude mixture with tol-d_8. Naphtalene was used as internal standard.

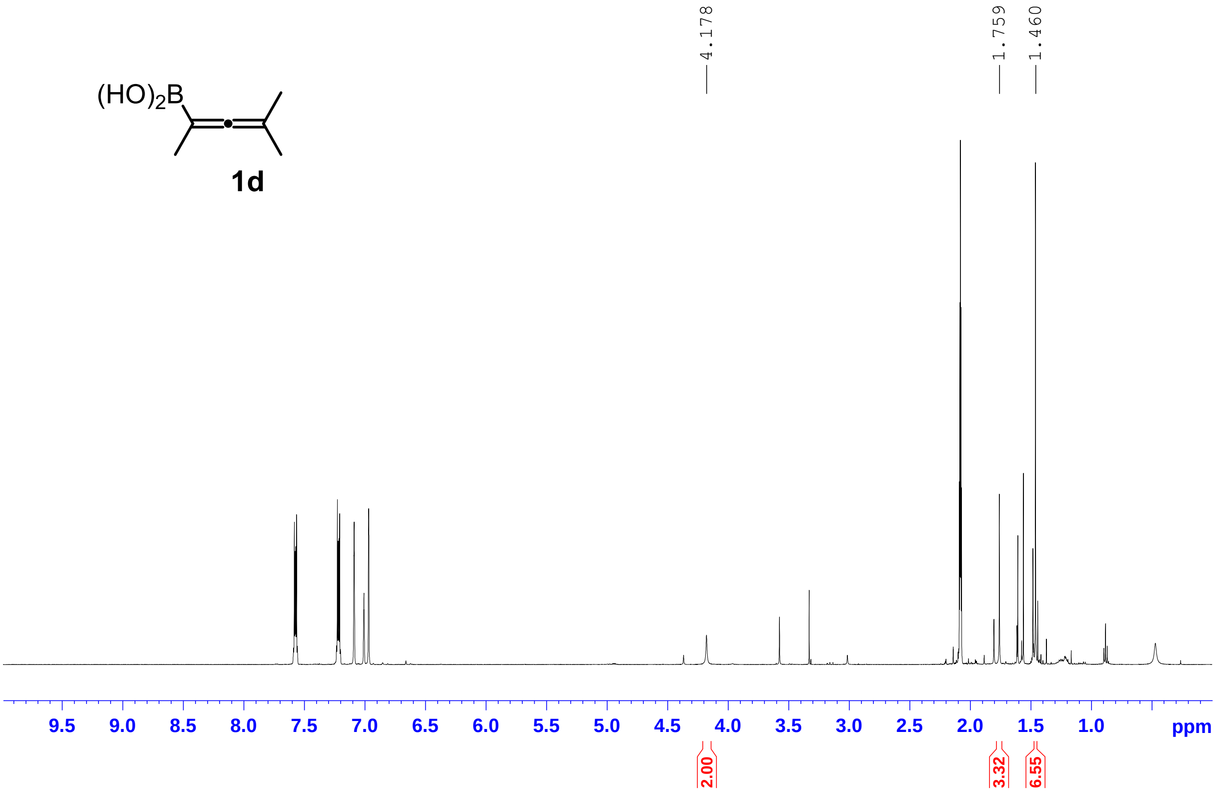

Spectrum for **1e:** ^{1}H NMR (500 MHz, tol-d_8). The sample was obtained by extraction of the crude mixture with tol-d_8. Naphtalene was used as internal standard.

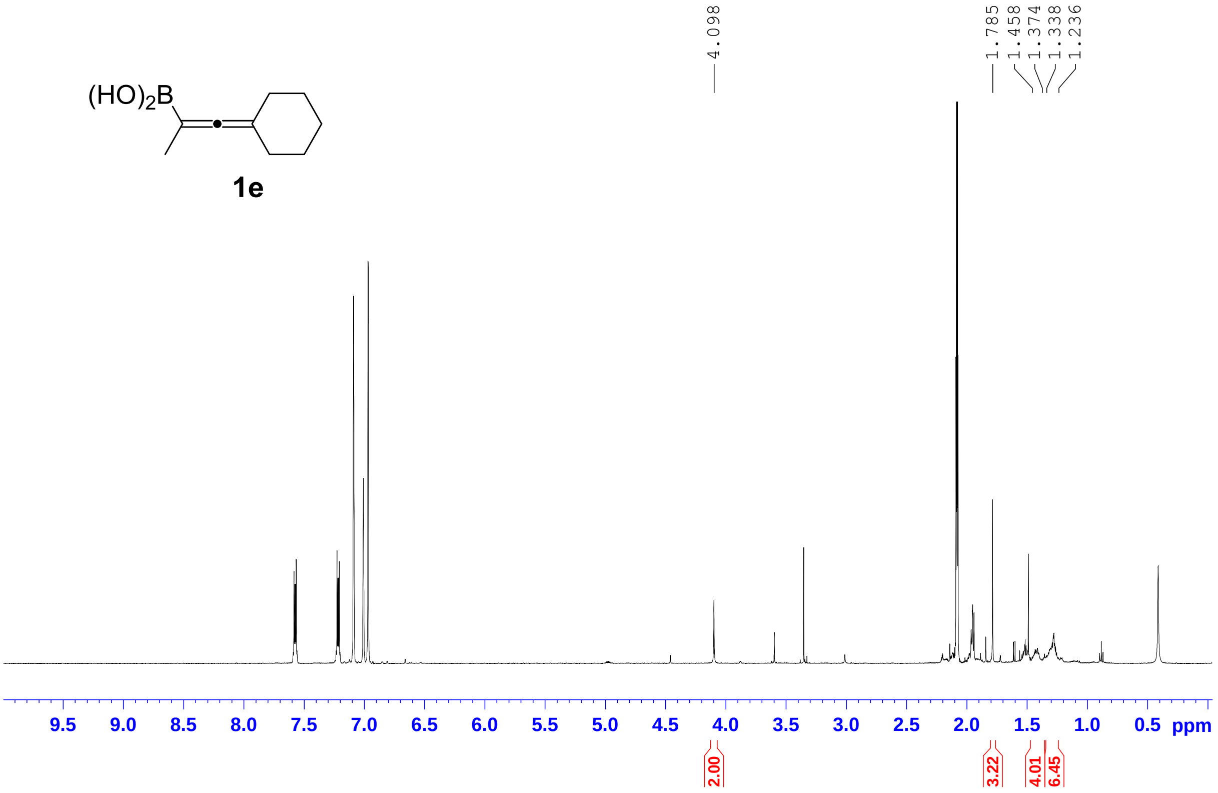

Spectrum for **1f:** ^{1}H NMR (500 MHz, tol-d_8). The sample was obtained by extraction of the crude mixture with tol-d_8. Naphtalene was used as internal standard.

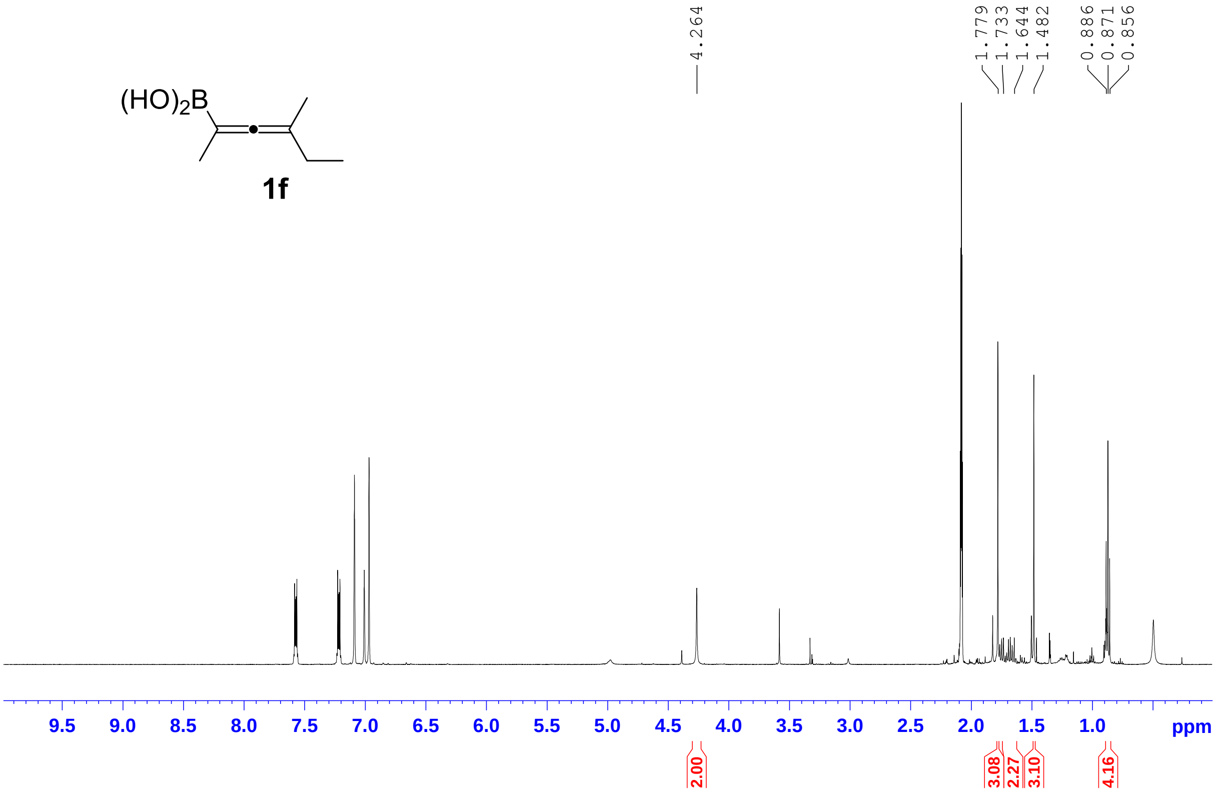

Spectrum for **1g:** ^{1}H NMR (500 MHz, tol-d_8). The sample was obtained by extraction of the crude mixture with tol-d_8. Naphtalene was used as internal standard.

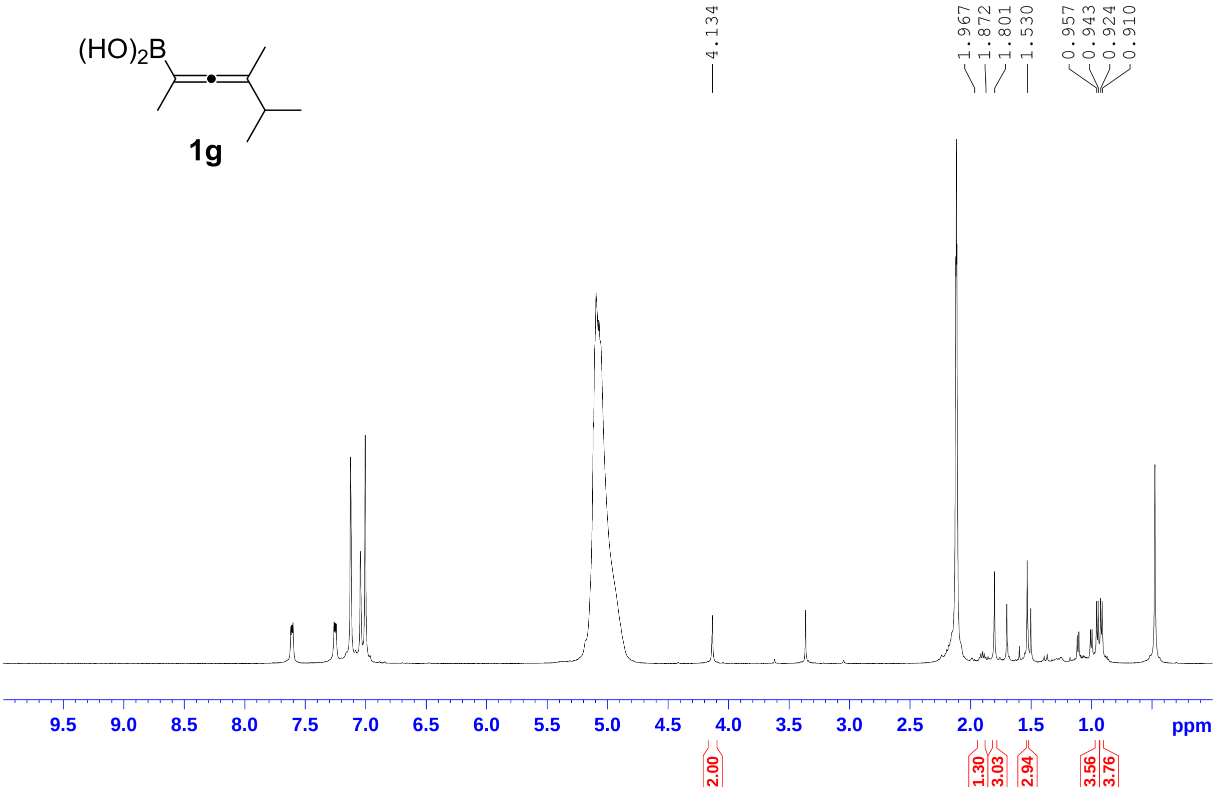

Spectrum for **1h:** ^{1}H NMR (500 MHz, tol-d_8). The sample was obtained by extraction of the crude mixture with tol-d_8. Naphtalene was used as internal standard.

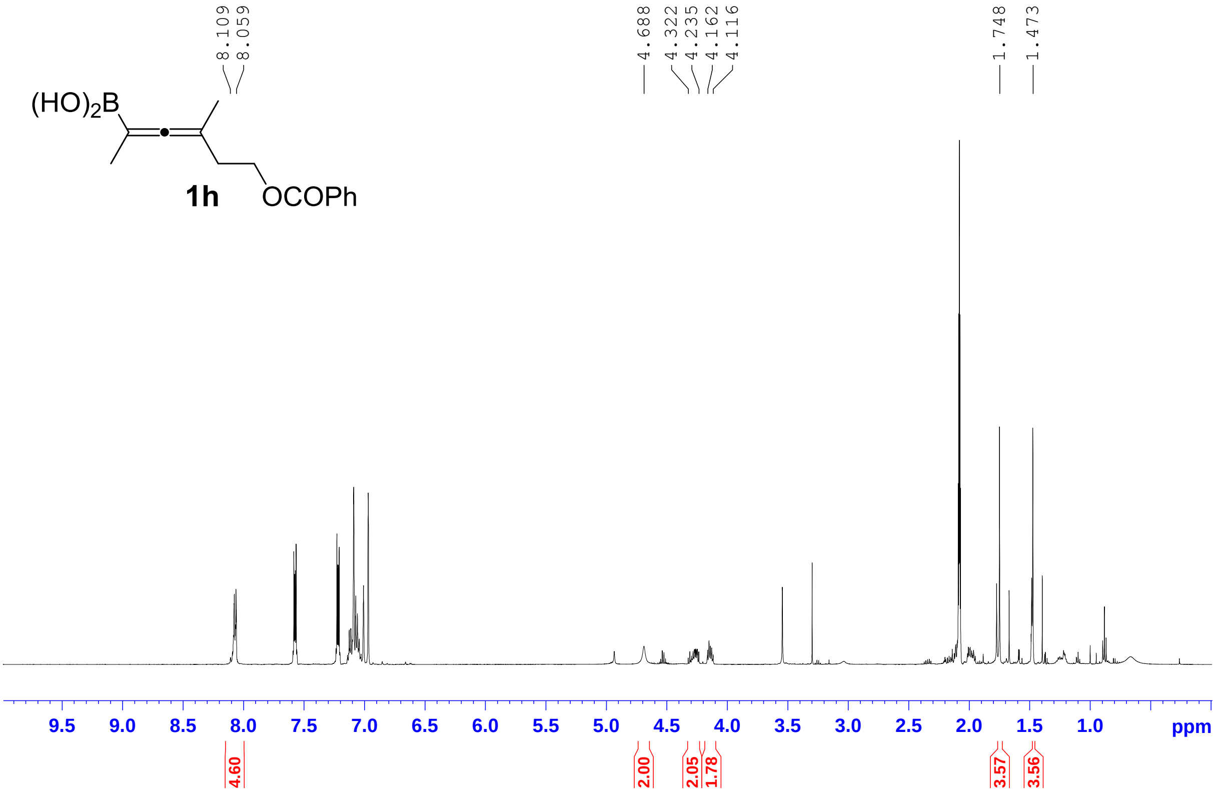

Spectrum for **1i:** ^{1}H NMR (500 MHz, tol-d$_8$). The sample was obtained by extraction of the crude mixture with tol-d$_8$. Naphtalene was used as internal standard.

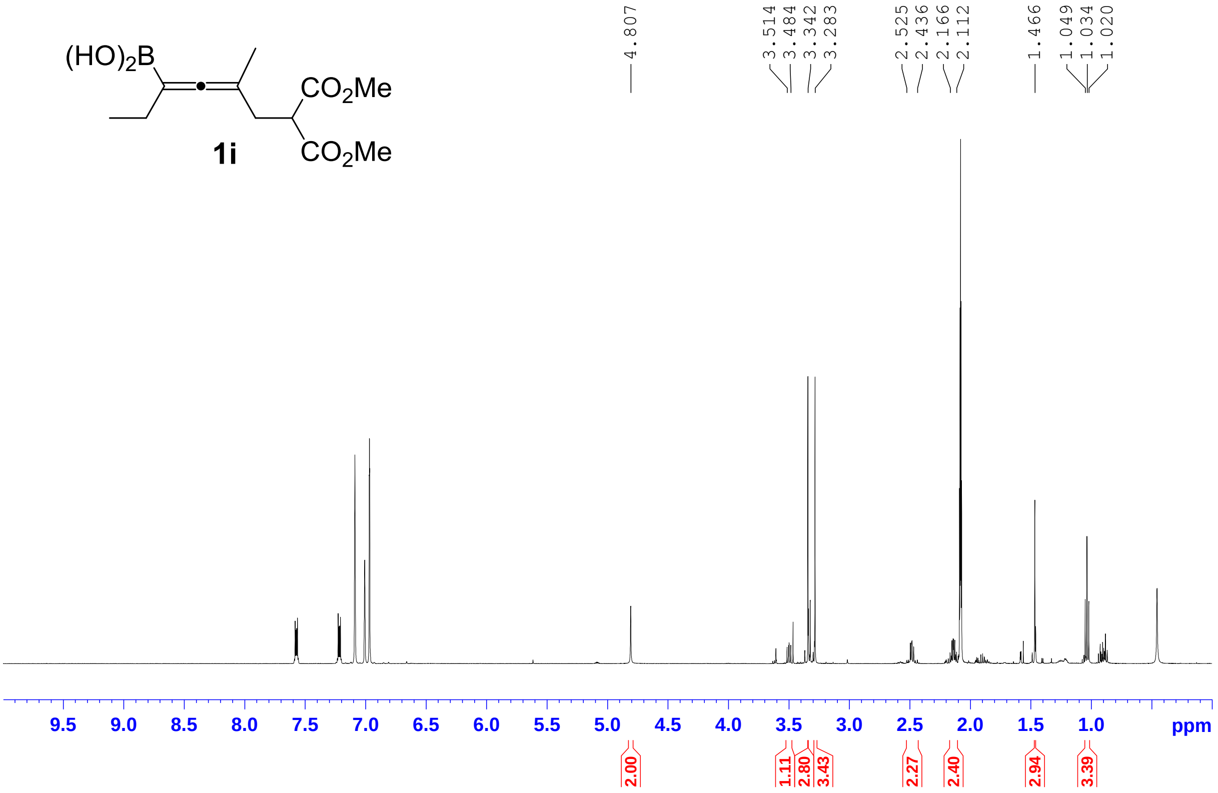

Spectrum for **1j:** ^{1}H NMR (500 MHz, tol-d$_8$). The sample was obtained by extraction of the crude mixture with tol-d$_8$. Naphtalene was used as internal standard.

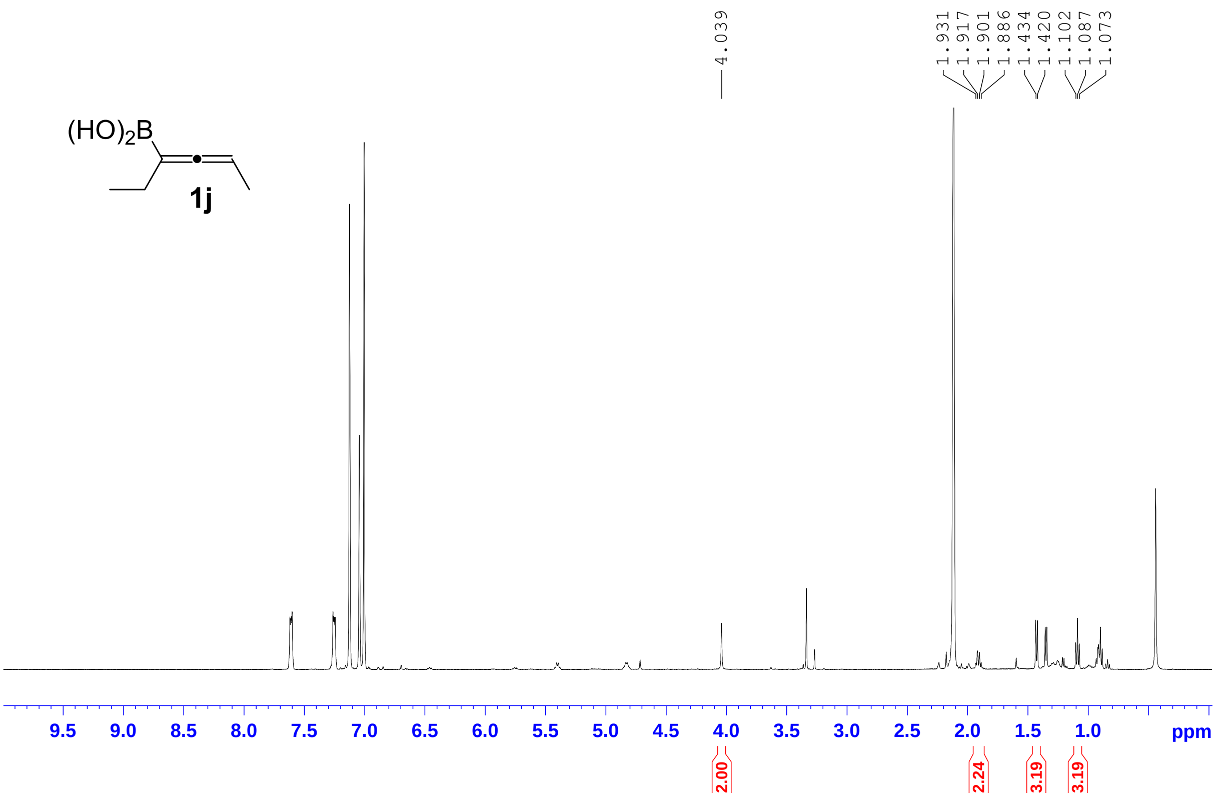

Spectra for **1a-Bpin:** ^{1}H NMR (400 MHz, C_6D_6):

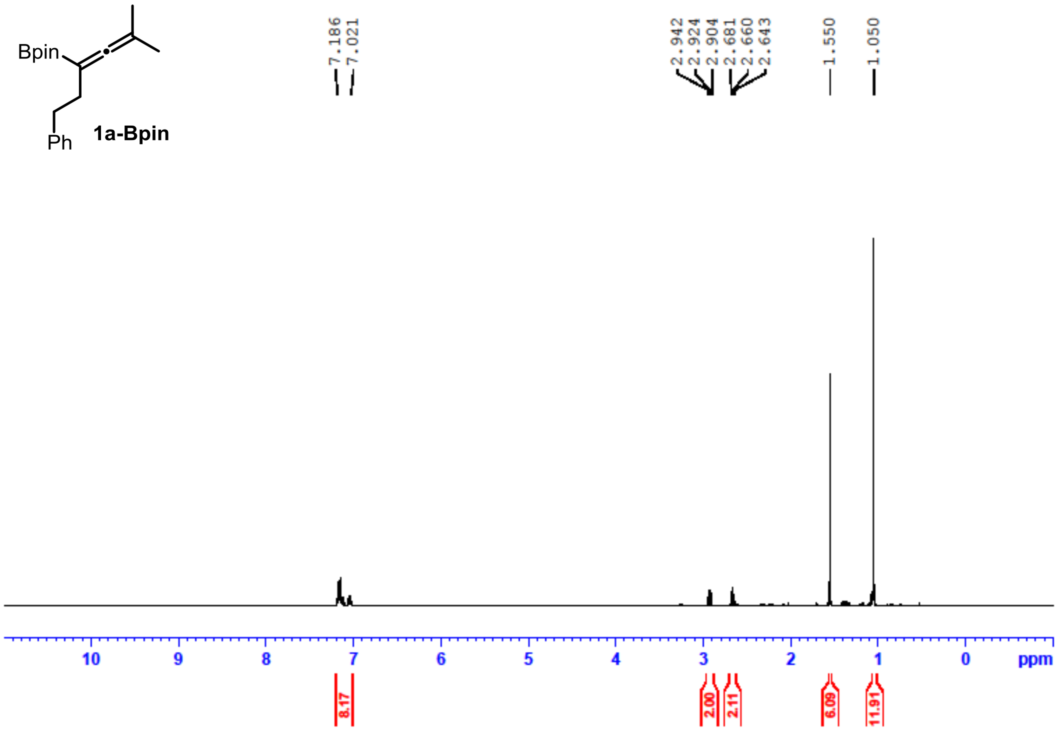

^{13}C NMR (100 MHz, C_6D_6):

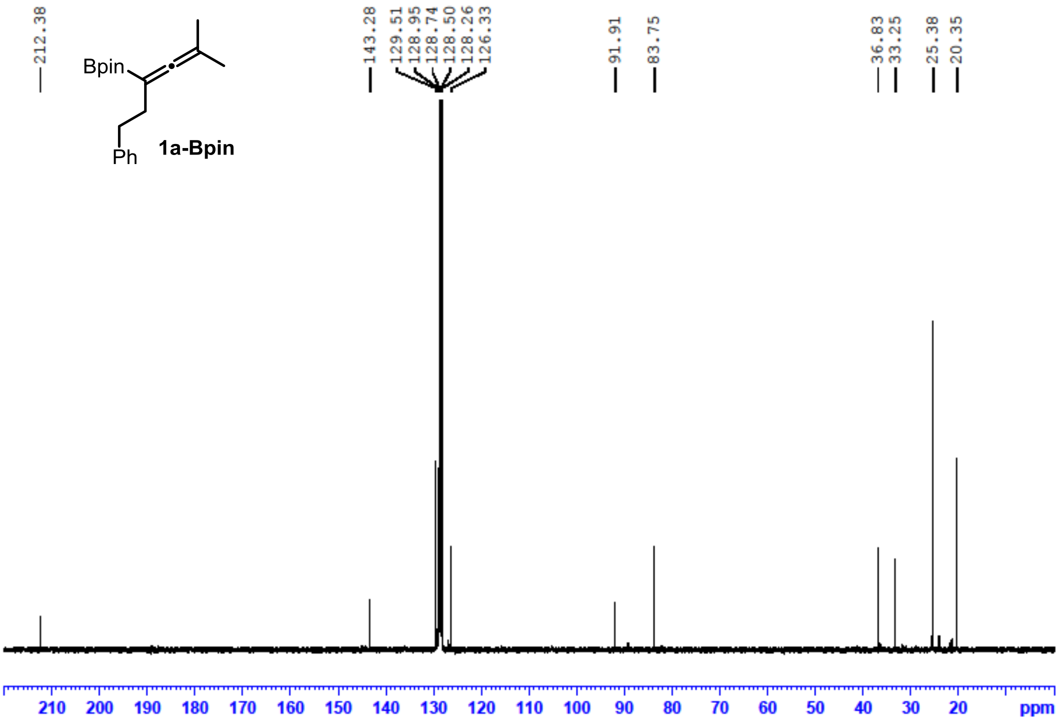

^{11}B NMR (128 MHz, C_6D_6):

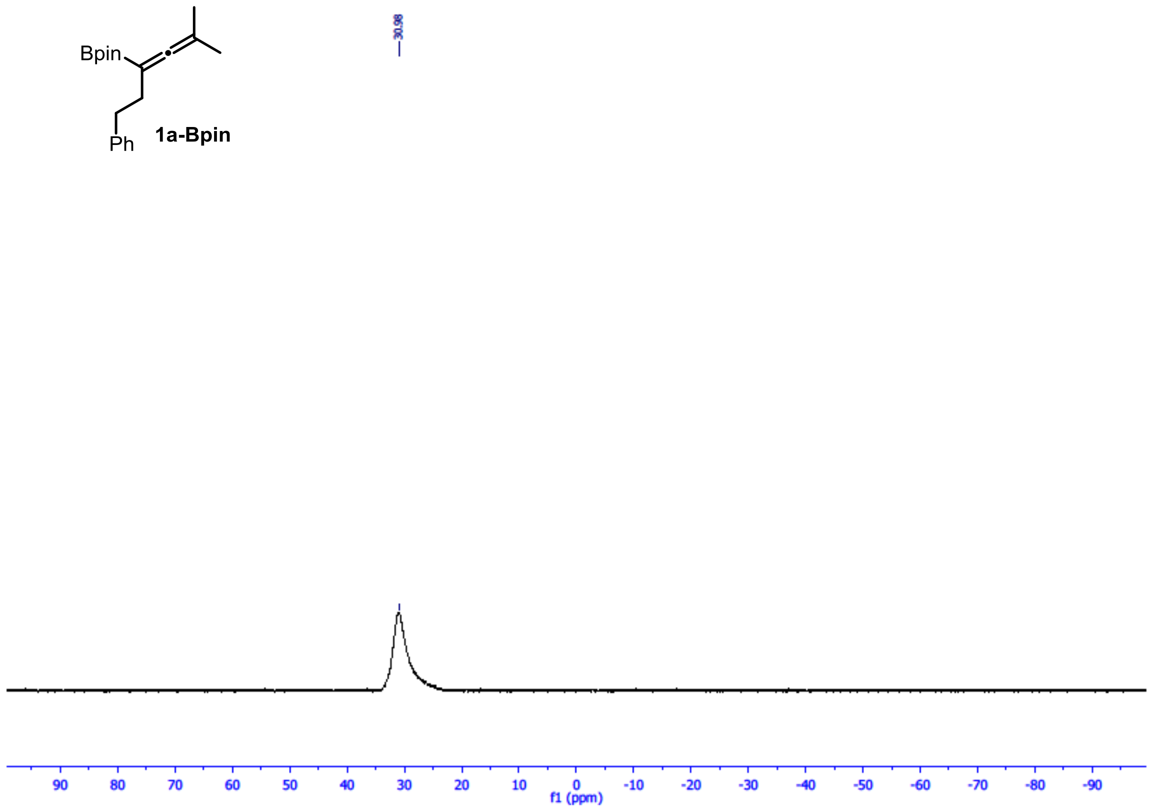

Spectra for **1a-Bpne:** ^{1}H NMR (400 MHz, $CDCl_3$):

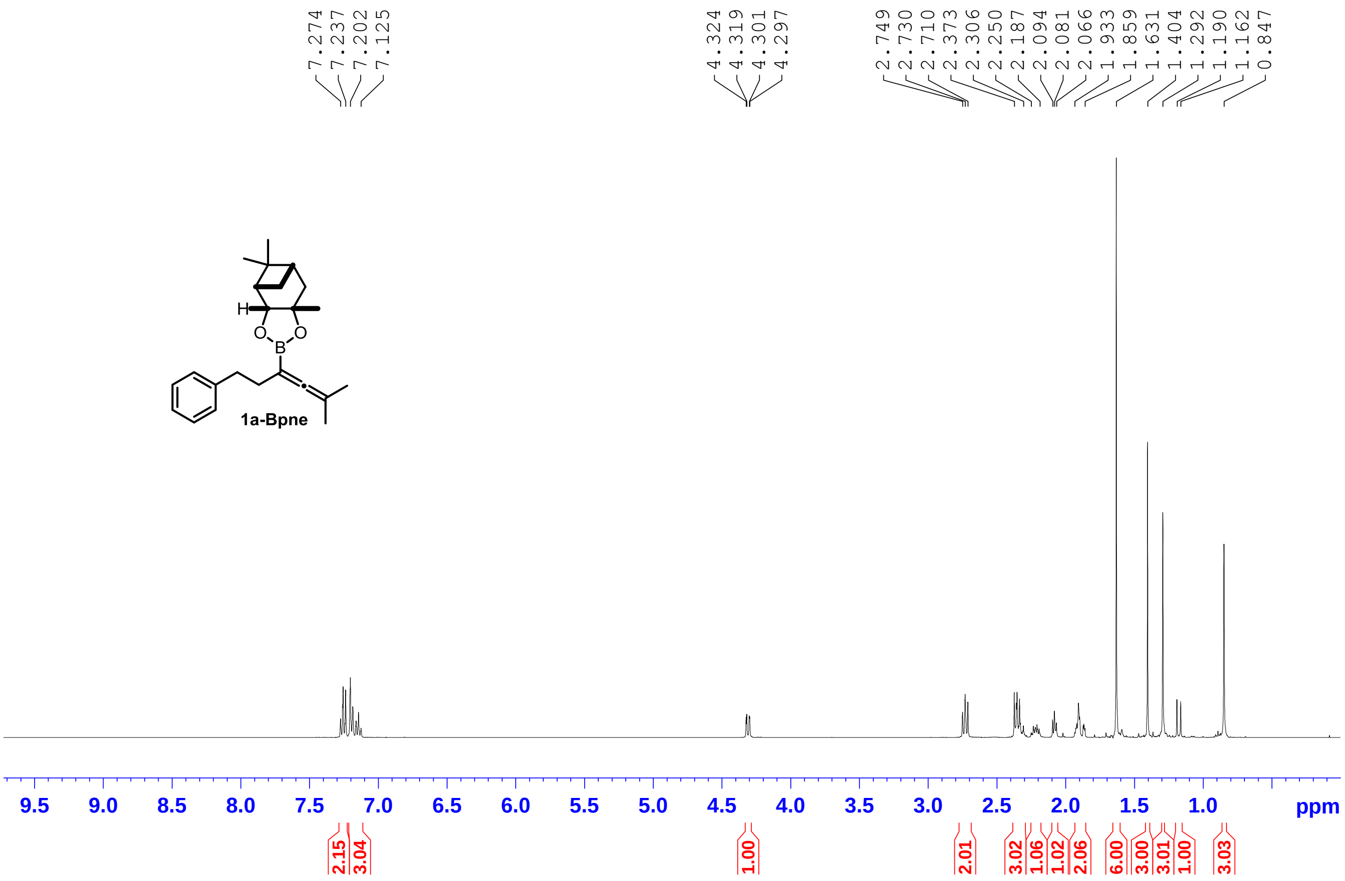

^{13}C NMR (100 MHz, $CDCl_3$)

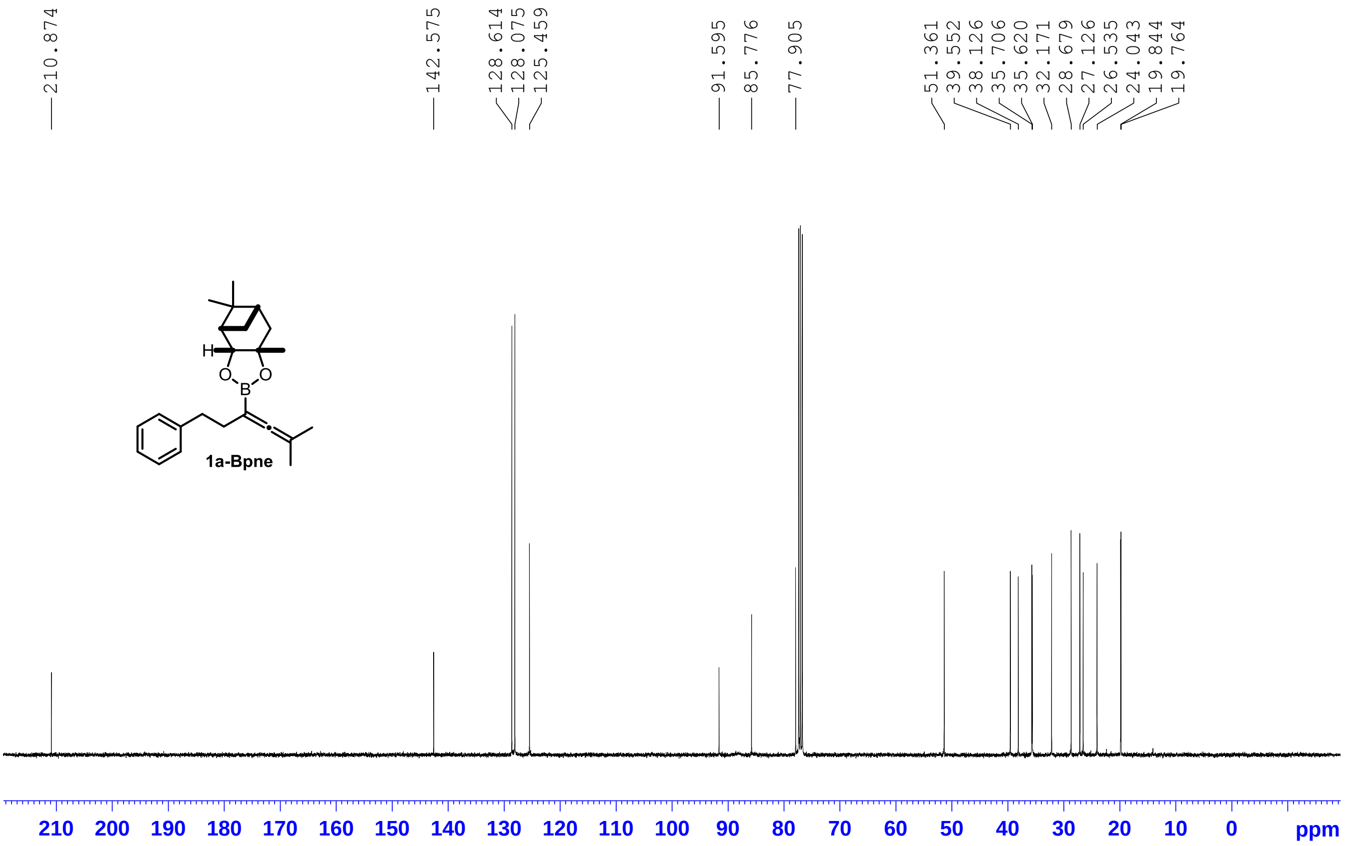

^{11}B NMR (128 MHz, $CDCl_3$)

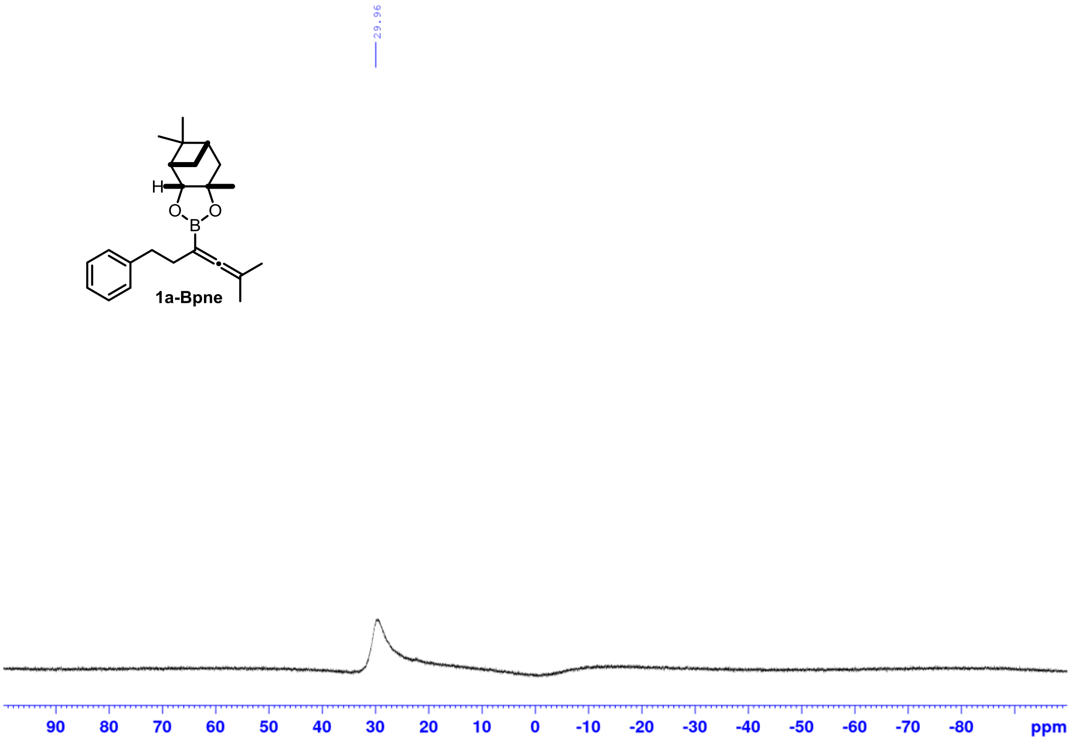

Spectrum of toluene extract of **1a-Bnep:** ^{1}H NMR (400 MHz, $CDCl_3$):

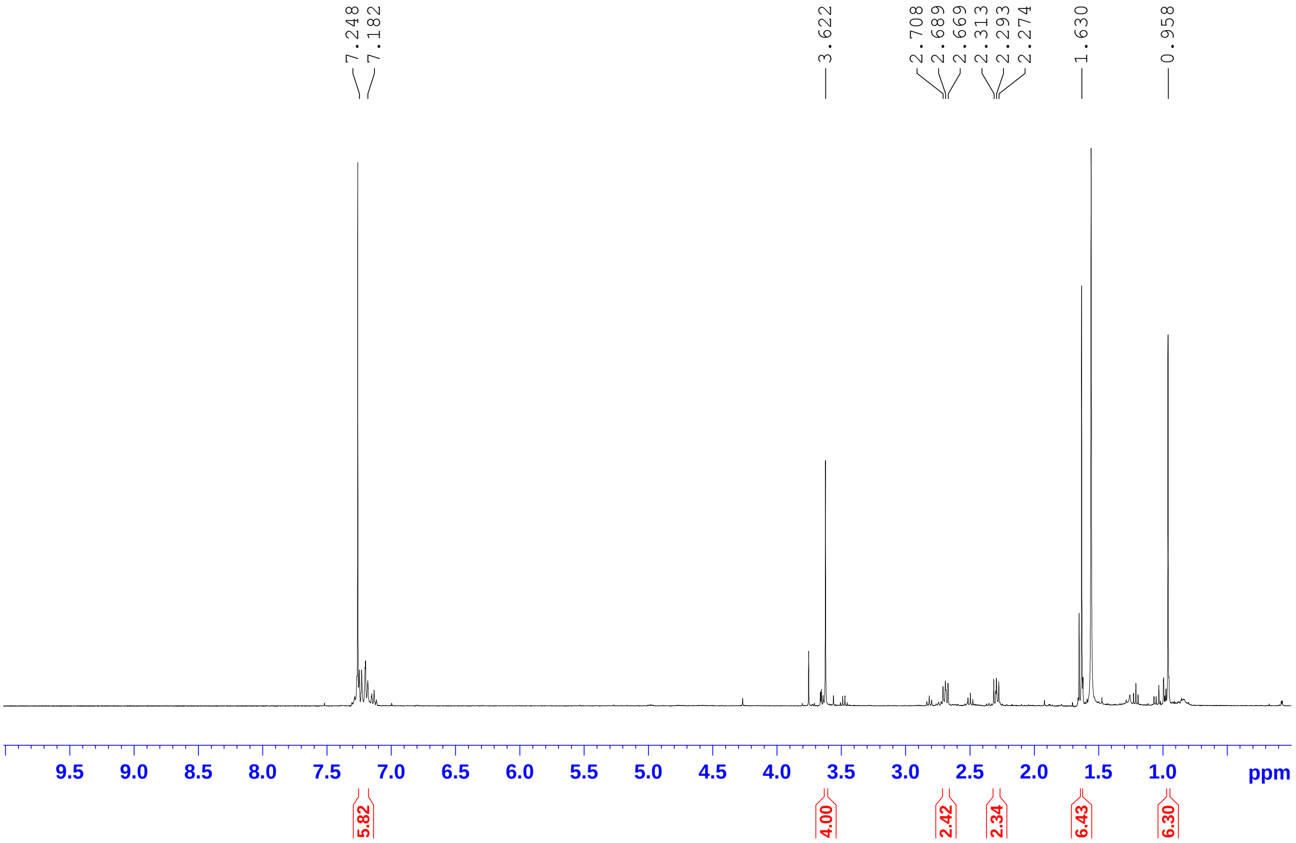

Spectra for **6a:** ^{1}H NMR (400 MHz, $CDCl_3$):

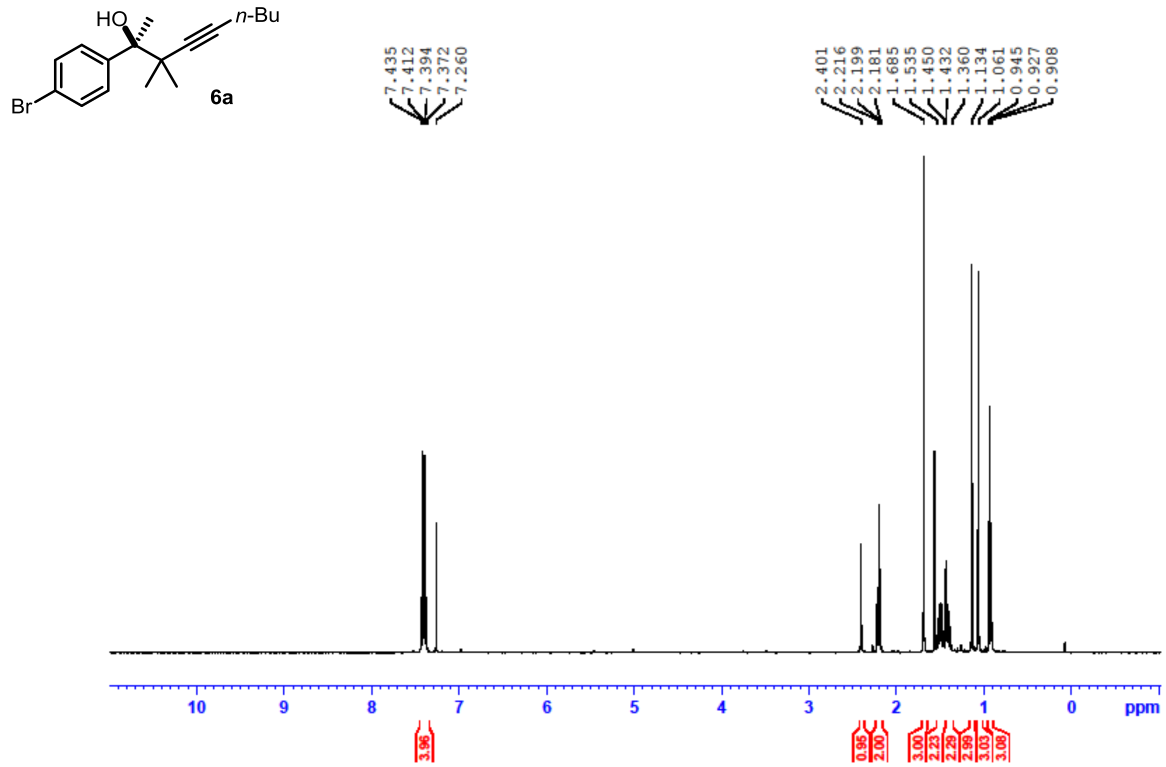

^{13}C NMR (100 MHz, $CDCl_3$):

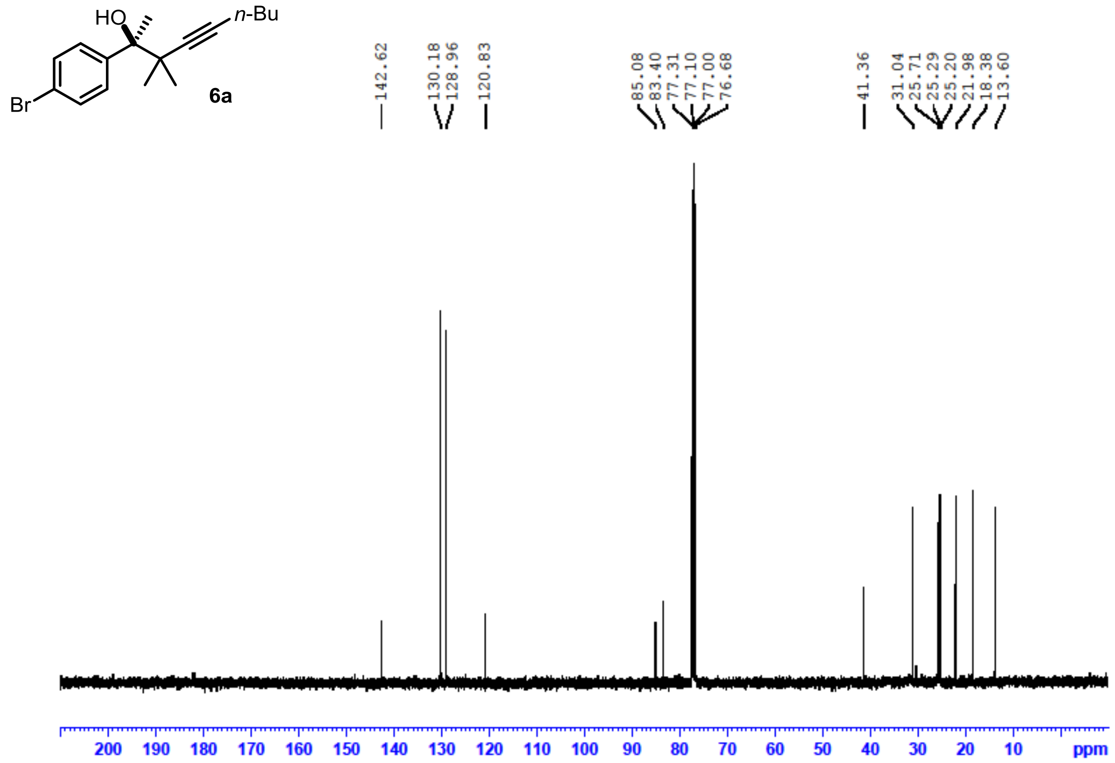

Spectra for **6b:** ^{1}H NMR (400 MHz, $CDCl_3$):

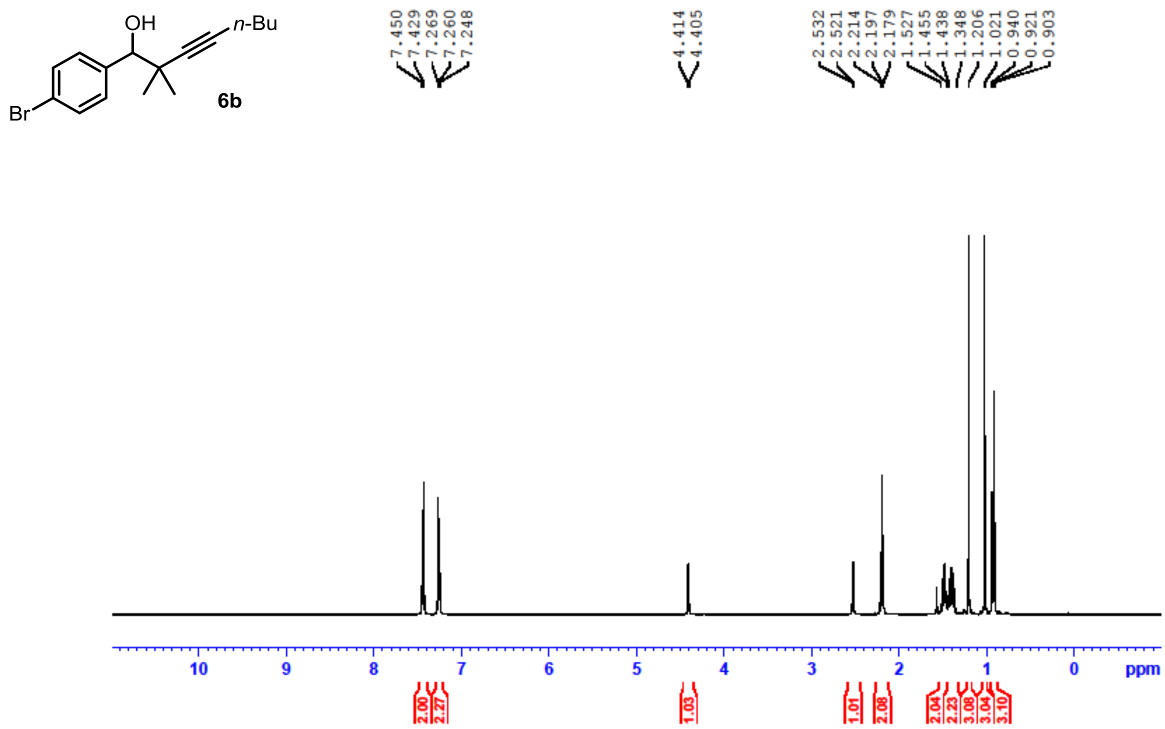

^{13}C NMR (100 MHz, $CDCl_3$):

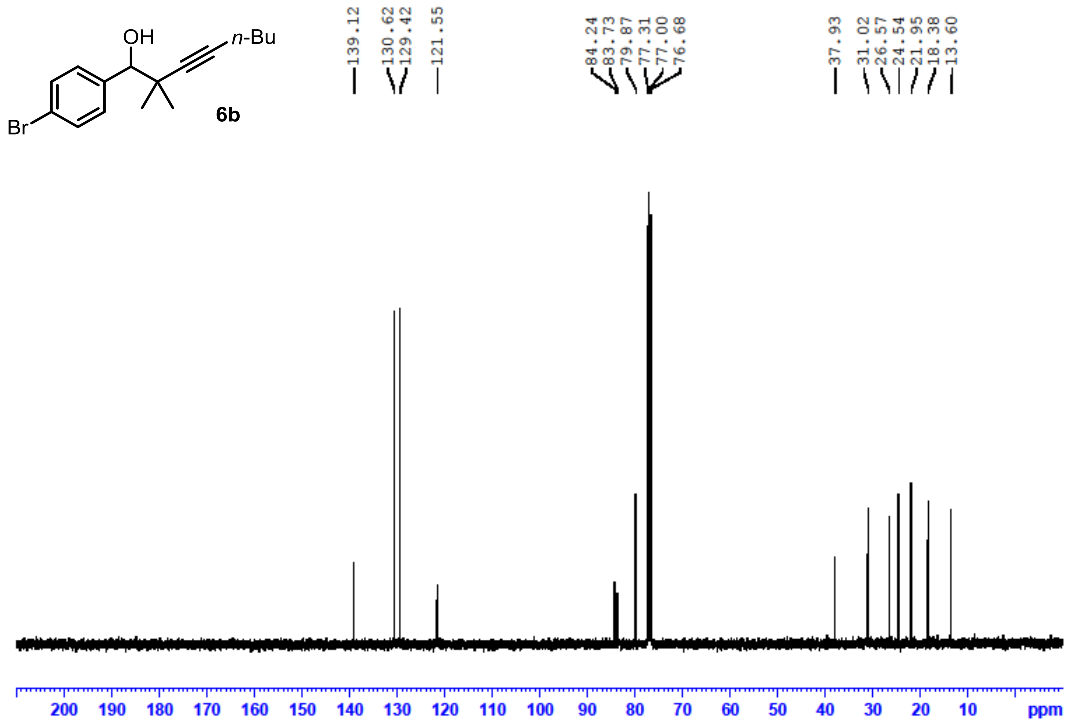

Spectra for **6c:** ^{1}H NMR (400 MHz, $CDCl_3$):

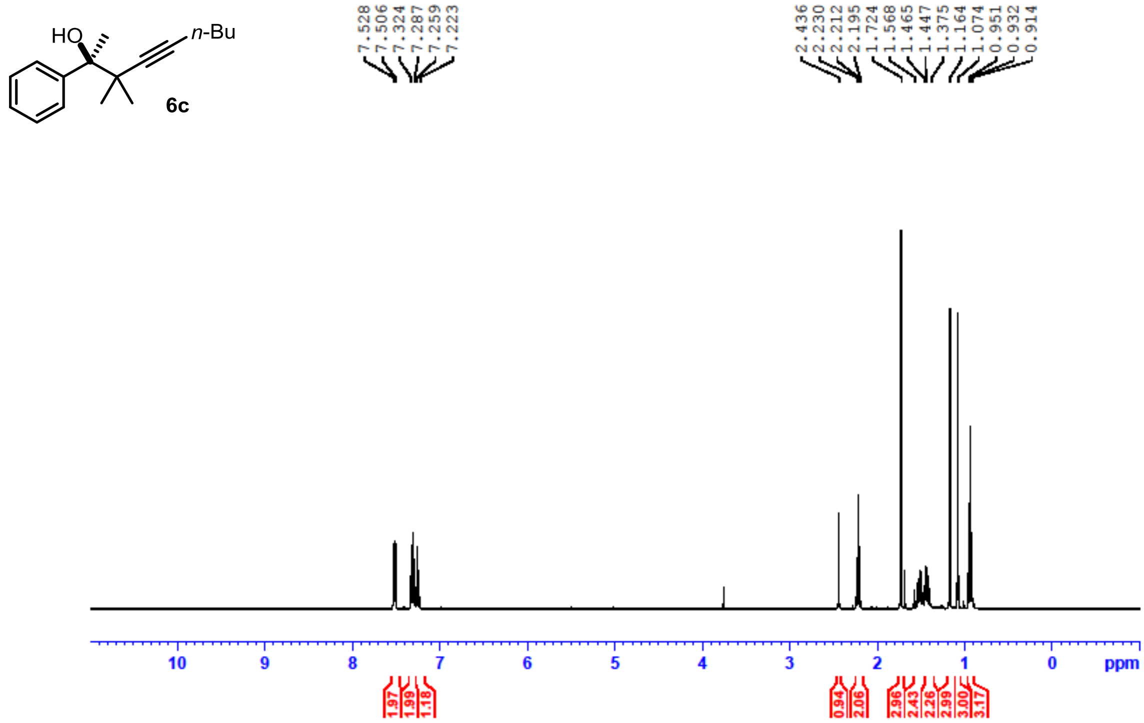

^{13}C NMR (100 MHz, $CDCl_3$):

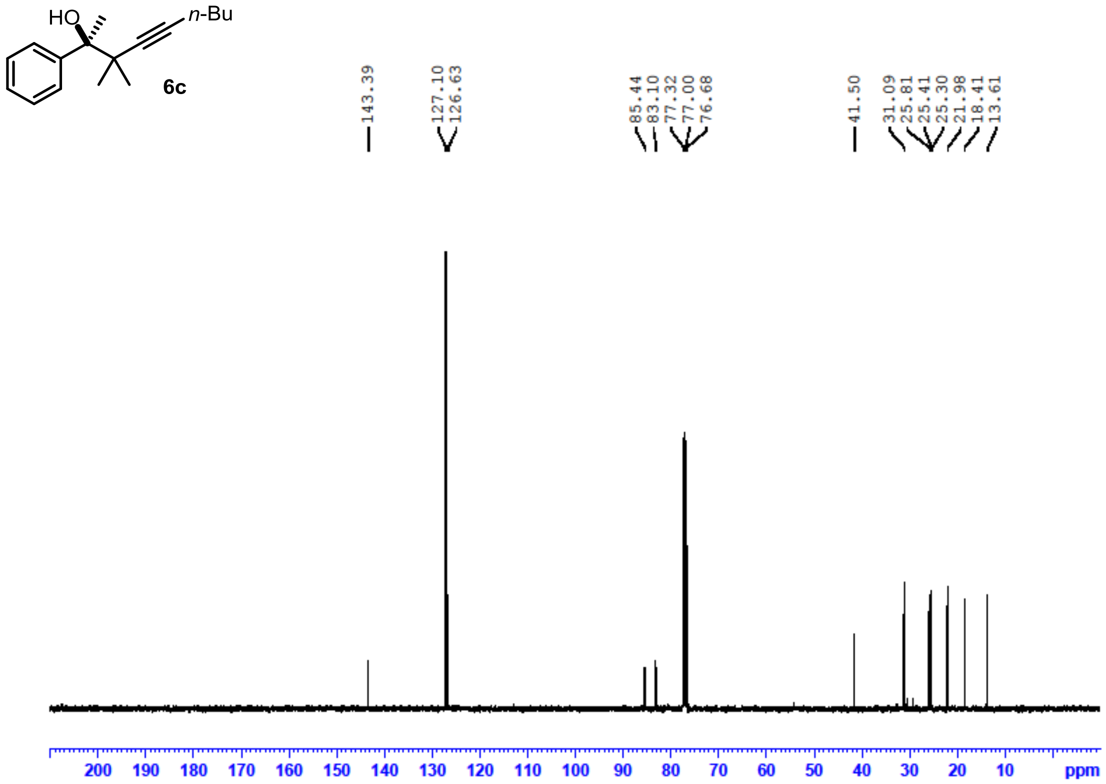

Spectra for **6d:** ^{1}H NMR (400 MHz, $CDCl_3$):

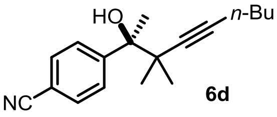

6d

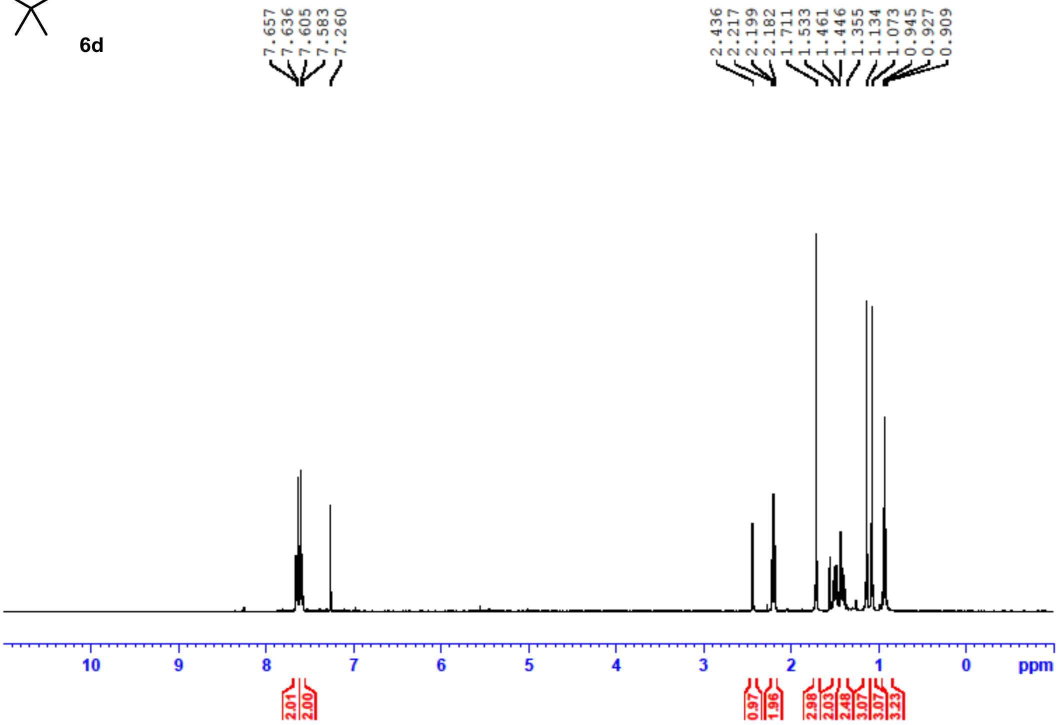

^{13}C NMR (100 MHz, $CDCl_3$):

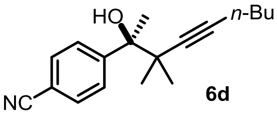

6d

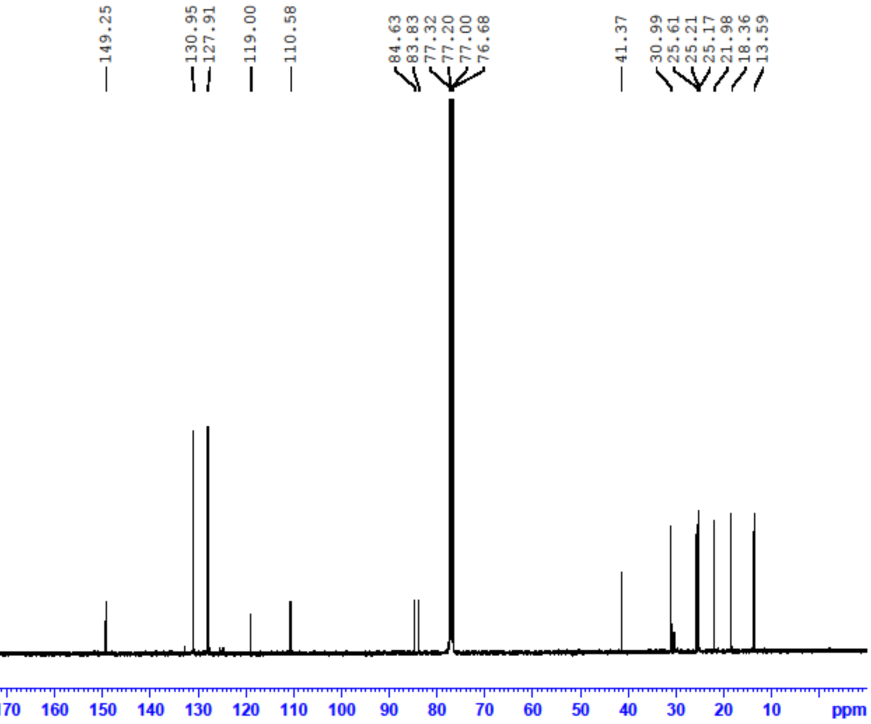

Spectra for **6e:** ^{1}H NMR (400 MHz, $CDCl_3$):

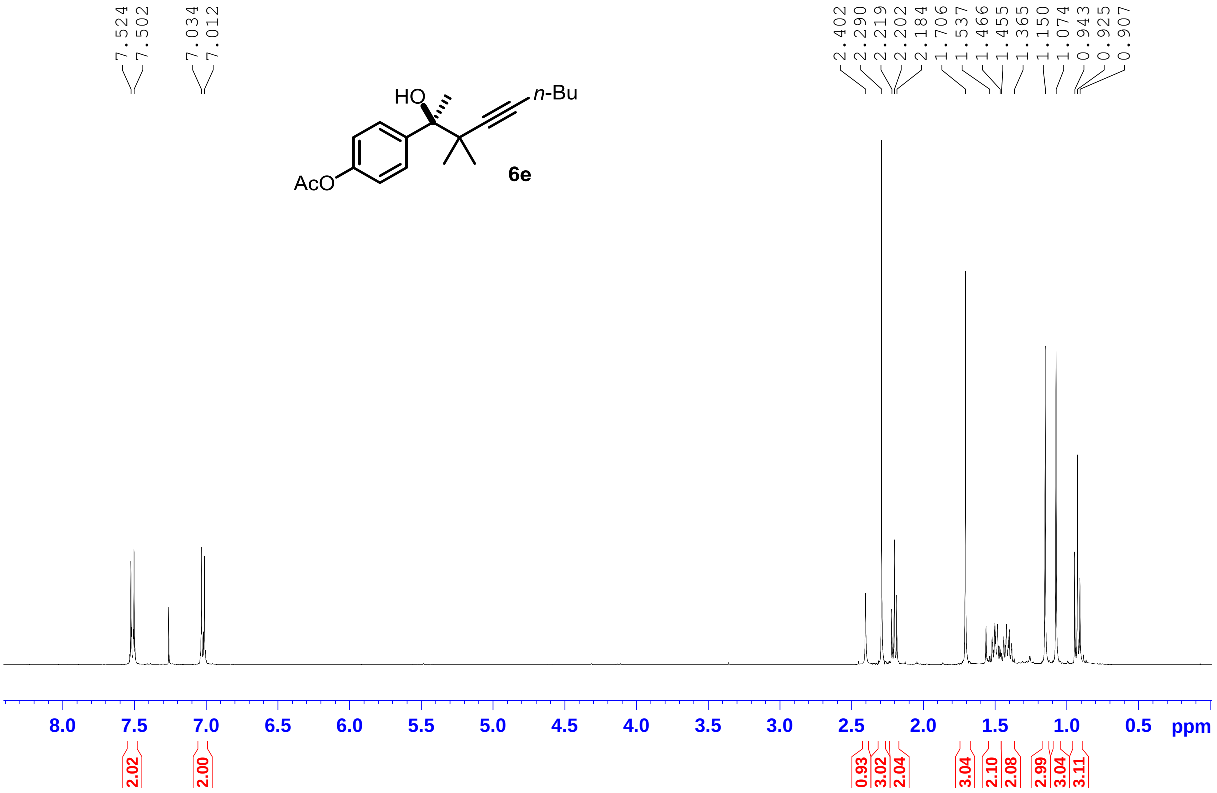

^{13}C NMR (100 MHz, $CDCl_3$):

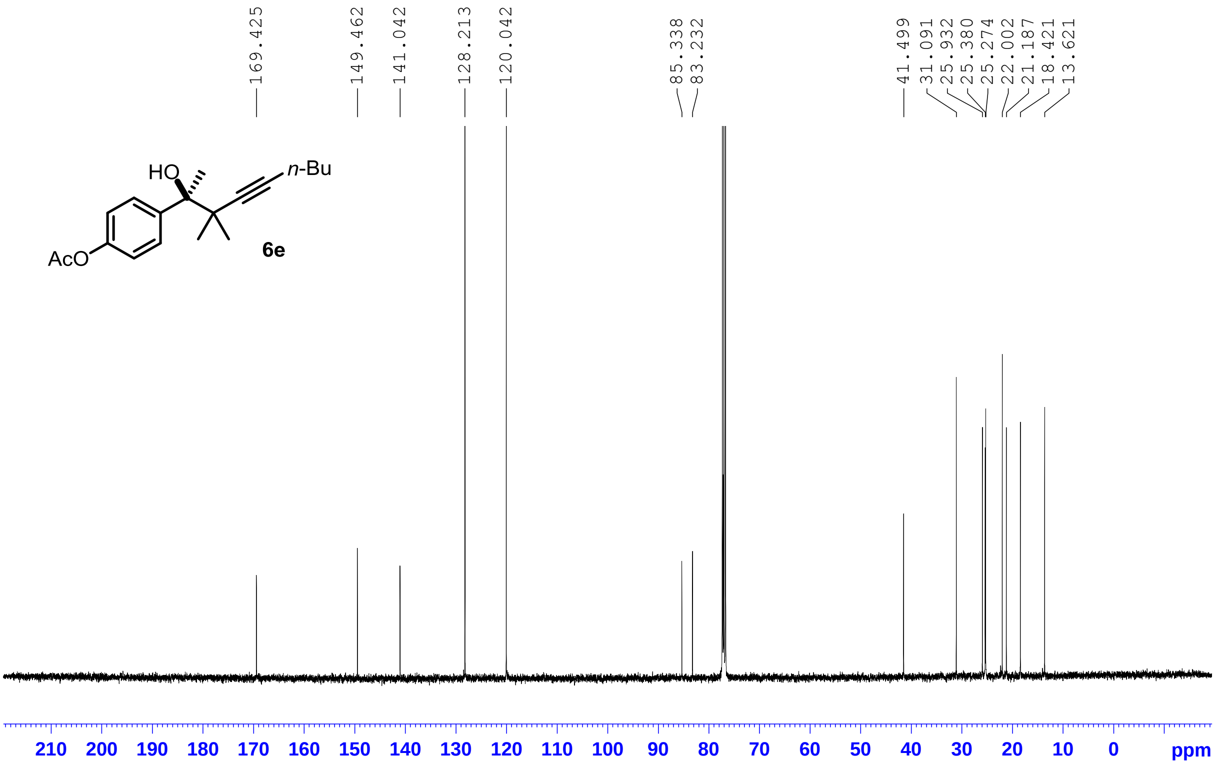

Spectra for **6f:** ^{1}H NMR (400 MHz, $CDCl_3$):

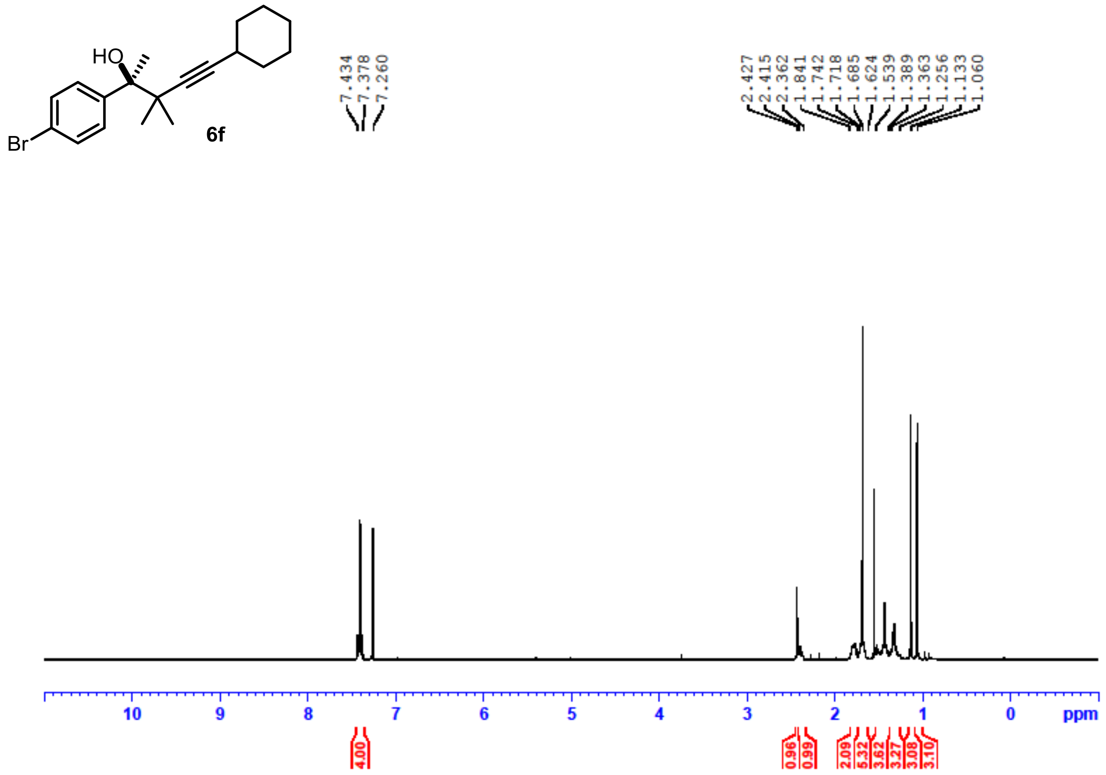

^{13}C NMR (100 MHz, $CDCl_3$):

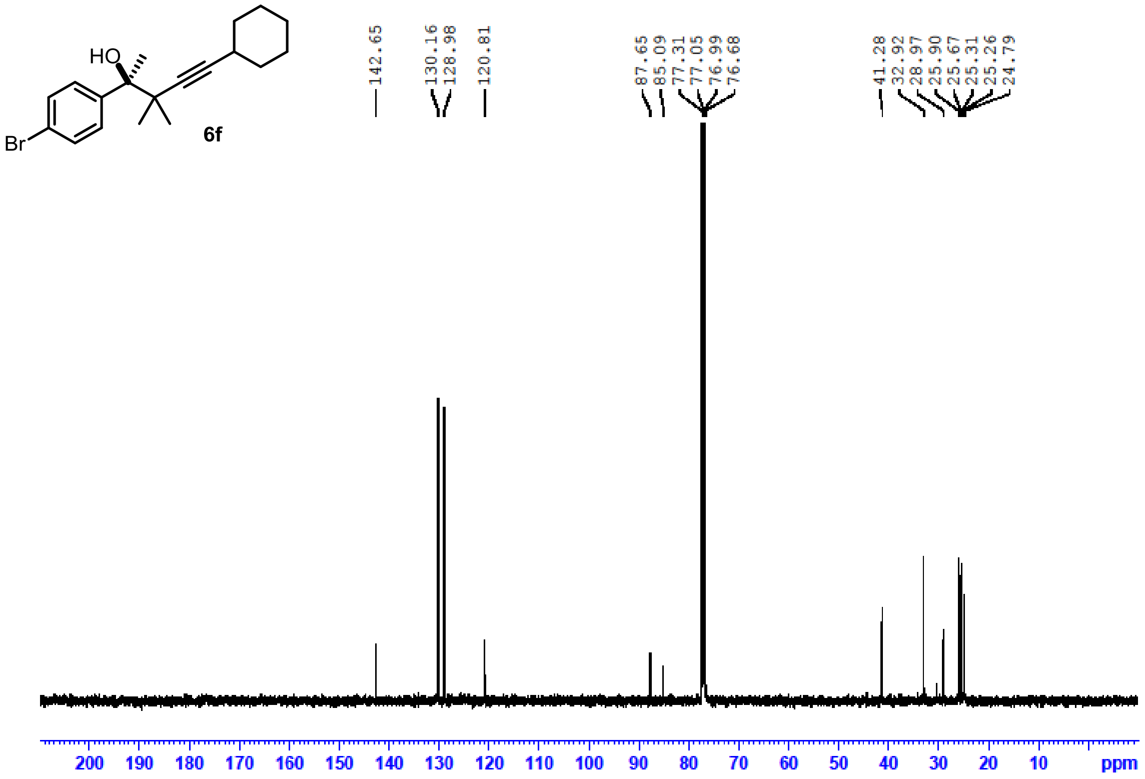

Spectra for **6g:** ^{1}H NMR (400 MHz, $CDCl_3$):

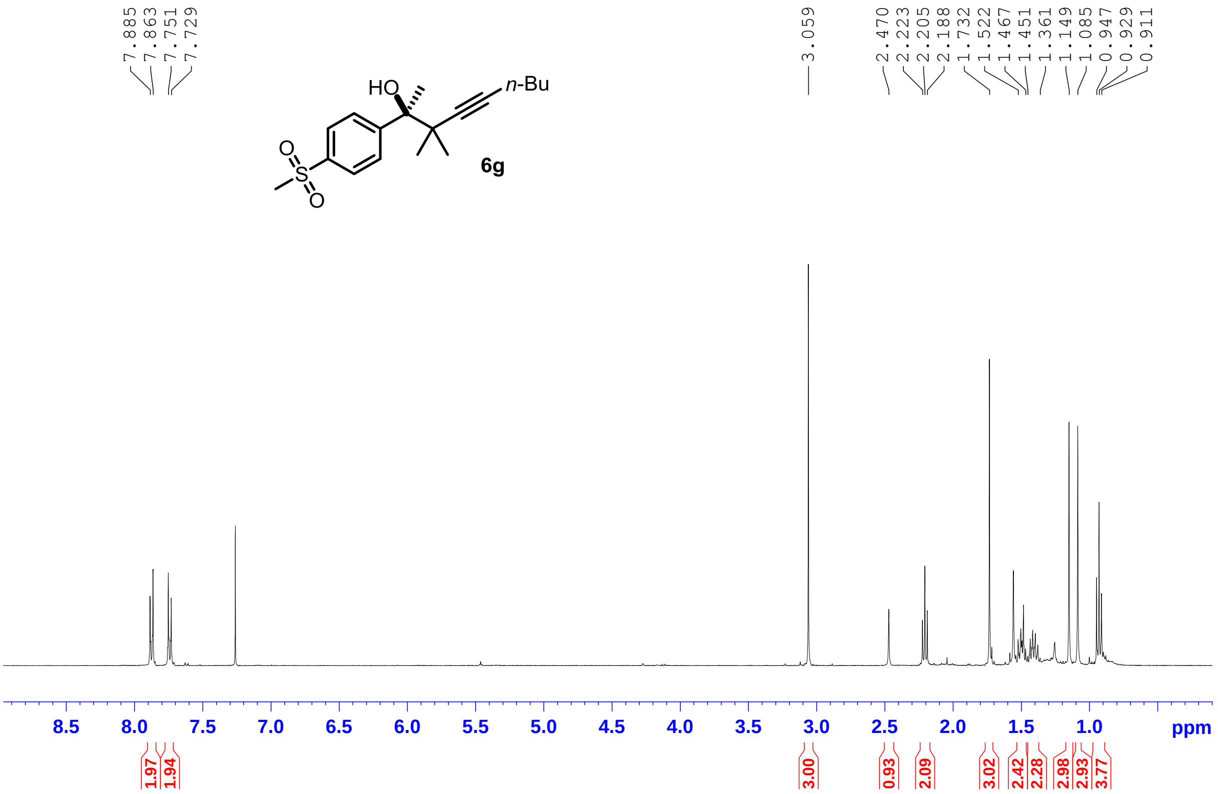

^{13}C NMR (100 MHz, $CDCl_3$):

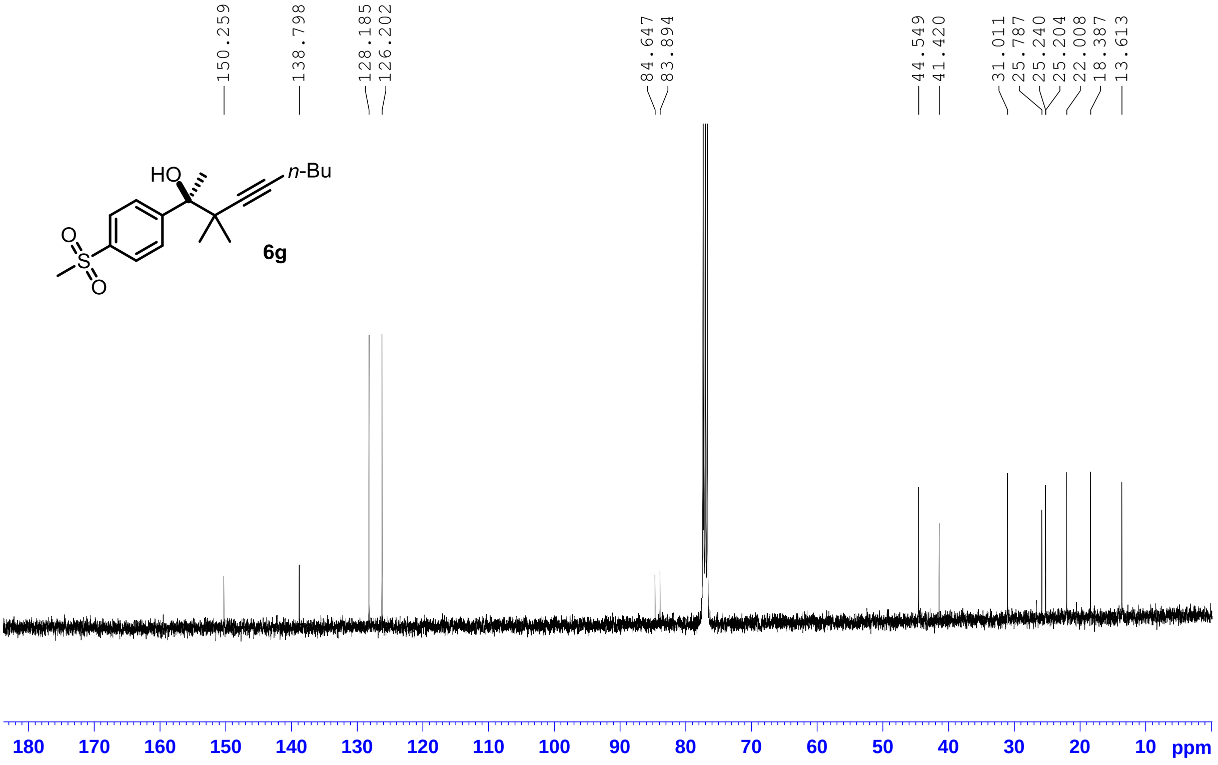

Spectra for **6h:** ^{1}H NMR (400 MHz, $CDCl_3$):

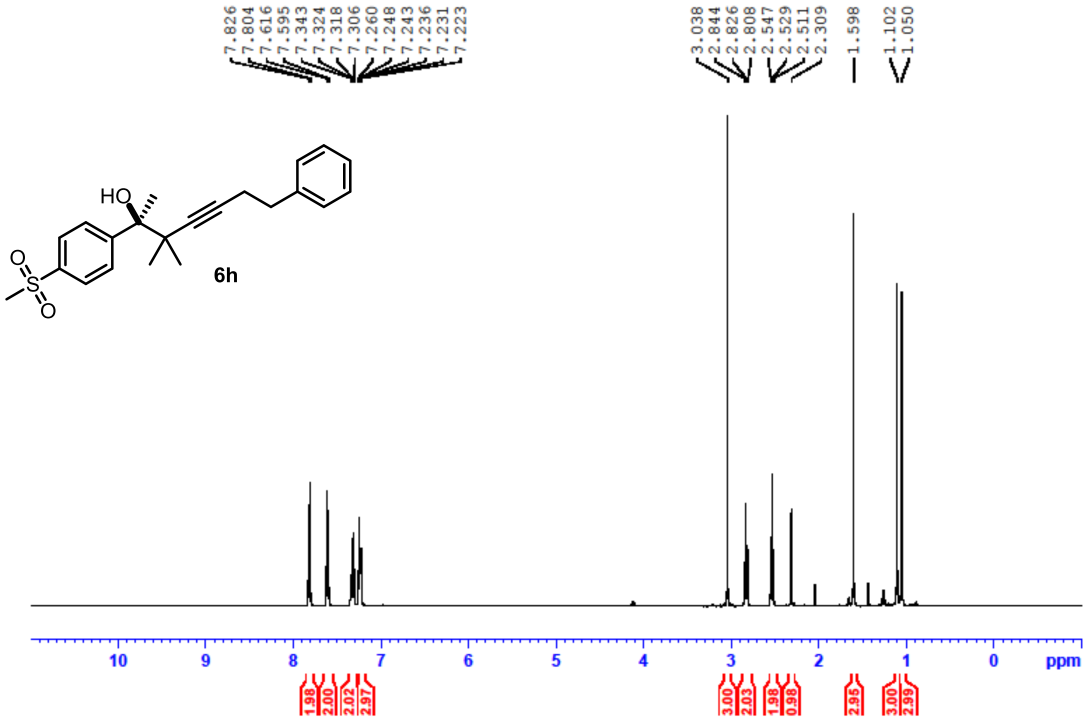

^{13}C NMR (100 MHz, $CDCl_3$):

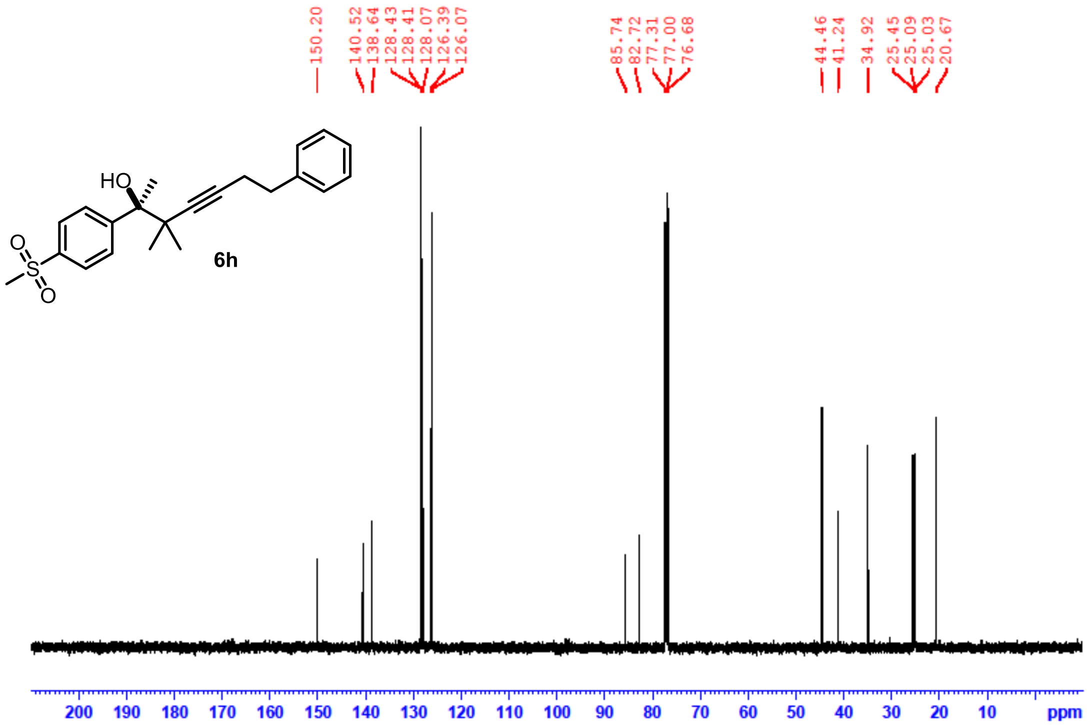

Spectra for **6i:** ^{1}H NMR (400 MHz, $CDCl_3$):

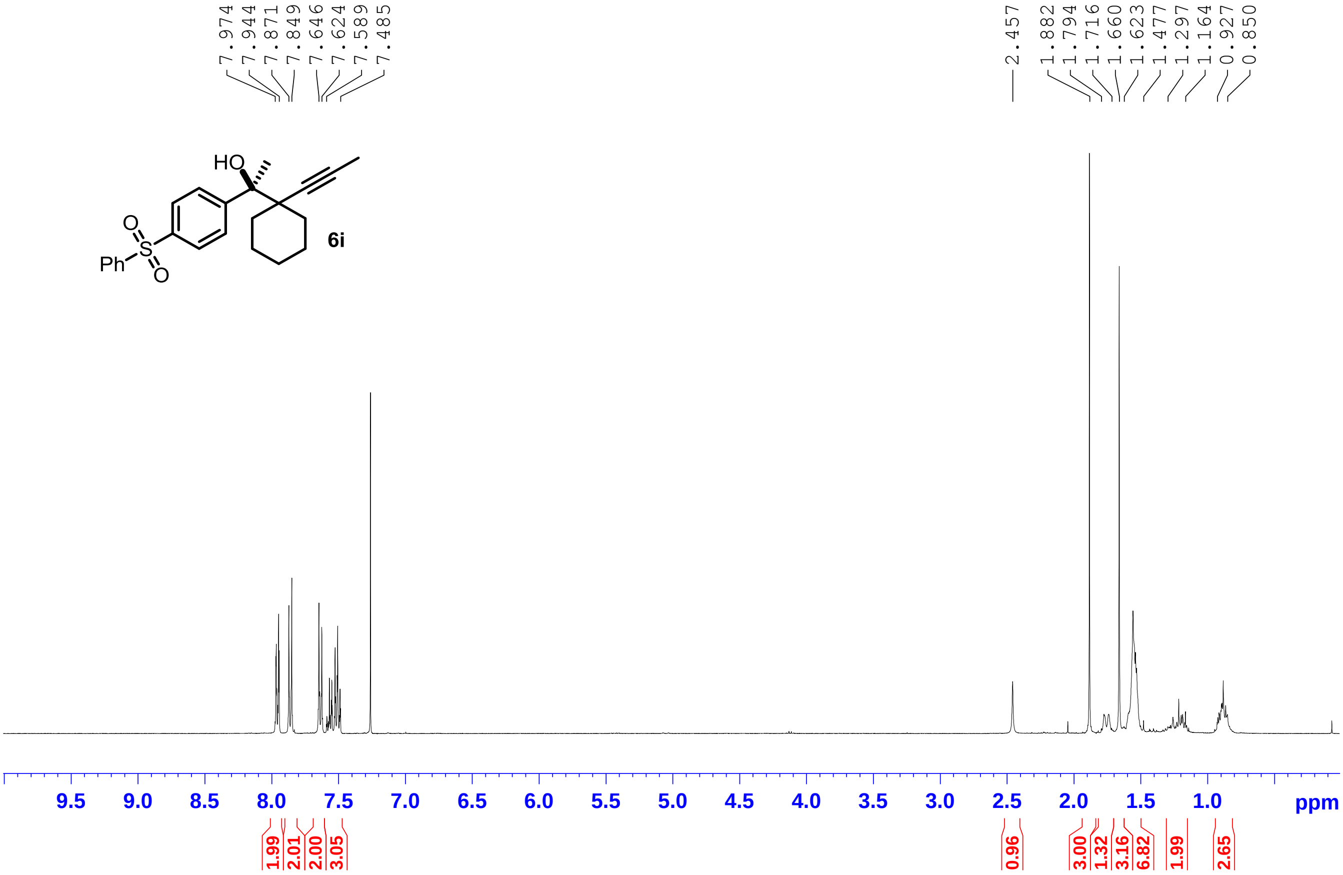

^{13}C NMR (100 MHz, $CDCl_3$):

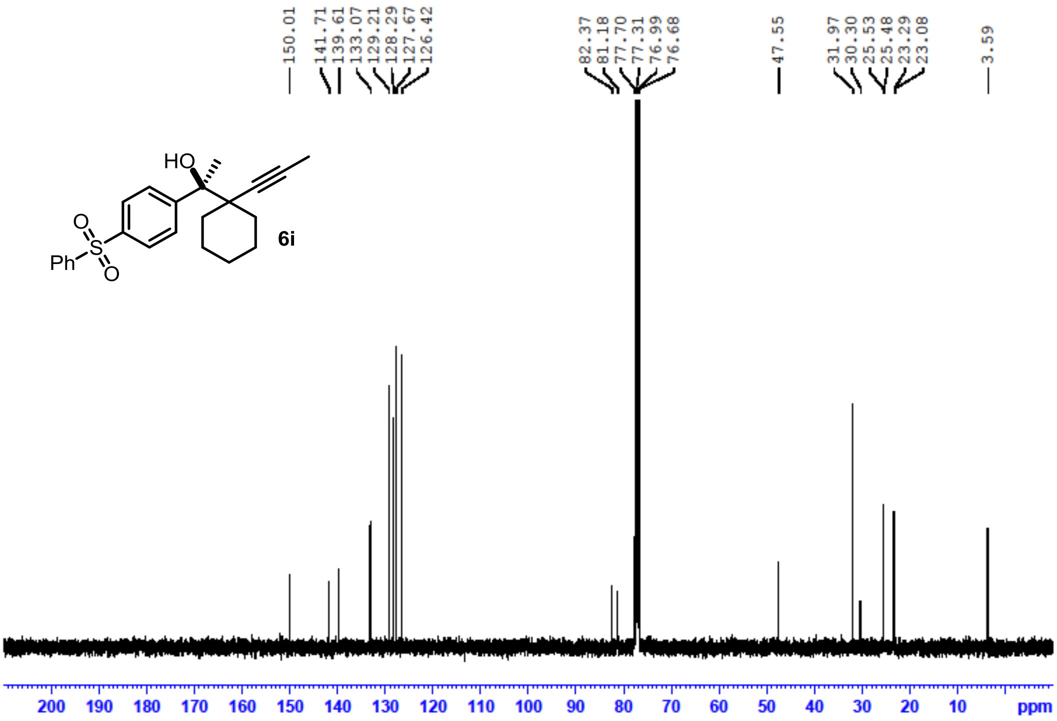

Spectra for **7a:** ^{1}H NMR (400 MHz, $CDCl_3$):

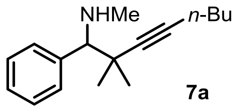

7a

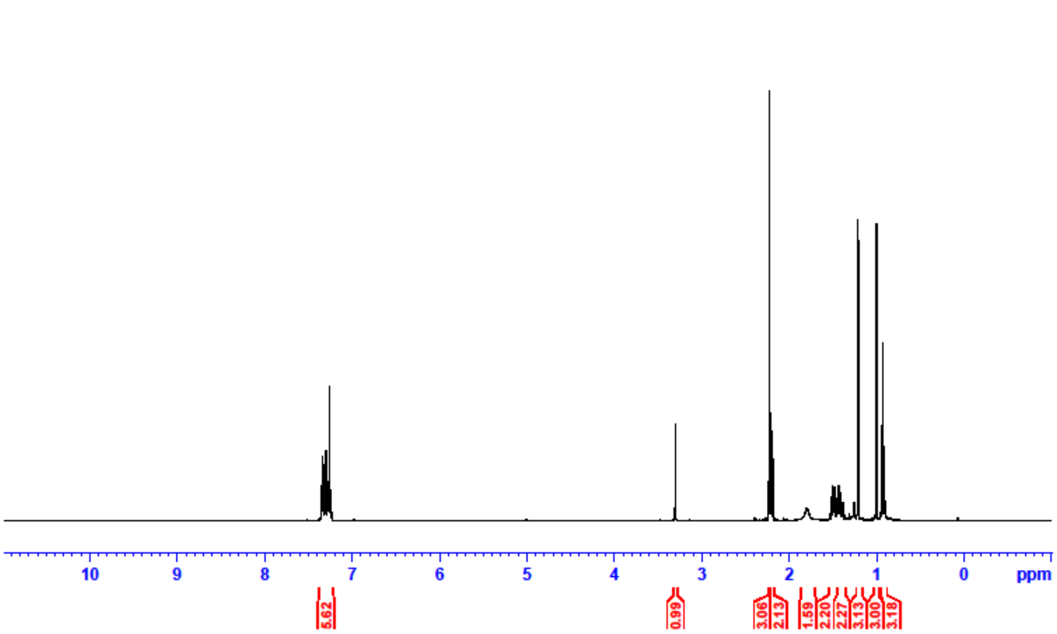

^{13}C NMR (100 MHz, $CDCl_3$):

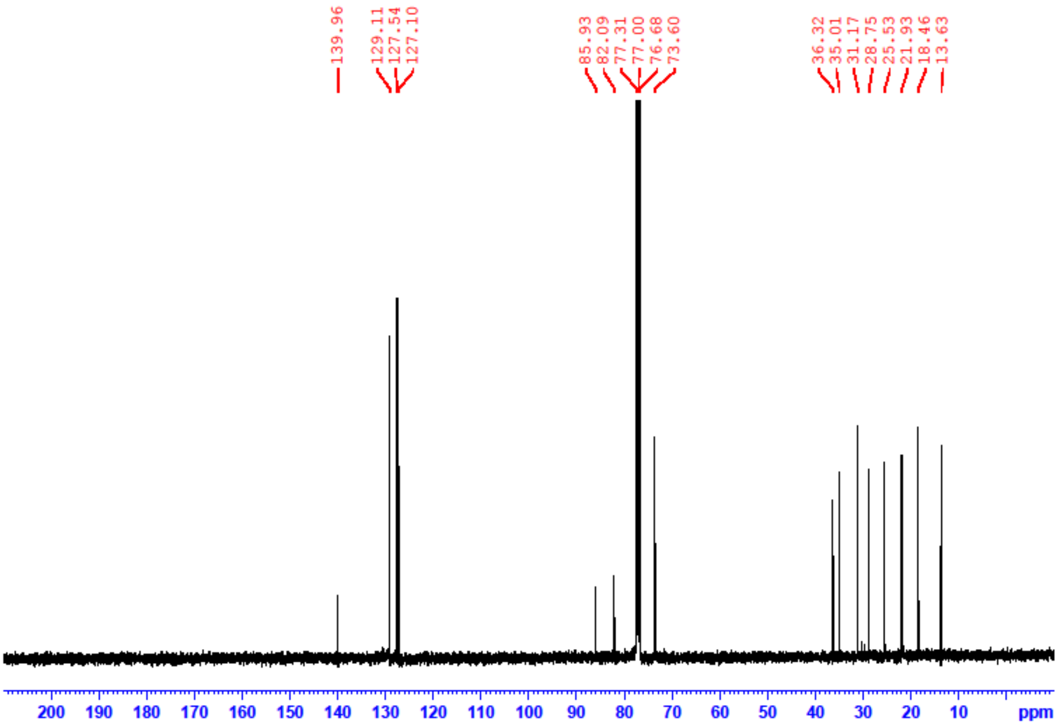

Spectra for **7b:** ^{1}H NMR (400 MHz, $CDCl_3$):

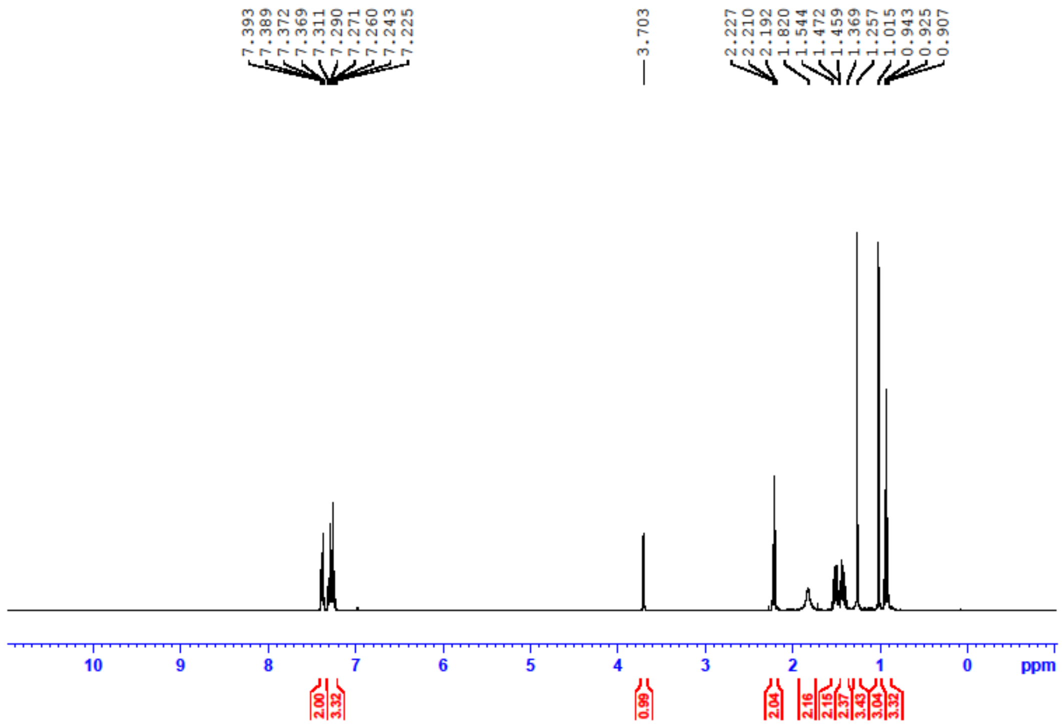

^{13}C NMR (100 MHz, $CDCl_3$):

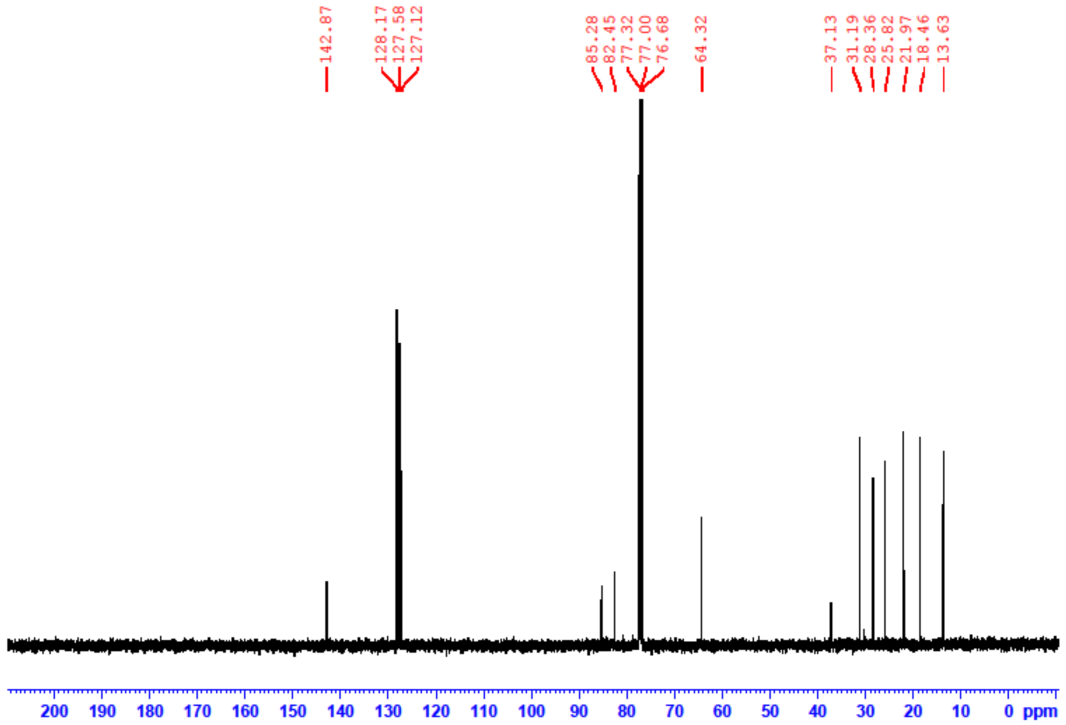

Spectra for **7c:** ^{1}H NMR (400 MHz, $CDCl_3$):

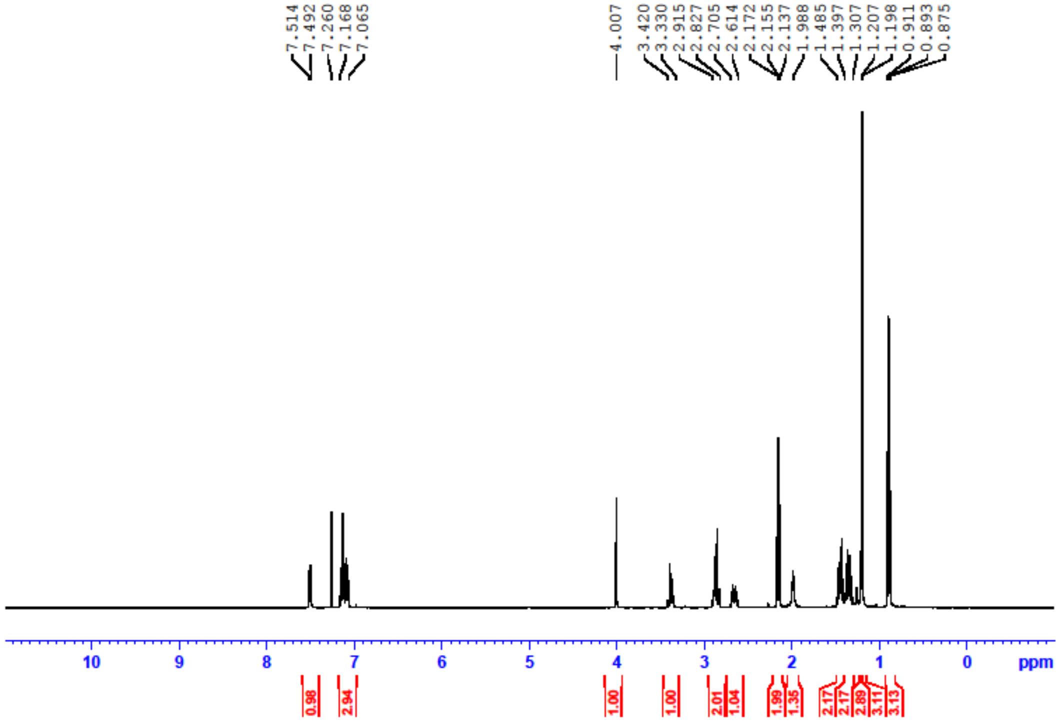

^{13}C NMR (100 MHz, $CDCl_3$):

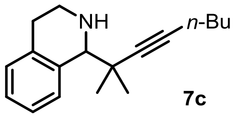

7c

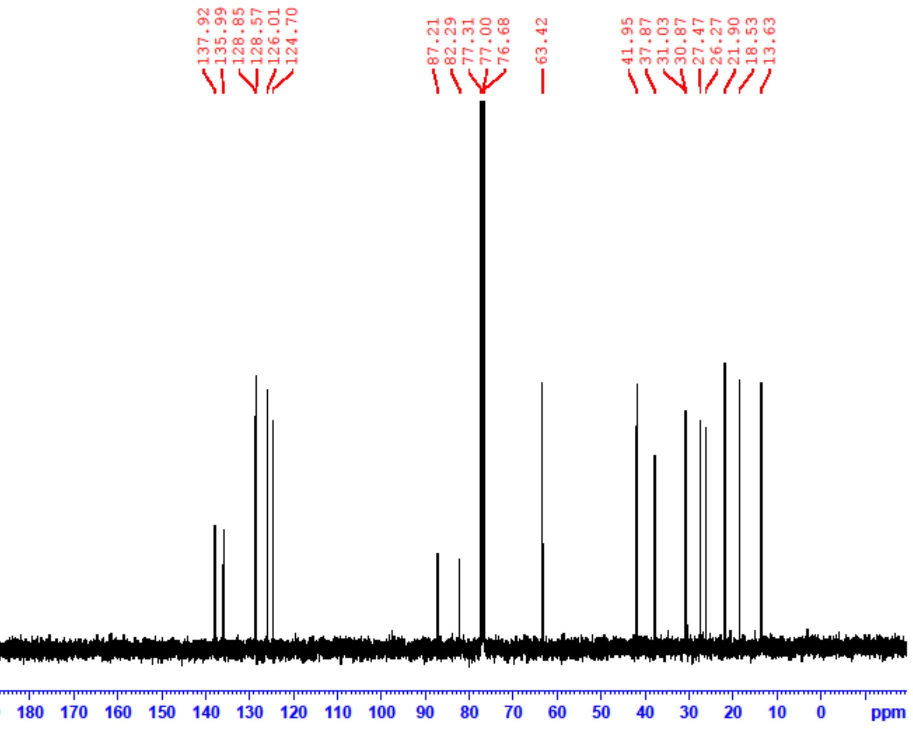

Spectra for **7d:** ^{1}H NMR (400 MHz, CD_3CN):

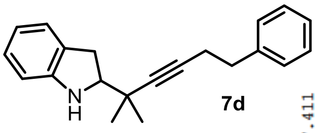

7d

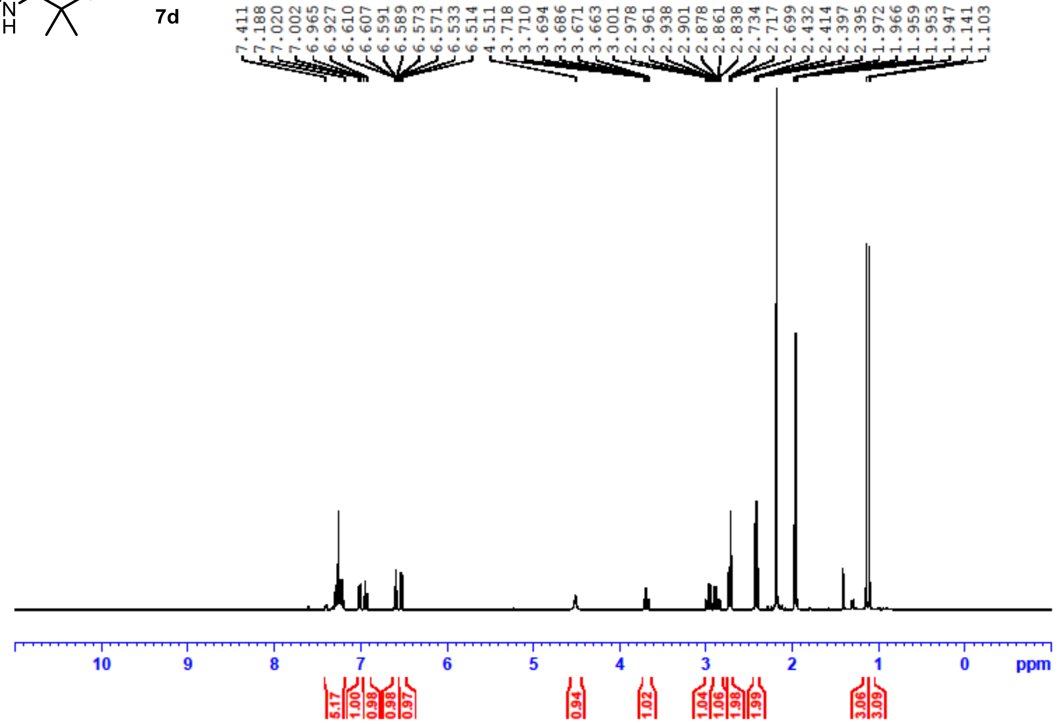

^{13}C NMR (100 MHz, CD_3CN):

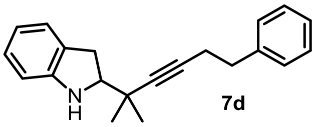

7d

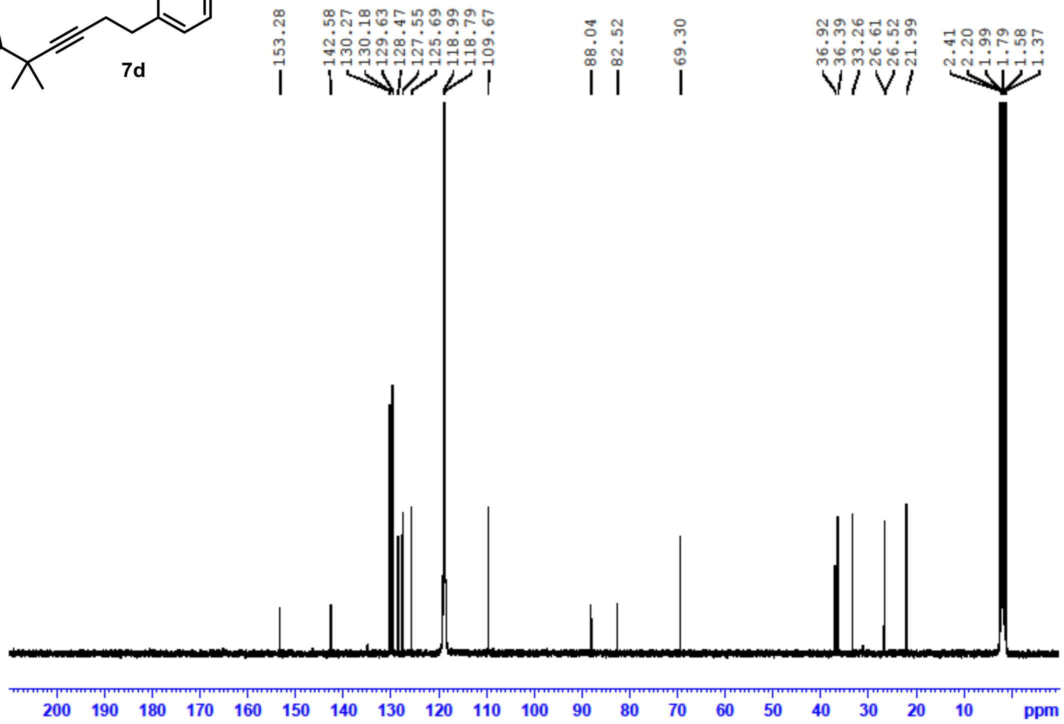

Spectra for **7e:** ^{1}H NMR (400 MHz, $CDCl_3$):

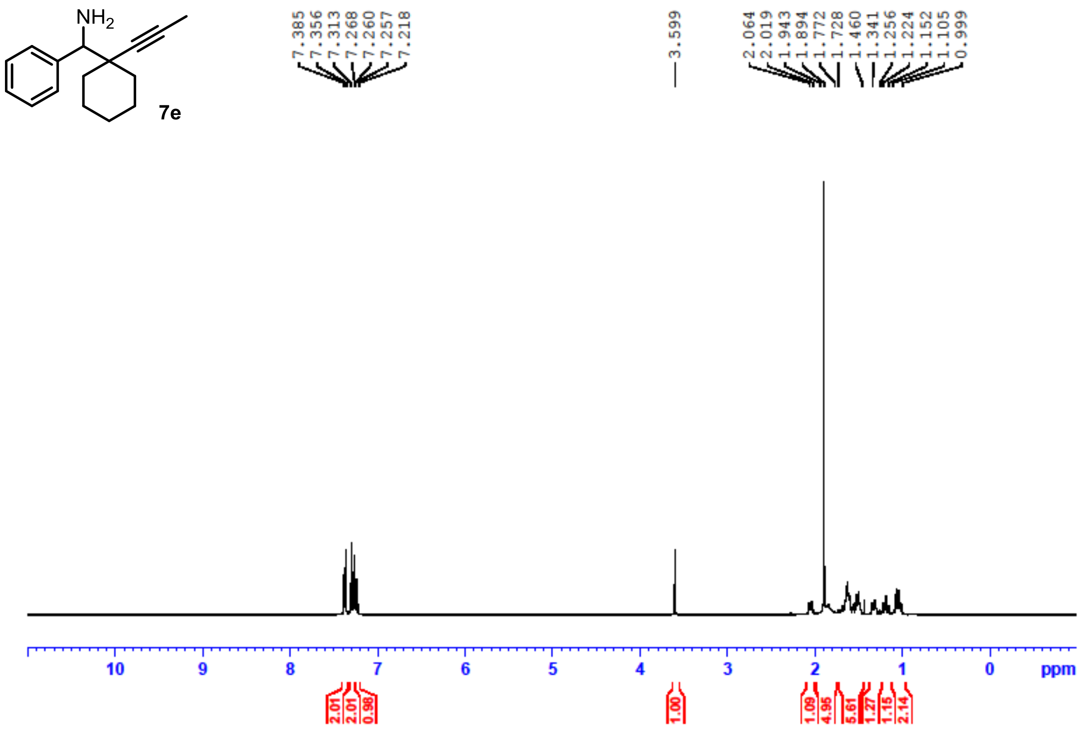

^{13}C NMR (100 MHz, $CDCl_3$):

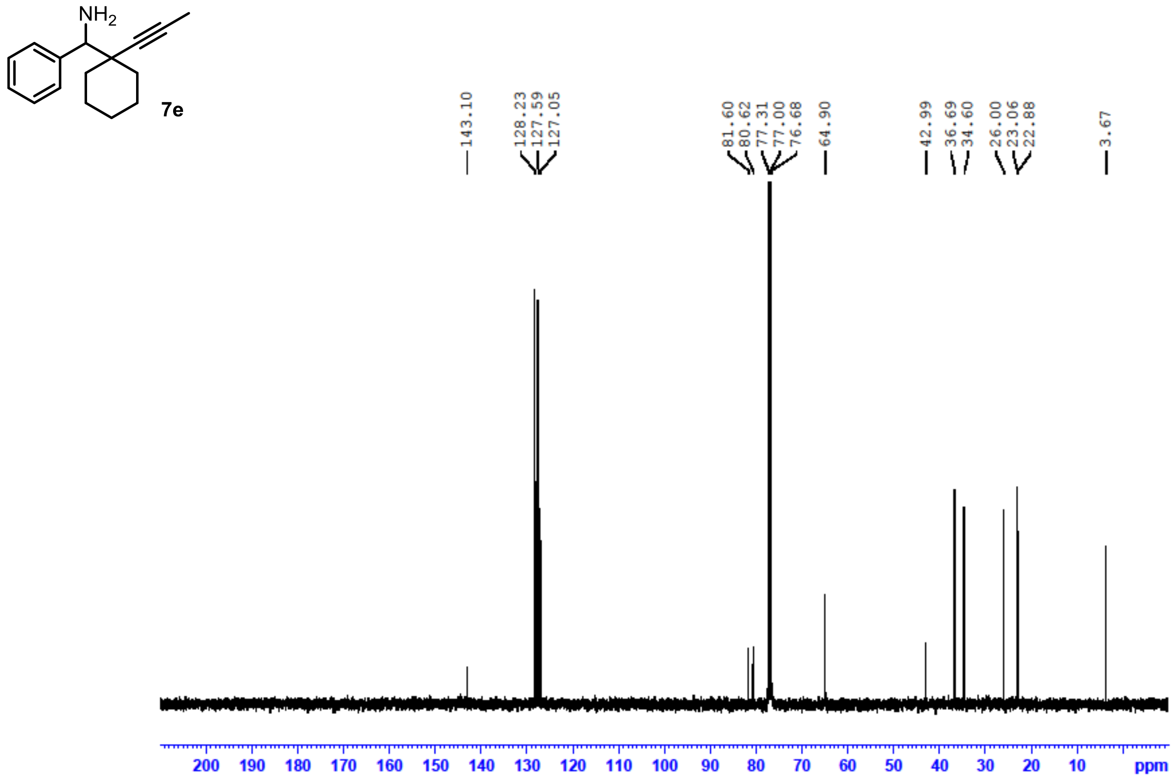

Spectra for **9:** ^{1}H NMR (400 MHz, $CDCl_3$):

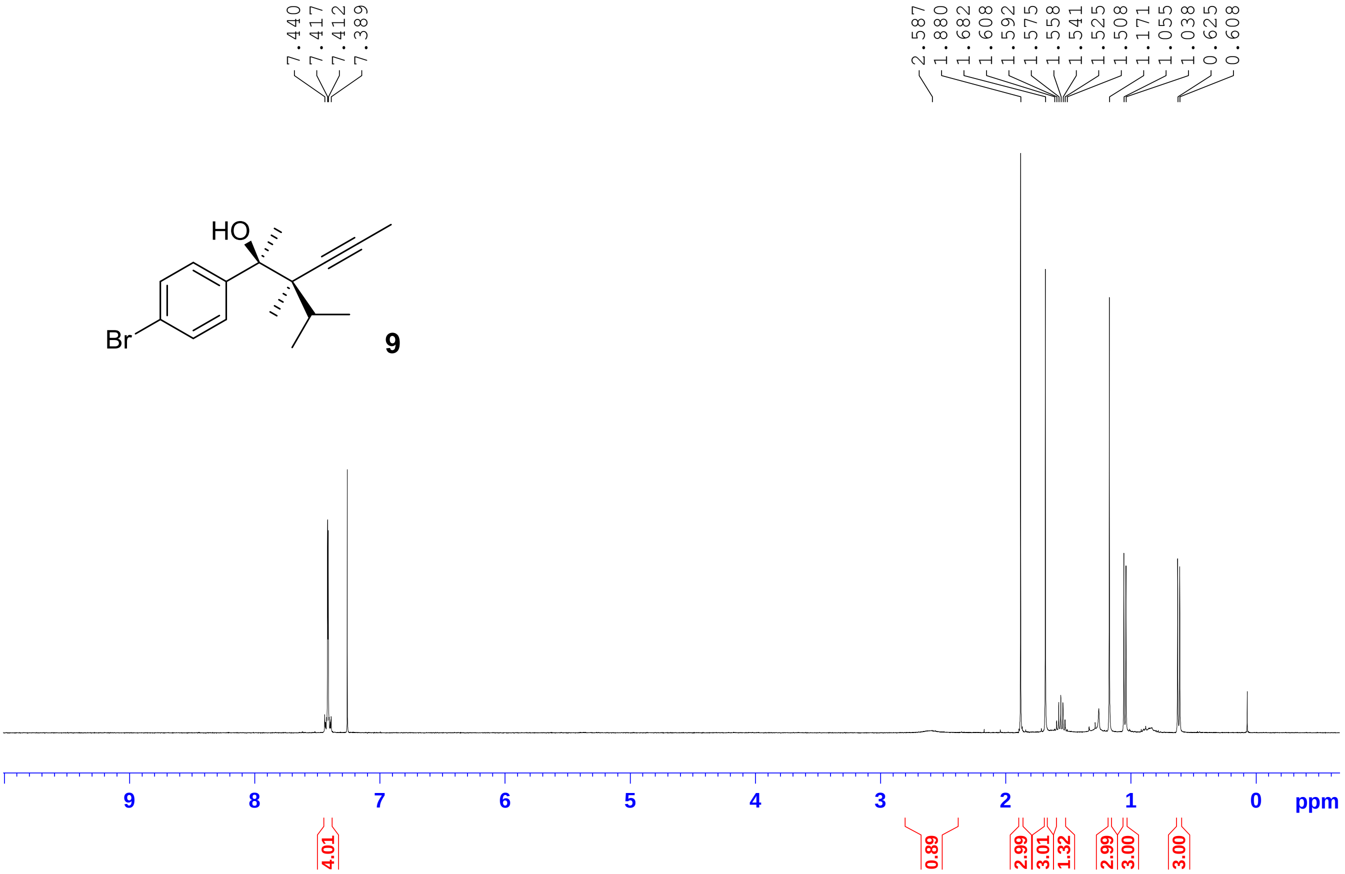

^{13}C NMR (100 MHz, $CDCl_3$):

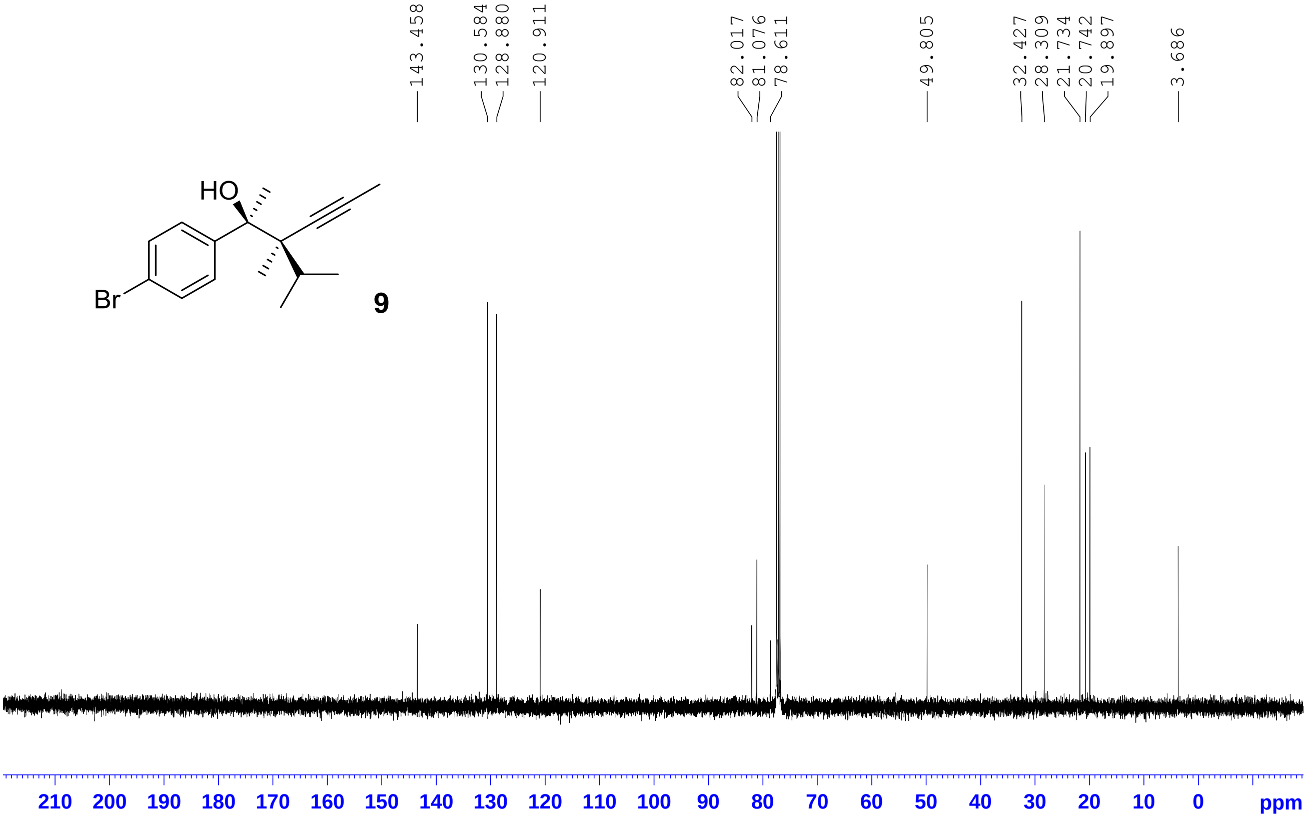

Spectra for **6g-ester:** ^{1}H NMR (400 MHz, $CDCl_3$):

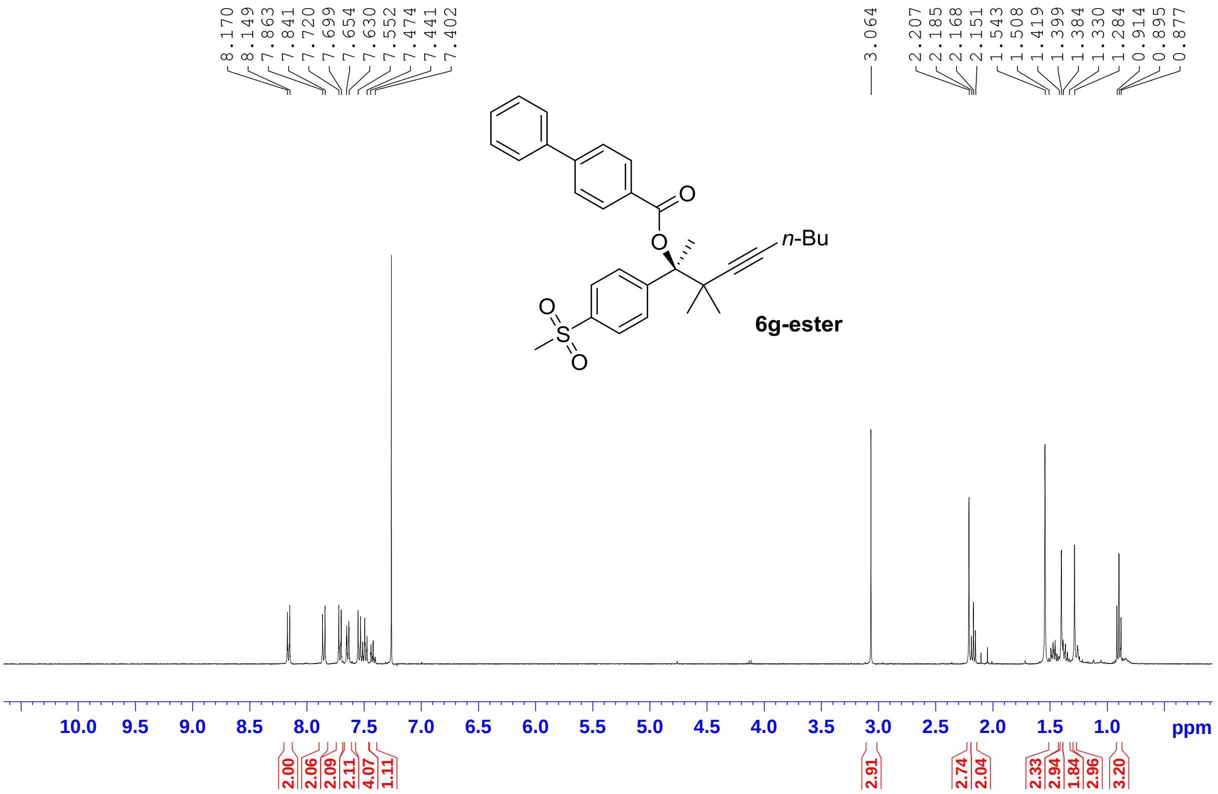

^{13}C NMR (100 MHz, $CDCl_3$):

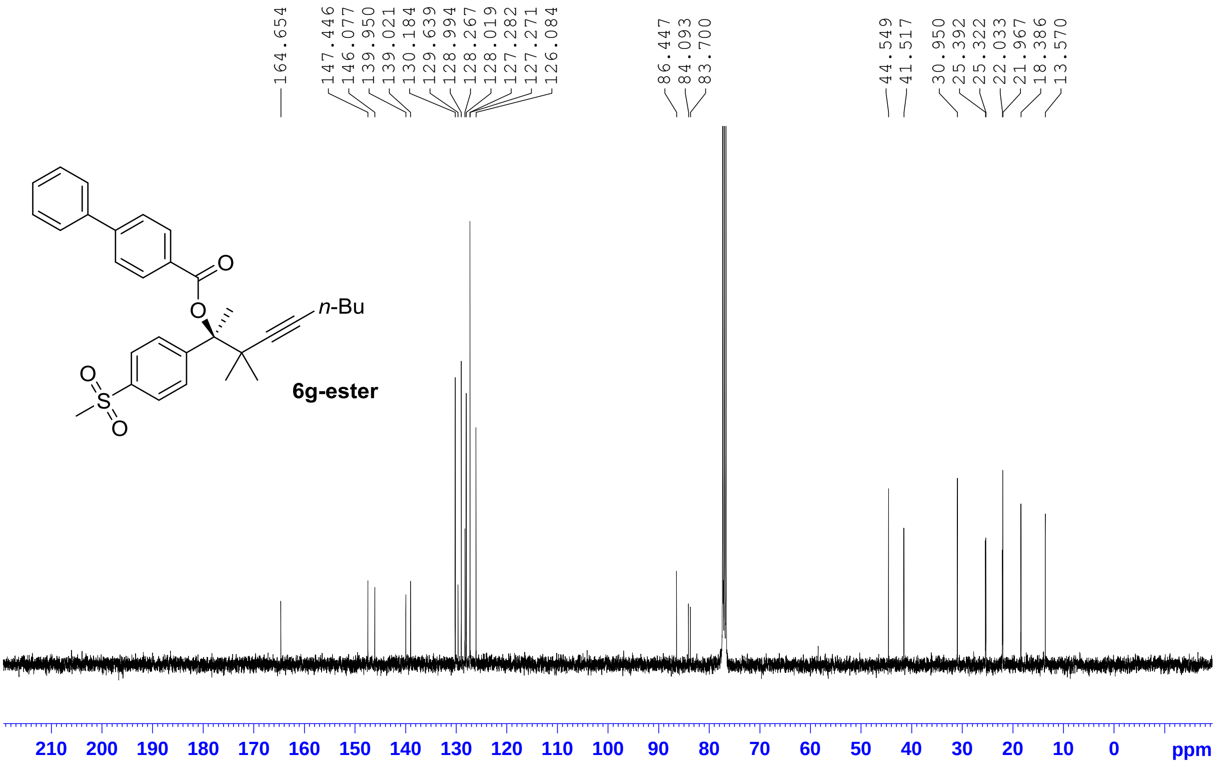

Supplement: Supplementary file 1 [file SC-009-C7SC05123A-s001.pdf]
